# Biotransformation of Thiochroman Derivatives Using Marine-Derived Fungi: Isolation, Characterization, and Antimicrobial Activity

**DOI:** 10.3390/ijms26030908

**Published:** 2025-01-22

**Authors:** Jorge R. Virués-Segovia, Cristina Pinedo-Rivilla, Salvador Muñoz-Mira, Matilde Ansino, Victoria E. González-Rodríguez, Abdellah Ezzanad, Fátima Galán-Sánchez, Rosa Durán-Patrón, Josefina Aleu

**Affiliations:** 1 Departamento de Química Orgánica, Facultad de Ciencias, Universidad de Cádiz, 11510 Puerto Real, Spain; jorge.roca@uca.es (J.R.V.-S.); cristina.pinedo@uca.es (C.P.-R.); salva.munozmira@alum.uca.es (S.M.-M.); ma.ansinoor@alum.uca.es (M.A.); abdellah.ezzanad@gm.uca.es (A.E.); 2 Instituto de Investigación en Biomoléculas (INBIO), Universidad de Cádiz, 11510 Puerto Real, Spain; 3 Laboratorio de Microbiología, Departamento de Biomedicina, Biotecnología y Salud Pública, Facultad de Ciencias del Mar y Ambientales, Universidad de Cádiz, 11510 Puerto Real, Spain; victoriaeugenia.gonzalez@uca.es; 4 Servicio de Microbiología, Hospital Universitario Puerta del Mar, 11009 Cádiz, Spain; fatima.galan@uca.es; 5 Instituto de investigación e Innovación Biomédica de Cádiz (INIBICA), 11009 Cádiz, Spain

**Keywords:** marine fungi, biotransformation, *Emericellopsis maritima*, *Purpureocillium lilacinum*, thiochromanone, antimicrobial activity

## Abstract

Thiochroman derivatives are highly versatile molecules widely used for the synthesis of novel heterocycles and bioactive compounds. In our study, we conducted the biotransformation of thiochroman-4-ol (**1**) and 6-chlorothiochroman-4-ol (**1a**) using the marine-derived fungal strains *Emericellopsis maritima* BC17 and *Purpureocillium lilacinum* BC17-2. Biotransformations yielded ten known thiochroman derivatives along with the compound 1-(5-chloro-2-(methylthio)phenyl)propane-1,3-diol (**6a**), which was described for the first time. Moreover, we successfully characterized the stereoisomers of sulfoxides **3** and **3a**. Their structures and absolute configurations were established though comprehensive analyses of NMR, HR ESI-MS, and ECD spectra, as well as by using Mosher’s method. Antimicrobial activity of the isolated metabolites was evaluated against bacterial and fungal human pathogens, specifically *Staphylococcus aureus* ATCC 29213, *Escherichia coli* ATCC25922, and *Candida albicans* HPM-1922816.

## 1. Introduction

Over the past few decades, biocatalysis has emerged as an increasingly valuable tool for the chemical synthesis of novel derivatives of drugs, agrochemicals, and fragrances with enhanced properties. Biocatalysis provides an alternative means of producing precursor and intermediate molecules used in manufacturing processes. This is due to the capacity of biological systems to conduct regio- and stereoselective chemical reactions that cannot be replicated using conventional synthetic methods. Furthermore, biocatalysis is an attractive option for Green Chemistry applications as it employs mild and often less expensive reaction conditions, such as water as the reaction medium and operating at physiological pH and temperature levels [1].

Marine-derived fungi, naturally adapted to the extreme conditions of the oceanic environment, represent a promising reservoir of novel enzymes with unique properties. These microorganisms have evolved to thrive under ecological stress, resulting in metabolic adaptations that endow their enzymes with remarkable properties, including salt tolerance, hyperthermostability, barophilicity, and cold adaptability. However, despite their widespread distribution and abundance, marine fungi remain an underexplored resource whose enzymatic potential holds significant promise for applications in biotransformation and biodegradation, offering potential solutions to industrial and environmental challenges [2].

The recent isolation of *Emericellopsis maritima* BC17 and *Purpureocillium lilacinum* BC17-2 from the intertidal region of Bay of Cádiz, Spain, offers a valuable opportunity to assess their ability to biotransform a range of substrates. It is worth noting that the biocatalytic potential of the genera *Emericellopsis* and *Purpureocillium* has not yet been fully evaluated. Recent studies have only described the biodegradation potential of the genus *Emericellopsis* in lignocellulose and oxytetracycline and aflatoxin antibiotics [3,4,5]. Furthermore, the capacity of *P. lilacinum* to biodegrade toluene, phthalate plasticizers, or solid waste from the leather industry has also been documented recently [6,7,8].

Thiochroman derivatives are versatile reagents widely utilized in the heterocyclic synthesis of novel compounds and represent a privileged scaffold in drug design and organic synthesis [9,10]. Consequently, the potential biocatalytic of these fungi offers a promising alternative to traditional chemical synthesis for the production of thiochromanoid derivatives. Numerous derivatives with this scaffold have demonstrated notable biological and pharmacological properties, including antimicrobial [10,11,12,13,14,15], cytotoxic [16,17,18,19], antiviral [20], and even antileishmanial activities [21,22,23]. However, despite their promising pharmacological properties, the chemistry of thiochromonoids remains relatively underexplored.

For instance, Xiao et al. studied the design and synthesis of antifungal compounds using thiochromanone as a lead compound [13]. The products exhibited a higher level of inhibition of the phytopatogen *Botryosphaeria dothidea* than the commercial antifungal Pyrimethanil [13]. Similarly, the antifungal activity of thiochromanone derivatives was investigated by Pinedo-Rivilla et al. [15]. The compounds 6-methylthiochroman-4-one, 6-chlorothiochroman-4-one, and 6-methylthiochroman-4-ol displayed total inhibition rates of 96–100% at concentrations ranging from 100 to 250 μg/mL against the phytopathogenic fungus *Botrytis cinerea* [15]. Other derivatives have shown antimicrobial activities against Gram-positive bacteria, including *Staphylococcus aureus*, *Staphylococcus epidermidis*, and *Bacillus pumilus*, and Gram-negative bacteria, such as *Salmonella typhi* and *Pseudomonas aeruginosa* [24].

The biotransformation of thiochroman derivatives has been little studied, as evidenced by the available literature. Thiochroman-4-ol (**1**) has been biotransformed by the fungi *Mortierella isabellina* ATCC 42613 and *Helminthosporium* sp. NRRL 4671, yielding thiochroman-4-one (**2**) and recovering the unreacted *R* alcohol with high enantiomeric purity. In the same study, related compounds such as chroman-4-one and thiochroman-4-one were converted into thiochroman-4-ols in good yields and enantioselectivity [25]. Furthermore, Pinedo-Rivilla et al. investigated the biotransformation of the derivatives 6-chlorothiochroman-4-one and 6-methylthiochroman-4-one by the fungi *Trichoderma viride* and *Botrytis cinerea* [15]. Their findings revealed the production of thiochromanols, sulfoxides, and sulfones with good yields and enantioselectivities [15].

In this study, we describe the biotransformation of the substrates thiochroman-4-ol (**1**) and 6-chlorothiochroman-4-ol (**1a**) by the marine-derived strains *E. maritima* BC17 and *P. lilacinum* BC17-2. The strain BC17-2, which was isolated and identified in the present study for the first time in the Bay of Cadiz, Spain, yielded the new compound (*S*)-1-(5-chloro-2-(methylthio)phenyl)propane-1,3-diol (**6a**). Given the bioactive potential of this scaffold, the biotransformation products were examined for antimicrobial activities against the pathogenic bacteria *Staphylococcus aureus* ATCC29213 and *Escherichia coli* ATCC25922, as well as the clinical isolate *Candida albicans* HPM-1922816.

## 2. Results and Discussion

Fungal strains BC17 [26] and BC17-2 were isolated from surface sediments collected in the intertidal zone of the inner Bay of Cadiz (Cádiz, Spain). The strain BC17-2 was identified as *Purpureocillium lilacinum*, using morphological and molecular methods, by the identification service of the Spanish Type Culture Collection (CECT, https://www.uv.es/cect, accessed on 7 December 2023). The isolate exhibits the macroscopic and microscopic characteristics typical of the species *P. lilacinum* [27].

**Figure 1:**
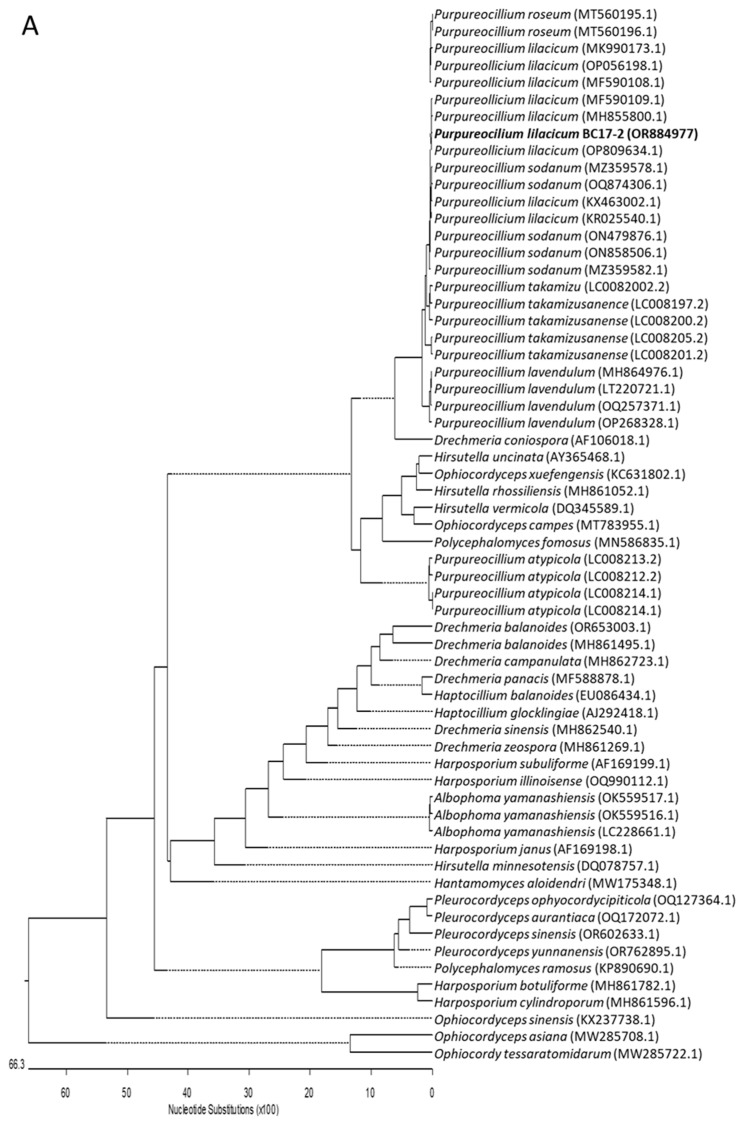

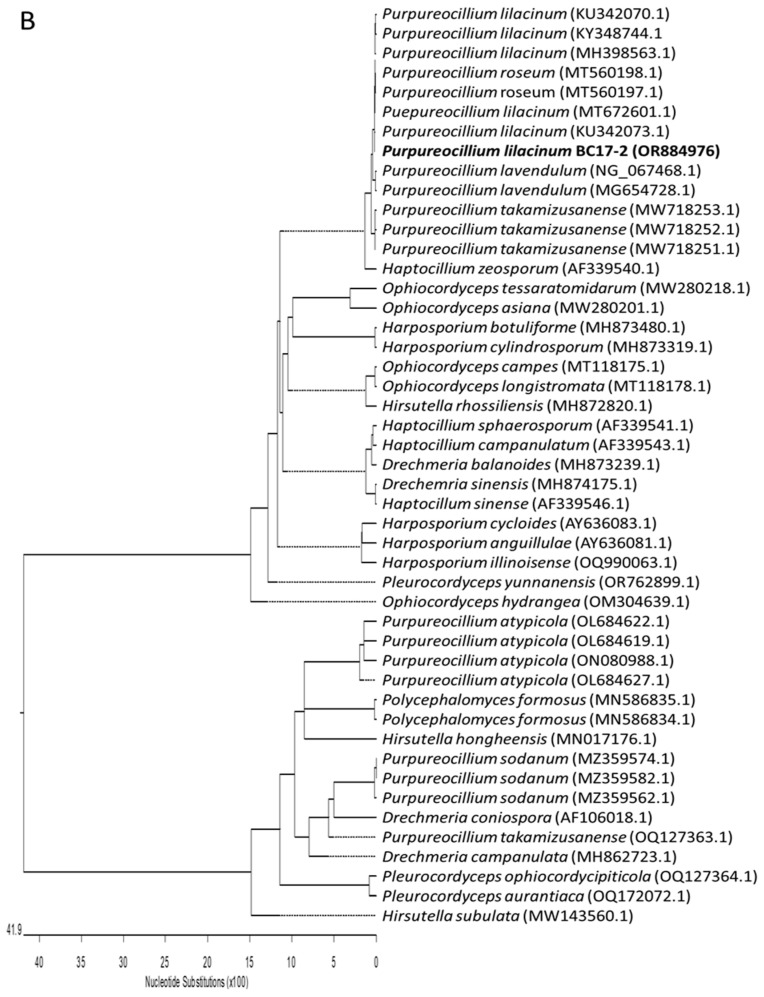
Neighbor-joining trees constructed using (**A**) intergenic spaces ITS1 and ITS2, including the 5.8S rRNA; (**B**) 28S rRNA gene. Sequences identified in this study are highlighted in bold, and published sequences obtained from the GenBank database. The length of each branch pair reflects the distance between respective sequence pairs. A dotted line on the tree denotes a negative branch length, while the bar indicates the number of nucleotide substitutions.

Neighbor-joining phylogenetic analysis was conducted using the Kimura two-parameter model and a bootstrap test with 5000 runs (MegAlign, DNASTAR^®^ Lasergene package v.7.1.0). Sequences of related fungal species/genus from the family *Ophiocordycipitaceae* were downloaded from the GenBank database. The phylogenetic trees shown in Figure 1 were constructed using (i) sixty-two sequences, including nine genera and thirty-seven species, for the ribosomal DNA region comprising the intergenic spaces ITS1 and ITS2, including the 5.8S rRNA (Figure 1A); and (ii) forty-seven sequences, including eight genera and thirty-one species, for the 28S rRNA gene (Figure 1B). Based on all these studies, it was determined that strain BC17-2 is clearly grouped with the species *P. lilacinum* (Figure 1).

Racemic alcohols thiochroman-4-ol (**1**) and 6-chlorothiochroman-4-ol (**1a**) were obtained in good yields from the commercially available ketones thiochroman-4-one (**2**) and 6-chlorothiochroman-4-one (**2a**), respectively, by reduction with NaBH_4_. Alcohols were identified by comparison of their spectroscopic data with those reported in the literature [28,29].

The substrates **1** and **1a** were incubated separately with the marine sediment-derived fungi *P. lilacinum* BC17-2 and *E. maritima* BC17. The structures of the biotransformation products were established through analysis of their 1D and 2D NMR and HR ESI-MS spectra and by comparison of their spectroscopic data with those reported in the literature. Their absolute configurations were determined either by comparing their optical activities with those published in the literature or by using Mosher’s method [30] and electronic circular dichroism (ECD) calculations. Additionally, the diastereomeric and enantiomeric excesses were determined by HPLC analysis using a Chiralcel IB N-5 column.

### 2.1. Biotransformation of (±)-Thiochroman-4-Ol (**1**) and (±)-6-Chlorothiochroman-4-ol (**1a**) by P. lilacinum BC17-2

**Figure 2:**
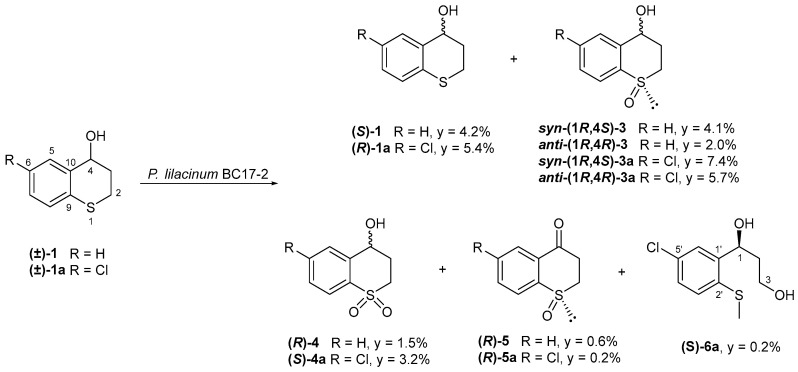
Biotransformation of thiochroman-4-ol (**1**) and 6-chlorothiochroman-4-ol (**1a**) by *P. lilacinum* BC17-2. Yields (y) are expressed in % of mol products/mol substrates.

In addition to the starting material, the biotransformation of (±)-thiochroman-4-ol (**1**) by *P. lilacinum* BC17-2 afforded four known compounds: *syn*-(1*R*,4*S*)-(–)-thiochroman-4-ol 1-oxide [***syn*-(1*R*,4*S*)-3**] [31], *anti*-(1*R*,4*R*)-(–)-thiochroman-4-ol 1-oxide [***anti*-(1*R*,4*R*)-3**] [31], (*R*)-(–)-thiochroman-4-ol 1,1-dioxide [**(*R*)-4**] [32], and (*R*)-(–)-thiochroman-4-one 1-oxide [**(*R*)-5**] [33] (Figure 2). The starting material recovered exhibited ${\left[\alpha \right]}_{D\;}^{20}\;$= −4.1. A comparison of its optical activity with that reported for both enantiomers in the literature allowed us to assign its absolute configuration as *S* [28,34]. However, the *ee* was found to be 1.6%, indicating that the substrate remains almost racemic.

On the other hand, the biotransformation of (±)-6-chlorothiochroman-4-ol (**1a**) by the strain *P. lilacinum* BC17-2 yielded five compounds, including *syn*-(1*R*,4*S*)-(–)-6-chlorothiochroman-4-ol 1-oxide [***syn*-(1*R*,4*S*)-3a**], *anti*-(1*R*,4*R*)-(–)-6-chlorothiochroman-4-ol 1-oxide [***anti*-(1*R*,4*R*)-3a**], (*S*)-(+)-6-chlorothiochroman-4-ol 1,1-dioxide [**(*S*)-4a**], and (*R*)-(–)-6-chlorothiochroman-4-one 1-oxide [**(*R*)-5a**], together with (*S*)-(+)-1-(5-chloro-2-(methylthio)phenyl)propane-1,3-diol [**(*S*)-6a**], which is described here for the first time. The starting material recovered was assigned an *R*-configuration by comparing its specific rotation value with literature data (Figure 2) [29].

Sulfoxides **3** and **3a** were obtained as a mixture of diastereoisomers, which were separated by preparative thin-layer chromatography in the corresponding *syn* and *anti* diastereoisomers. The diastereoisomeric excesses of the sulfoxides (**3** and **3a**) were 31.5% and 17.4%, respectively, in favor of the *syn*-configuration in both cases. The *syn*-configuration was assigned on the basis of the chemical shift for H-4, which exhibits an upfield shift in the *syn*-diastereoisomer [15]. The absolute configuration at C-4 of the major enantiomers of the sulfoxides***syn***-**3**, ***anti*-3**, and ***syn*-3a** was deduced by Mosher’s method. In order to achieve this, the fractions containing the aforementioned sulfoxides were submitted to a separation by HPLC using a Chiralcel IB N-5 column and *n*-hexane:iPrOH 93:7. Subsequently, treatment of the major enantiomers with (*R*)-α-methoxyphenyl acetic acid (*R*-MPA) and/or (*S*)-α-methoxyphenyl acetic acid (*S*-MPA), separately, yielded the corresponding (*R*)- and (*S*)-MPA esters at C-4.

In accordance with this method, a comparison of the chemical shifts in the ^1^H NMR spectra of the two MPA esters of ***syn*-3** (Figures S7 and S10) revealed a positive value of Δ*δ^RS^* for the vicinal proton H-5 (+0.70 ppm) and negative values of Δ*δ^RS^* for H-3a (−0.38 ppm), H-3b (−0.24), H-2a (−0.16), and H-2b (−0.17). These data indicated an *S* configuration for C-4 in ***syn***-**3**. Consequently, its structure was assigned as *syn*-(1*R*,4*S*)-(–)-thiochroman-4-ol 1-oxide. This compound was produced by *P. lilacinum* with a low diastereoselectivity (31.5% *de*) and a moderate enantioselectivity (52.7% *ee*).

The absolute C-4 configuration of the major enantiomer of the sulfoxide ***anti*-3** was assigned by single derivatization and low-temperature NMR spectroscopy. According to this method, a comparison of the ^1^H NMR spectra of the (*S*)-MPA ester recorded at room temperature (T_1_) (Figure S13) and at −25 °C (T_2_) (Figure S16) showed a positive value of Δ*δ*^T1T2^ (*δ*T_1_−*δ*T_2_) for the protons H-2b (+0.14) and H-3b (+0.06), and a negative value for H-5 (−0.01), indicating an 4*R* configuration in this compound. As a result, the structure of ***anti*-3** was established as *anti*-(1*R*,4*R*)-(–)-thiochroman-4-ol 1-oxide. Despite the low diastereoselectivity observed in the biosynthesis of this product from *P. lilacinum*, its enantioselectivity was moderately high (82.0% *ee*).

The major enantiomer of *syn*-**3a** was subjected to double derivatization using (*R*)-MPA and (*S*)-MPA. A comparison of the ^1^H NMR spectroscopic data of the two MPA esters (Figures S19 and S22) revealed a positive Δ*δ^RS^* for H-5 (+0.73) and negative values for H-3a (−0.36), H-3b (−0.25), H-2a (−0.15), and H-2b (−0.15). These data indicated a 4*S* configuration in ***syn***-**3a**, leading to its assignment as *syn*-(1*R*,4*S*)-(–)-6-chlorothiochroman-4-ol 1-oxide. The absolute configuration of the major enantiomer of *anti*-**3a** was assigned as *anti*-(1*R*,4*R*)-(–)-6-chlorothiochroman-4-ol 1-oxide by comparison of its spectroscopic data and optical activity with those published in the literature [15].

**Figure 3:**
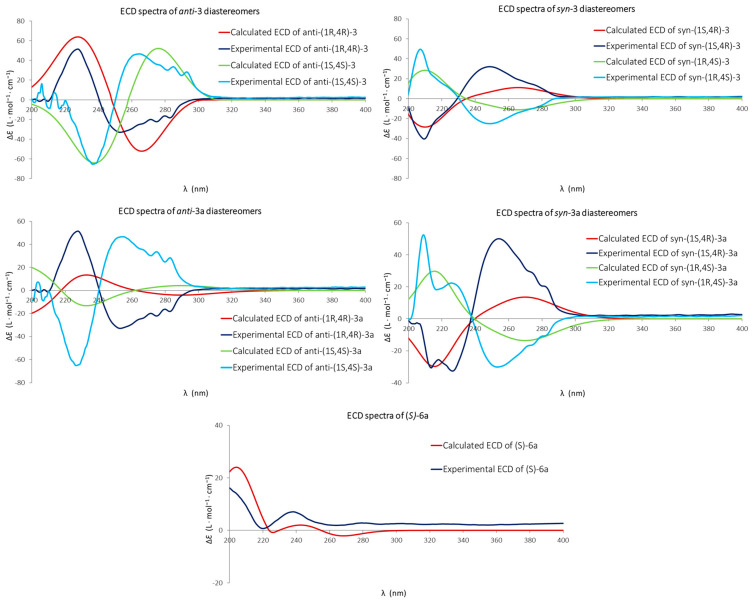
Experimental and calculated ECD spectra for enantiomers of compounds **3**, **3a**, and **6a**.

The absolute configuration of sulfoxides **3** and **3a** was confirmed through a comparison of calculated ECD spectra with the experimental one of each enantiomer (Figure 3). The comparisons were established according to the assignments predicted previously by Mosher’s method.

In accordance with the experimental ECD spectra displayed in Figure 3, ***anti*-(1*R*,4*R*)-3, *syn*-(1*R*,4*S*)-3, *anti*-(1*R*,4*R*)-3a***,* and ***syn*-(1*R*,4*S*)-3a** enantiomers showed positive and negative Cotton effects that were consistent with the ECD curves calculated with the TD-DFT theoretical method, confirming the assignment of their absolute configuration. Despite the low diastereoselectivity observed in the biosynthesis of **3a** by *P. lilacinum* (17.4% *de* in favor of *syn* configuration), the enantiomers ***syn*-(1*R*,4*S*)-3a** and ***anti*-(1*R*,4*R*)-3a** were obtained with a moderately high enantioselectivity, 75.2 and 78.5% *ee*, respectively.

Sulfone **4** was identified as (*R*)-(–)-thiochroman-4-ol 1,1-dioxide by comparing its spectroscopic data and optical activity with literature data for the *S*-configuration product [32,34]. The specific rotation of compound **4** was found to be opposite in sign to that previously published, indicating an *R*-configuration at C-4. The absolute configuration of the major enantiomer of the sulfone **4a** was determined by double derivatization with (*R*)-MPA and (*S*)-MPA, employing Mosher’s method. A comparison of the ^1^H NMR spectroscopic data of the two MPA esters (Figures S25 and S27) revealed a positive Δ*δ^RS^* for H-5 (+0.60) and negative values for H-3a (−0.17), H-3b (−0.34), H-2a (−0.32), and H-2b (−0.24), indicating an 4*S* configuration for this compound. Consequently, the structure of **4a** was established as (*S*)-(+)-6-chlorothiochroman-4-ol 1,1-dioxide. Sulfone (–)-**4a** was initially obtained by Pinedo-Rivilla et al., who assigned a relative configuration *S* at C-4 [15]. The results obtained here prove that the sulfone previously obtained by Pinedo-Rivilla et al. was in fact (*R*)-(–)-6-chlorothiochroman-4-ol 1,1-dioxide [15].

In addition to the bio-oxidation of sulfides, *P. lilacinum* was found to oxidize alcohols to ketones, thereby producing the previously reported sulfoxides **5** [33] and **5a** [35] with moderate enantioselectivity (48.8% and 62.8% *ee*, respectively). Both compounds exhibited specific rotations with opposite signs to those previously reported for the *S*-configuration products. Consequently, the absolute configurations of **5** and **5a** were assigned as (*R*)-(–)-thiochroman-4-one 1-oxide and (*R*)-(–)-6-chlorothiochroman-4-one 1-oxide, respectively.

Compound **6a** was obtained as a colorless oil with the molecular formula C_10_H_13_O_2_SCl, as determined by the HR ESI-MS peak at *m/z* 255.0221 [M + Na]^+^ (Figure S6), indicating four degrees of unsaturation. Considering that the thiochroman skeleton has at least five unsaturations (an aromatic ring and a cycle) and that signals corresponding to a methyl group on sulfur (δ_H_ 2.46 and δ_c_ 16.5) appeared in its ^1^H NMR (Figure S1) and ^13^C NMR (Figure S2) spectra, it was possible to deduce that a ring cleavage had occurred. The ^13^C NMR spectrum (Figure S2) displayed nine additional signals, including six aromatic carbons (*δ*_C_ 144.3, 133.4, 131.8, 127.9, 127.6, 126.0), two methylenes, one of which was oxygenated (*δ*_C_ 61.8, 38.9), and one methine group, also oxygenated (*δ*_C_ 70.8). Its ^1^H NMR spectrum (Figure S1) showed two signals at *δ*_H_ 5.32 (dd, H-1) and 3.91 (dd, H-3), corresponding to a hydroxylated methine and a hydroxylated methylene, respectively. The observation of three signals at *δ*_H_ 7.57 (d, H-6′), 7.22 (dd, H-4′), and 7.14 (d, H-3′) indicated the presence of a trisubstituted aromatic ring, and HMBC correlations from H-1 to C-2/C-3/C-1′/C-2′/C-6′ and from H-3 to C-1/C-2 (Figure S5) allowed the assignment of a propane chain. These data permitted the elucidation of compound **6a** as 1-(5-chloro-2-(methylthio)phenyl)propane-1,3-diol (Figures S1–S6).

The absolute configuration of compound **6a** was determined from the Cotton effects observed in its ECD spectrum (Figure 3). The positive Cotton effect at 200 nm and 238 nm observed in the experimental spectrum corresponded well with those observed in the spectrum predicted from TD-DFT calculations (Figure 3). Accordingly, the structure of compound **6a** was assigned as (*S*)-(+)-1-(5-chloro-2-(methylthio)phenyl)propane-1,3-diol, which was described here for the first time.

Compound **6a** was probably formed by hydroxylation at position 2 of the substrate, followed by ring cleavage, reduction of the aldehyde, and finally methylation at the thiol group.

It is noteworthy that *P. lilacinum* BC17-2 exhibited generally a good enantioselectivity producing all the sulfoxides and a moderate diastereoselectivity in favor of *syn* products in both biotransformations.

### 2.2. Biotransformation of (±)-Thiochroman-4-Ol (**1**) and (±)-6-Chlorothiochroman-4-ol (**1a**) by E. maritima BC17

**Figure 4:**
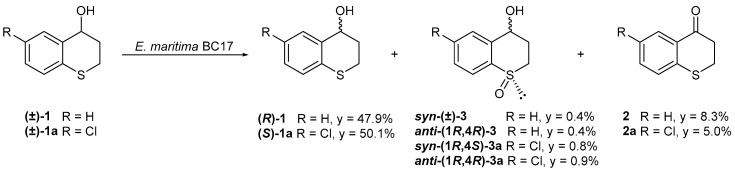
Biotransformation of thiochroman-4-ol (**1**) and 6-chlorothiochroman-4-ol (**1a**) by *E. maritima* BC17. Yields (y) are expressed in % of mol products/mol substrates.

The separate biotransformation of compound **1** and its chlorinated derivative **1a** by *E. maritima* BC17 resulted in the formation of the same three known compounds, some of which differed only in stereochemistry. Specifically, the biotransformation of compound **1** produced *syn*-(±)-thiochroman-4-ol 1-oxide [***syn*-(±)-3**], *anti*-(1*R*,4*R*)-(–)-thiochroman-4-ol 1-oxide [***anti*-(1*R*,4*R*)-3**], and thiochroman-4-one (**2**). In contrast, the biotransformation of compound **1a** yielded *syn*-(1*R*,4*S*)-(–)-6-chlorothiochroman-4-ol 1-oxide [***syn*-(1*R*,4*S*)-3a**], *anti*-(1*R*,4*R*)-(–)-6-chlorothiochroman-4-ol 1-oxide [***anti*-(1*R*,4*R*)-3a**], and 6-chlorothiochroman-4-one (**2a**) (Figure 4).

The starting materials recovered, thiochroman-4-ol (**1**) and 6-chlorothiochroman-4-ol (**1a**), exhibited ${\left[\alpha \right]}_{D\;}^{20}\;$= +17.4 (19.6% *ee*) and ${\left[\alpha \right]}_{D\;}^{20}\;$= −7.9 (18.2% *ee*), in favor of configuration *R* and *S,* respectively, in accordance with the data reported in the literature [28,29]. These data reflect a reversed enantioselectivity of *E. maritima* in comparison to that observed in *P. lilacinum* for the transformation of thiochroman-4-ol (**1**) and 6-chlorothiochroman-4-ol (**1a**).

In contrast to *P. lilacinum* BC17-2, *E. maritima* BC17 exhibited a low enantioselectivity in both biotransformations producing ***syn*-3** (racemic), ***anti*-3** (18.2% *ee*), ***syn*-3a** (37.3% *ee*), and ***anti*-3a** (3.7% *ee*). Interestingly, *E. maritima* BC17 exhibited a preference opposite to that observed in *P. lilacinum* BC17-2 with respect to the **3a**-sulfoxides, showing a moderate diastereoselectivity in favor of the compound ***anti*-3a** (51.0% *de*).

It is noteworthy that *E. maritima* BC17 was able to oxidize the alcohol group from the starting substrates to give the corresponding ketones **2** and **2a**, with excellent yields in both biotransformations. Both fungi performed the oxidation of substrates **1** and **1a**, exhibiting a better yield from the non-chlorinated compound. The sulfoxides and sulfones obtained from the biotransformations were probably formed by oxidation by cytochrome P450 monooxygenases.

### 2.3. Antimicrobial Assays

The antimicrobial activities of the biotransformation products **1**, **1a**, **2**, **2a**, ***syn*-3**, ***anti*-3**, ***syn*-3a**, ***anti*-3a**, **4**, **4a**, **5**, and **5a** were evaluated against two strains from the American Type Culture Collection (*Staphylococcus aureus* ATCC29213 and *Escherichia coli* ATCC25922), as well as the clinical isolate *Candida albicans* HPM-1922816. No significant antimicrobial activity was detected for these compounds against the strains assayed (Figure S42).

## 3. Materials and Methods

### 3.1. General Experimental Methods

Optical rotations were determined using a JASCO P-2000 polarimeter (JASCO Corporation, Tokyo, Japan). Infrared spectra (IR) were recorded on a PerkinElmer Spectrum BX FT-IR (PerkinElmer, Waltham, MA, USA) and reported as wave number (cm^−1^). ^1^H and ^13^C NMR measurements were recorded on Agilent 400 and 500 MHz (Agilent Technologies, Santa Clara, CA, USA), and Bruker 400, 500, and 700 MHz NMR spectrometers (Bruker Corporation, Billerica, MA, USA) with SiMe_4_ as the internal reference. Chemical shifts are expressed in ppm (*δ*) referenced to CDCl_3_ (Eurisotop, Saint-Aubiu, France, *δ*_H_ 7.25, *δ*_C_ 77.0). Two-dimensional NMR experiments were performed using standard Agilent pulse sequence. High-resolution mass spectrometry (HRMS) was performed in a Waters Xevo G2-S QTOF mass spectrometer in the positive-ion ESI mode (Waters Corporation, Milford, MA, USA). TLC and preparative TLC were performed on Merck silica gel 60 Å F254 (Merck Group, Darmstadt, Germany), with a 0.25 and 1 mm layer thickness, respectively. Silica gel 60 (60−200 µm, VWR) was used for column chromatography. Purification by HPLC was performed with a Merck-Hitachi Primaide apparatus equipped with a UV−vis detector (Primaide 1410) and a refractive index detector (RI-5450) (Merck Group, Darmstadt, Germany), and a Merck-Hitachi LaChrom apparatus equipped with a UV−vis detector (L 4250) and a differential refractometer detector (RI-7490) (Merck Group, Darmstadt, Germany). LiChroCART LiChrospher Si 60 (5 µm, 250 mm × 4 mm) (Merck Group, Darmstadt, Germany) and ACE 5 SIL (5 μm, 250 mm × 4.6 mm) (Advanced Chromatography Technologies, Aberdeen, Scotland, UK) column and isocratic *n*-hexane:ethyl acetate and CHCl_3_:CH_3_OH mixtures were used. Diastereoisomeric (*de*) and enantiomeric excesses (*ee*) were determined by means of a Hitachi Chromaster HPLC System equipped with a Diode Array Detector (Hitachi-Chromaster 5430) and a column oven (Hitachi–Chromaster 5310) (Hitachi High-Technologies Corporation, Tokyo, Japan). Chiralcel IB N-5 (, 5 µm, 250 mm × 4.6 mm) (Daicel Corporation, Osaka, Japan) chiral column and isocratic *n*-hexane:*i*-PrOH mixtures were used. Solvents employed were all HPLC grade. Chemicals were provided by Thermo Scientific Chemicals (Thermofisher Scientific, Waltham, MA, USA) and Sigma-Aldrich (Sigma-Aldrich, Darmstadt, Germany).

### 3.2. Fungal Material and Identification

Two marine-derived fungi were used in this study: *E. maritima* BC17 and *P. lilacinum* BC17-2. *E. maritima* BC17 was isolated from intertidal sediments collected in the inner Bay of Cadiz (Cádiz, Spain) [26].

The fungus *P. lilacinum* BC17-2 was also isolated from intertidal sediments collected in the inner Bay of Cadiz (Cádiz, Spain) within a *Spartina* spp. bed with the permission of the national competent authority (ABSCH-CNA-ES-240784-3, reference number ESNC84). Surface sediment samples were collected aseptically in the field, stored in sterile packaging, kept on ice, brought to the laboratory, and immediately processed. Sediment was diluted with sterile seawater water (SSW) and aliquots were grown on PDA plates and marine agar plates (Condalab S.L., Madrid, Spain) and incubated at 25 °C for 5–10 days. Fungal colonies were selected and streaked on PDA plates under axenic conditions. The isolates were maintained on PDA at 25 °C for routine experiments and spores were stored in 60% (*v*/*v*) glycerol at −20 °C for later studies.

The BC17-2 fungal strain isolated was identified as *P. lilacinum* using the service of the Spanish Type Culture Collection (CECT, https://www.uv.es/cect, accessed on 7 December 2023) based on both phenotypic and molecular techniques. Two regions of the fungal genome were amplified by conventional PCR: (a) amplification and sequencing (with readings in both directions) of the ribosomal DNA region comprising the intergenic spaces ITS1 and ITS2, including the 5.8S rRNA (ITS5 5′-GGAAGTAAAAGTCGTAACAAGG-3′; ITS4 (5′-TCCTCCGCTTATTGATATGC-3′); and (b) partial amplification and sequencing (with readings in both directions) of the 28S rRNA gene (LR0R 5′-GTACCCGCTGAACTTAAGC-3′; LR7 5′-TACTACCACCAAGATCT-3′). The sequencing of these regions was compared with those in NCBI databases. Sequences were submitted to the NCBI database with the accession number OR884977 for the ITS region and OR884976 for the 28S rRNA gene. To study the phylogenetic relationship of our isolate, other sequences of related genera and species (107 sequences) from the family *Ophiocordycipitaceae* were downloaded from the GenBank database and included in the phylogenetic trees.

Cultures of *E. maritima* BC17 and *P. lilacinum* BC17-2 have been deposited at the University of Cádiz, Mycological Herbarium Collection (UCA). Conidial stock suspensions of these strains are maintained as viable in 80% glycerol at −40 °C.

### 3.3. Synthesis of Racemic Substrates (**1**, **1a**)

Thiochroman-4-one (**2**) (1.7 g, 0.010 mol, 97%) (Sigma-Aldrich, Darmstadt, Germany) and 6-chlorothiochroman-4-one (**2a**) (1.5 g, 0.008 mol, 98%) (Thermo Fisher Scientific, Waltham, MA, USA) were treated with NaBH_4_ (800 mg, 0.021 mol) in CH_2_Cl_2_:CH_3_OH 1:1 (200 mL) and stirred for 24 h at room temperature. Then, the solvent was evaporated under reduced pressure and the crudes were diluted with water, neutralized with aqueous HCl 10%, and extracted with ethyl acetate. The organic layers were dried over Na_2_SO_4_ and the solvents were evaporated under reduced pressure. The reduction mixtures were chromatographed on a silica gel column, eluting with *n*-hexane-ethyl acetate mixtures, to give thiochroman-4-ol (**1**) (1.6 g, 0.094 mol, 94% yield) and 6-chlorothiochroman-4-ol (**1a**) (1.3 g, 0.007 mol, 85% yield). Spectroscopic data of compounds **1** [28] and **1a** [29] were identical to those described in the literature.

### 3.4. Biotransformation Experiments

#### 3.4.1. General Method

*E. maritima* BC17 and *P. lilacinum* BC17-2 were grown in Roux bottles (1 L), each containing 150 mL of potato dextrose broth (PDB, Condalab S. L., Madrid, Spain). Each bottle was inoculated with 5 mycelium plugs having a 0.9 cm diameter from a seven-day-old culture on potato dextrose agar (PDA, Condalab, Madrid, Spain) or 10^6^ fresh conidia/mL and incubated at 25 °C under white light (day light lamp). After 4 days, a solution of the substrate in ethanol was added to achieve a final concentration of 150 ppm. Roux bottles were incubated under the same conditions described above for 6, 8, or 11 days more (see detailed experiments below). Then, the broth was filtered and extracted with ethyl acetate (×3). The organic extract was dried over anhydrous Na_2_SO_4_ and the solvent was evaporated under reduced pressure. The residue was subjected to column chromatography on silica gel, using a gradient mixture of *n*-hexane/ethyl acetate of increasing polarity. Fractions containing the *syn* and *anti* diastereoisomers of sulfoxides **3** and **3a** were further purified by preparative TLC, using CHCl_3_: CH_3_OH 3:97 (*v*/*v*) as the solvent system. Final purification was carried out by HPLC with LiChroCART LiChrospher Si 60, ACE 5 SIL, or Chiralcel IB N-5 columns and an isocratic *n*-hexane:ethyl acetate, CHCl_3_:CH_3_OH, or *n*-hexane:*i*-PrOH mixtures as mobile phases.

#### 3.4.2. Biotransformation of (±)-Thiochroman-4-Ol (**1**) by *P. lilacinum* BC17-2

In accordance with the above procedure, 30 Roux bottles were subcultured with mycelium plugs of *P. lilacinum* BC17-2. Each bottle was fed with a solution of thiochroman-4-ol (**1**) in ethanol on day 4 to a final concentration of 150 ppm. The substrate control contained sterile medium and the same concentration of **1** dissolved in ethanol. Extraction of the broth 8 days post-feeding yielded a crude extract (425.9 mg), which was purified as described in the general method to afford **(*S*)-1** (28.2 mg), ***syn*-(1*R*,4*S*)-3** (30.4 mg), ***anti*-(1*R*,4*R*)-3** (14.6 mg), **(*R*)-4** (12.3 mg), and **(*R*)-5** (4.2 mg).

(*S*)-(–)-Thiochroman-4-ol [**(*S*)-1**]. Colorless oil. ${\left[\alpha \right]}_{D\;}^{20}\;$= −4.1 (c 2.1, CHCl_3_), 1.6% *ee*. HPLC conditions: Chiralcel IB N-5, Daicel, Japan, *n*-hexane:*i*PrOH 95:5, flow rate 0.6 mL/min, t_R_ = 22.19 min (*S*) and 26.75 min (*R*) (Figure S29).

*syn*-(1*R*,4*S*)-(–)-Thiochroman-4-ol 1-oxide [***syn*-(1*R*,4*S*)-3**]. Colorless oil. ${\left[\alpha \right]}_{D\;}^{26}\;$= −33.1 (c 0.4, MeOH), 52.7% *ee*, 31.5% *de* (Figure S40). HPLC conditions: Chiralcel IB N-5, Daicel, Japan, *n*-hexane:*i*PrOH 93:7, flow rate 0.6 mL/min, t_R_ 50.6 min (1*R*,4*S*) and 60.1 min (1*S*,4*R*) (Figure S30). ^1^H NMR (CDCl_3_, 400 MHz) *δ* 7.73 (dd, *J* = 7.5, 1.4 Hz, 1H, H-8), 7.63 (m, 1H, H-5), 7.55 (td, *J* = 7.5, 1.4 Hz, 1H, H-6), 7.45 (dtd, *J* = 7.5, 1.5 Hz, 1H, H-7), 4.81 (t, *J* = 5.7 Hz, 1H, H-4), 3.39 (ddd, *J* = 13.7, 6.8, 4.9 Hz, 1H, H-2a), 2.99 (m, 1H, H-2b), 2.63 (tddd, *J* = 14.7, 8.9, 6.3, 4.9 Hz, 1H, H-3a), 2.30 (dddt, *J* = 14.7, 9.4, 6.8, 5.2 Hz, 1H, H-3b). ^13^C NMR (CDCl_3_, 400 MHz) *δ* 139.2 (C, C-10), 138.8 (C, C-9), 132.6 (CH, C-6), 129.4 (CH, C-8), 129.2 (CH, C-5), 128.9 (CH, C-7), 67.1 (CH, C-4), 44.1 (CH_2_, C-2), 24.9 (CH_2_, C-3).

*anti*-(1*R*,4*R*)-(–)-Thiochroman-4-ol 1-oxide [***anti*-(1*R*,4*R*)-3**]. Colorless oil. ${\left[\alpha \right]}_{D\;}^{25}\;$= −38.9 (c 0.4, MeOH), 82.0% *ee*. HPLC conditions: Chiralcel IB N-5, Daicel, Japan, *n*-hexane:*i*PrOH 93:7, flow rate 0.6 mL/min, t_R_ 67.6 min (1*R*,4*R*) and 71.8 min (1*S*,4*S*) (Figure S31). ^1^H NMR (CDCl_3_, 400 MHz) *δ* 7.76 (dd, *J* = 7.9, 1.6 Hz, 1H, H-8), 7.57–7.45 (m, 3H, H-5, H-6, H-7), 5.00 (t, *J* = 4.2 Hz, 1H, H-4), 3.43 (ddd, *J* = 13.9, 11.2, 3.4 Hz, 1H, H-2a), 3.07 (ddd, *J* = 13.9, 6.3, 3.4 Hz, 1H, H-2b), 2.81 (ddt, *J* = 14.8, 11.2, 3.4 Hz, 1H, H-3a), 2.19 (dddd, *J* = 14.8, 6.3, 4.2, 3.4 Hz, 1H, H-3b). ^13^C NMR (CDCl_3_, 400 MHz) *δ* 138.0 (C, C-9), 136.6 (C, C-10), 132.3 (CH, C-6), 130.7 (CH, C-5), 130.1 (CH, C-8), 129.6 (CH, C-7), 65.9 (CH, C-4), 42.0 (CH_2_, C-2), 22.7 (CH_2_, C-3).

(*R*)-(–)-Thiochroman-4-ol 1,1-dioxide [**(*R*)-4**]. Colorless oil. ${\left[\alpha \right]}_{D\;}^{27}\;$= −5.9 (c 0.3, CHCl_3_), 23.1% *ee*. HPLC conditions: Chiralcel IB N-5, Daicel, Japan, *n*-hexane:*i*PrOH 93:7, flow rate 0.6 mL/min, t_R_ 90.9 min (*R*) and 95.0 min (*S*) (Figure S32).

(*R*)-(–)-Thiochroman-4-one 1-oxide [**(*R*)-5**]. Colorless oil. ${\left[\alpha \right]}_{D\;}^{24}\;$= −39.1 (c 0.4, MeOH), 48.8% *ee*. HPLC conditions: Chiralcel IB N-5, Daicel, Japan, *n*-hexane:*i*PrOH 70:30, flow rate 0.8 mL/min, t_R_ 18.4 min (*S*) and 19.4 min (*R*) (Figure S33).

#### 3.4.3. Biotransformation of (±)-6-Chlorothiochroman-4-Ol (**1a**) by *P. lilacinum* BC17-2

Thirty subcultured Roux bottles with mycelium plugs of *P. lilacinum* BC17-2 were fed with a solution of 6-chlorothiochroman-4-ol (**1a**) in ethanol on day 4 to a final concentration of 150 ppm. Negative control consisted of the sterile medium with **1a** at the same concentration. Extraction of the broth 8 days post-feeding yielded a crude extract (437.7 mg), which was purified as described in the general method to afford **(*R*)-1a** (36.5 mg), ***syn*-(1*R*,4*S*)-3a** (54.1 mg), ***anti*-(1*R*,4*R*)-3a** (41.7 mg), **(*S*)-4a** (25.0 mg), **(*R*)-5a** (1.7 mg), and **(*S*)-6a** (1.5 mg).

(*R*)-(+)-6-Chlorothiochroman-4-ol [**(*R*)-1a**]. Colorless oil. ${\left[\alpha \right]}_{D\;}^{21}\;$= +22.5 (c 2.4, CHCl_3_), 28.5% *ee*. HPLC conditions: Chiralcel IB N-5, Daicel, Japan, *n*-hexane:*i*PrOH 95:5, flow rate 0.6 mL/min: t_R_ 19.4 min (*S*) and 22.7 min (*R*) (Figure S34).

*syn*-(1*R*,4*S*)-(–)-6-Chlorothiochroman-4-ol 1-oxide [***syn*-(1*R*,4*S*)-3a**]. Colorless oil. ${\left[\alpha \right]}_{D\;}^{24}\;$= −70.9 (c 0.2, MeOH), 75.2% *ee*, 17.4% *de* (Figure S41). HPLC conditions: Chiralcel IB N-5, Daicel, Japan, *n*-hexane:*i*PrOH 93:7, flow rate 0.6 mL/min, t_R_ 41.7 min (1*R,*4*S*) and 49.4 min (1*S*,4*R*) (Figure S35).

*anti*-(1*R*,4*R*)-(–)-6-Chlorothiochroman-4-ol 1-oxide [***anti*-(1*R*,4*R*)-3a**]. Colorless oil. ${\left[\alpha \right]}_{D\;}^{25}\;$= −82.7 (c 1.10, MeOH), 78.5% *ee*. HPLC conditions: Chiralcel IB N-5, Daicel, Japan, *n*-hexane:*i*PrOH 93:7, flow rate 0.6 mL/min, t_R_ 54.0 min (1*R*,4*R*) and 60.1 min (1*S*,4*S*) (Figure S36).

(*S*)-(+)-6-Chlorothiochroman-4-ol 1,1-dioxide [**(*S*)-4a**]. Colorless oil. ${\left[\alpha \right]}_{D\;}^{27}\;$= +10.8 (c 0.3, CHCl_3_), 44.6% *ee*. HPLC conditions: Chiralcel IB N-5, Daicel, Japan, *n*-hexane:*i*PrOH 93:7, flow rate 0.6 mL/min, t_R_ 77.7 min (*R*) and 81.3 min (*S*) (Figure S37).

(*R*)-(–)-6-Chlorothiochroman-4-one 1-oxide [**(*R*)-5a**]. Colorless oil. ${\left[\alpha \right]}_{D\;}^{25}\;$= −98.0 (c 0.2, MeOH), 62.8% *ee*. HPLC conditions: Chiralcel IB N-5, Daicel, Japan, *n*-hexane:*i*PrOH 93:7, flow rate 0.6 mL/min, t_R_ 117.1 min (*R*) and 133.8 min (*S*) (Figure S38).

(*S*)-(+)-1-(5-Chloro-2-(methylthio)phenyl)propane-1,3-diol [**(*S*)-6a**]. Colorless oil. ${\left[\alpha \right]}_{D\;}^{20}\;$= +10.6 (c 0.1, CHCl_3_), 52.1% *ee*. HPLC conditions: Chiralcel IB N-5, Daicel, Japan, *n*-hexane:*i*PrOH 98:2, flow rate 0.6 mL/min, t_R_ 77.36 min (major) and 80.37 min (minor) (Figure S39). *υ*_max_ (cm^−1^) 3361, 2917, 1459, 1438, 1105, 1045, 806. ^1^H NMR (CDCl_3_, 700 MHz) *δ* 7.57 (d, 1H, *J* = 2.4 Hz, H-6′), 7.22 (dd, 1H, *J* = 8.4, 2.4 Hz, H-4′), 7.14 (d, 1H, *J* = 8.4 Hz, H-3′), 5.32 (dd, 1H, *J* = 8.9, 3.1 Hz, H-1), 3.91 (dd, 2H, *J* = 6.9, 4.2 Hz, H-3), 2.46 (s, 3H, S-Me), 1.99 (m, 1H, H-2a), 1.91 (m, 1H, H-2b). ^13^C NMR (CDCl_3_, 175 MHz) *δ* 144.3 (C, C-1′), 133.4 (C, C-2′), 131.8 (C, C-5′), 127.9 (CH, C-4′), 127.6 (CH, C-3′), 126.0 (CH, C-6′), 70.8 (CH, C-1), 61.8 (CH_2_, C-3), 38.9 (CH_2_, C-2), 16.5 (CH_3_, S-Me). HR ESI-MS *m/z* 255.0221 [M + Na]^+^ (calcd. for C_10_H_13_O_2_NaSCl, 255.0222) (Figures S1–S6).

#### 3.4.4. Biotransformation of (±)-Thiochroman-4-Ol (**1**) by *E. maritima* BC17

Twenty Roux bottles were subcultured with 10^6^ fresh conidia/mL of *E maritima* BC17. Each bottle was fed with a solution of thiochroman-4-ol (**1**) in ethanol on day 4 to a final concentration of 150 ppm. Extraction of the broth 6 days post-feeding yielded a crude extract (466.3 mg), which was purified as described in the general method to afford (*R*)-(+)-thiochroman-4-ol [**(*R*)-1**] (323.1 mg) {${\left[\alpha \right]}_{D\;}^{20}\;$= +17.4 (c 4.8, MeOH), 19.6% *ee*} (Figure S29), *syn*-(±)-thiochroman-4-ol 1-oxide [***syn*-(±)-3**] (3.3 mg) (racemic, 7.0% *de*) (Figures S30 and S40), and *anti*-(1*R*,4*R*)-(-)-thiochroman-4-ol 1-oxide [***anti*-(1*R*,4*R*)-3**] (2.9 mg) {18.2 % *ee*} (Figure S31), and thiochroman-4-one (**2**) (55.1 mg). Negative control consisted of the sterile medium with **1** at the same concentration.

#### 3.4.5. Biotransformation of (±)-6-Chlorothiochroman-4-Ol (**1a**) by *E. maritima* BC17

Twenty Roux bottles were subcultured with 10^6^ fresh conidia/mL of *E maritima* BC17. Each bottle was fed with a solution of 6-chlorothiochroman-4-ol (**2**) in ethanol on day 4 to a final concentration of 150 ppm. Extraction of the broth 11 days post-feeding yielded a crude extract (493.4 mg), which was purified as described in the general method to afford (*S*)-(+)-6-chlorothiochroman-4-ol [**(*S*)-1a**] (338.2 mg) {${\left[\alpha \right]}_{D\;}^{20}\;$= −7.9 (c 0.3, MeOH), 18.2% *ee*} (Figure S34), *syn*-(1*R*,4*S*)-(-)-6-chlorothiochroman-4-ol 1-oxide [***syn*-(1*R*,4*S*)-3a**] (6.0 mg) {${\left[\alpha \right]}_{D\;}^{20}\;$= −19.4 (c 0.42, MeOH), 37.3% *ee*} (Figure S35), *anti*-(1*R*,4*R*)-(-)-6-chlorothiochroman-4-ol 1-oxide [***anti*-(1*R*,4*R*)-3a**] (6.2 mg) {${\left[\alpha \right]}_{D\;}^{20}\;$= −10.5 (c 0.40, MeOH), 3.7% *ee*, 51.0% *de*} (Figures S36 and S41), and 6-chlorothiochroman-4-one (**2a**) (27.6 mg). Negative control consisted of the sterile medium with **1a** at the same concentration.

### 3.5. General Procedure for the Preparation of Mosher’s Esters

*N,N*-Dimethylaminopyridine (DMPA, 4.2 mg, 0.033 mmol, 98%) (Sigma-Aldrich, Darmstadt, Germany), 1-ethyl-3-(3-dimethylaminopropyl) carbodiimide (EDC, 6.0 mg, 0.026 mmol, 98%) (Tokyo Chemical Industry Co., Ltd., Tokyo, Japan), and (*R*)- or (*S*)-methoxyphenylacetic acid (MPA, 5.0 mg, 0.003 mmol, 99%) (Sigma-Aldrich, Darmstadt, Germany) were added to a stirred solution of the corresponding thiochroman-4-ols ***syn*-3**, ***anti*-3**, ***syn*-3a**, and **4a** (0.018 mmol) in dry CH_2_Cl_2_ (1 mL). The resulting mixtures were stirred at room temperature for 3–4 h. Then, the solvents were evaporated under reduced pressure and the residues were subjected to column chromatography on silica gel to afford (*R*)-*α*-methoxyphenylacetyl esters of ***syn*-3** (92% yield), ***syn*-3a** (59%), and **4a** (97%); and (*S*)-*α*-methoxyphenylacetyl esters of ***syn*-3** (58%), ***anti*-3** (89%), ***syn*-3a** (60%), and **4a** (92%).

(*R*)-*α*-methoxyphenylacetyl ester of ***syn*-3**. ^1^H NMR (500 MHz, CDCl_3_) *δ* 7.82 (dd, *J* = 7.7, 1.6 Hz, 1H, H-8), 7.52 (ddd, *J* = 7.7, 7.6, 1.5 Hz, 1H, H-7), 7.48 (ddd, *J* = 7.6, 7.6, 1.6 Hz, 1H, H-6), 7.44 – 7.40 (m, 2H, Ph-2′), 7.39 – 7.32 (m, 3H, Ph-2′), 7.33 (dd, *J* = 7.6, 1.5 Hz, 1H, H-5), 6.02 (dd, *J* = 6.5, 4.4 Hz, 1H, H-4), 4.82 (s, 1H, H-2′), 3.42 (s, 3H, OMe-2′), 3.07 (ddd, *J* = 12.9, 8.5, 2.8 Hz, 1H, H-2a), 3.01 (ddd, *J* = 12.9, 10.1, 2.8 Hz, 1H, H-2b), 2.27 (dddd, *J* = 15.1, 8.5, 6.5, 2.8 Hz, 1H, H-3a), 2.12 (dddd, *J* = 15.1, 10.1, 4.4, 2.8 Hz, 1H, H-3b) (Figures S7–S9).

(*S*)-*α*-methoxyphenylacetyl ester of ***syn*-3**. ^1^H NMR (500 MHz, CDCl_3_) *δ* 7.74 (dd, *J* = 7.9, 1.4 Hz, 1H, H-8), 7.45 – 7.38 (m, 3H, H-7, Ph-2′), 7.38 – 7.34 (m, 3H, Ph-2′), 7.21 (ddd, *J* = 7.7, 7.7, 1.4 Hz, 1H, H-6), 6.63 (dt, *J* = 7.7, 1.1 Hz, 1H, H-5), 5.95 (dd, *J* = 7.8, 5.0 Hz, 1H, H-4), 4.82 (s, 1H, H-2′), 3.43 (s, 3H, OMe-2′), 3.23 (ddd, *J* = 13.2, 8.9, 2.9 Hz, 1H, H-2a), 3.18 (m, 1H, H-2b), 2.65 (dddd, *J* = 14.8, 9.8, 7.8, 2.9 Hz, 1H, H-3a), 2.36 (dddd, *J* = 14.8, 8.9, 5.0, 2.8 Hz, 1H, H-3b) (Figures S10–S12).

(*S*)-*α*-methoxyphenylacetyl ester of ***anti*-3**. ^1^H NMR (500 MHz, CDCl_3_, T_1_ = 25 °C) *δ* 7.65 (m, 1H, H-8), 7.44 – 7.38 (m, 2H, H-7, H-6), 7.29 (dd, *J* = 1.7, 0.7 Hz, 1H, H-5), 7.32 – 7.23 (m, 5H, Ph-2′), 6.02 (t, *J* = 3.5 Hz, 1H, H-4), 4.60 (s, 1H, H-2′), 3.26 (s, 3H, OMe-2′), 2.77 (m, 2H, H-2a, H-2b), 2.66 (m, 1H, H-3a), 1.84 (m, 1H, H-3b). ^1^H NMR (500 MHz, CDCl_3_, T_2_ = -25 °C) *δ* 7.63 (m, 1H, H-8), 7.43 (m, 2H, H-6, H-7), 7.30 (q, *J* = 3.6 Hz, 1H, H-5), 7.31 – 7.23 (m, 5H, Ph-2′), 5.99 (d, *J* = 3.4 Hz, 1H, H-4), 4.59 (s, 1H, H-2′), 3.23 (s, 3H, OMe-2′), 2.76 (dd, *J* = 12.4, 4.8 Hz, 1H, H-2a), 2.65 (m, 1H, H-3a), 2.63 (m, 1H, H-2b), 1.78 (dt, *J* = 12.3, 3.8 Hz, 1H, H-3b) (Figures S13–S18).

(*R*)-*α*-methoxyphenylacetyl ester of ***syn*-3a**. ^1^H NMR (400 MHz, CDCl_3_) *δ* 7.74 (d, *J* = 8.4 Hz, 1H, H-8), 7.48 (dd, *J* = 8.4, 2.2 Hz, 1H, H-7), 7.46 – 7.34 (m, 5H, Ph-2′), 7.29 (dd, *J* = 2.2, 0.8 Hz, 1H, H-5), 5.94 (dd, *J* = 6.9, 4.6 Hz, 1H, H-4), 4.85 (s, 1H, H-2′), 3.43 (s, 3H, OMe-2′), 3.03 (m, 2H, H-2a, H-2b), 2.28 (dddd, *J* = 15.4, 8.6, 6.9, 3.0 Hz, 1H, H-3a), 2.12 (m, 1H, H-3b) (Figures S19–S21).

(*S*)-*α*-methoxyphenylacetyl ester of ***syn*-3a**. ^1^H NMR (400 MHz, CDCl_3_) *δ* 7.66 (d, *J* = 8.4 Hz, 1H, H-8), 7.47 – 7.39 (m, 4H, Ph-2′), 7.37 (dd, *J* = 8.5, 1.7 Hz, 1H, H-7), 7.33 (s, 1H, Ph-2′), 6.56 (dd, *J* = 2.2, 0.9 Hz, 1H, H-5), 5.87 (dd, *J* = 8.3, 5.1 Hz, 1H, H-4), 4.84 (s, 1H, H-2′), 3.44 (s, 3H, OMe-2′), 3.18 (m, 2H, H-2a, H-2b), 2.64 (m, 2H, H-3a), 2.37 (dddd, *J* = 13.2, 8.1, 5.2, 3.0 Hz, 1H, H-3b) (Figures S22–S24).

(*R*)-*α*-methoxyphenylacetyl ester of **4a**. ^1^H NMR (400 MHz, CDCl_3_) *δ* 7.86 (d, *J* = 8.5 Hz, 1H, H-8), 7.53 (dd, *J* = 8.5, 2.1 Hz, 1H, H-7), 7.38 (dq, *J* = 3.5, 1.6 Hz, 5H, Ph-2′), 7.34 (d, *J* = 2.1 Hz, 1H, H-5), 6.04 (t, *J* = 4.6 Hz, 1H, H-4), 4.79 (s, 1H, H-2′), 3.40 (s, 3H, OMe-2′), 3.20 (ddd, *J* = 14.4, 12.1, 2.9 Hz, 1H, H-2a), 3.09 (ddd, *J* = 14.4, 6.6, 3.1 Hz, 1H, H-2b), 2.65 (ddt, *J* = 15.4, 12.1, 3.1 Hz, 1H, H-3a), 2.26 (dddd, *J* = 15.4, 6.6, 4.6, 2.9 Hz, 1H, H-3b) (Figures S25–S26).

(*S*)-*α*-methoxyphenylacetyl ester of **4a**. ^1^H NMR (400 MHz, CDCl_3_) *δ* 7.80 (d, *J* = 8.5 Hz, 1H, H-8), 7.43 (ddd, *J* = 8.5, 2.1, 0.5 Hz, 1H, H-7), 7.39 – 7.33 (m, 5H, Ph-2′), 6.74 (dt, *J* = 2.1, 0.5 Hz, 1H, H-5), 5.98 (dd, *J* = 6.2, 4.1 Hz, 1H, H-4), 3.52 (m, 1H, H-2a), 3.33 (m, 1H, H-2b), 2.82 (dddd, *J* = 15.1, 10.7, 4.1, 2.9 Hz, 1H, H-3a), 2.60 (dddd, *J* = 15.1, 8.2, 6.2, 2.9 Hz, 1H, H-3b) (Figures S27 and S28).

### 3.6. Computational Details of ECD Calculations

Quantum mechanical computations were executed utilizing the Gaussian 09 package [36]. The geometric optimization of compounds ***syn*-3**, ***anti*-3**, ***syn*-3a**, ***anti*-3a**, and **6a** was conducted through the application of the density functional theory (DFT) within the framework of B3LYP functionals and the 6−311+G(2d,p) basis set [37,38]. Subsequently, calculations were performed to determine the energies, oscillator strengths, and rotational strengths associated with the initial 20 electronic excitations, employing the TD-DFT methodology [39,40]. The solvent’s influence (methanol) was considered within the calculations, incorporating the polarizable continuum model (PCM) with the implementation of the implicit solvation energy (IEF) approach [41,42,43]. To mimic the ECD spectrum of the conformer a Gaussian function was used featuring a half-bandwidth of 0.33 eV.

### 3.7. In Vitro Antimicrobial Assays

The antimicrobial activities of biotransformation compounds **1**, **1a**, **2**, **2a**, ***syn*-3**, ***anti*-3**, ***syn*-3a**, ***anti*-3a**, **4**, **4a**, **5**, and **5a** were evaluated against bacterial and fungal human pathogens. Antibacterial susceptibility of the compounds was tested against the strains *S. aureus* ATCC29213 and *E. coli* ATCC25922 from the American Type Culture Collection. Antifungal activity was tested against the clinical isolate *C. albicans* HPM-1922816.

Assays were conducted on 96-well culture plates using a microdilution method according to Clinical and Laboratory Standards Institute (CLSI) procedures for bacteria, and to European Committee on Antimicrobial Susceptibility Testing for yeasts [44,45,46]. The compounds were tested with 1:2 dilutions starting at 256 µg/mL in independent triplicates to validate results.

For the antibacterial assays, Mueller–Hinton Broth (Sigma-Aldrich, Darmstadt, Germany) was used as the growth medium. The strains were first streaked onto blood agar plates and incubated overnight at 37 °C. Isolated colonies were inoculated in saline serum in order to reach a 0.5 McFarland standard turbidity, and then diluted 1:100 in Mueller–Hinton Broth to obtain an assay inoculum of 1 × 10^6^ colony-forming units (cfu)/mL. Finally, bacteria were inoculated in well plates reaching an assay concentration of approximately 5 × 10^5^ cfu/mL.

For the antiyeast assay, RPMI-1640 (with l-glutamine, *w*/*o* Na_2_CO_3_) (Biowest, Nuaillé, France) with phenol red as a pH indicator was used as the growth medium. The stock inoculum was first streaked onto a Sabouraud Dextrose agar plate (Sigma-Aldrich, Darmstadt, Germany) and incubated at 37 °C overnight. Single colonies were then resuspended in sterile distilled water, resulting in a 0.5 McFarland standard turbidity. The suspension was then diluted 1:10 in RPMI-1640 medium, reaching an assay inoculum concentration of approximately 5 × 10^5^ cfu/mL. Finally, it was inoculated to achieve an assay concentration of 2.5 × 10^5^ cfu/mL.

Viability counts were performed in order to ensure the inoculum concentration. Proper blanks were assayed simultaneously. Fosfomycin and amphotericin B (Sigma-Aldrich, Darmstadt, Germany) were used as reference against bacteria and yeast*,* respectively. The MIC was determined as the lowest concentration that showed no visible growth after 18–20 h of incubation at 37 °C.

## 4. Conclusions

Biotransformation of thiochroman-4-ol (**1**) and 6-chlorothiochroman-4-ol (**1a**) using the marine-derived fungal strains *E. maritima* BC17 and *P. lilacinum* BC17-2 yielded ten known thiochroman derivatives and the new compound (*S*)-1-(5-chloro-2-(methylthio)phenyl)propane-1,3-diol (**6a**).

The study revealed that the marine fungal strains employed exhibited a notable capacity for biotransforming thiochroman derivatives, indicating that these fungi possess an enormous potential as biocatalysts for the modification of such structures, particularly oxidations. This is of particular interest for obtaining enantiomerically enriched sulfoxides with interesting biological activities, whose scaffold is considered a suitable building block in drug design and organic synthesis [9,10]. Notably, the strain *P. lilacinum* BC17-2, isolated and identified for the first time in the Bay of Cadiz (Spain) in the present study, was able to open the tetrahydro thiopyran ring of the thiochroman substrate, resulting in the formation of the new compound (*S*)-1-(5-chloro-2-(methylthio)phenyl)propane-1,3-diol (**6a**). Specifically, this fungal strain showed a higher ability to biotransform these substrates than *E. maritima* BC17.

The results demonstrate a reversal enantioselectivity for *E. maritima* in comparison to that observed in *P. lilacinum* for the transformation of thiochroman-4-ol (**1**) and 6-chlorothiochroman-4-ol (**1a**). It is noteworthy that *E. maritima* BC17 exhibited a preference contrary to that observed in *P. lilacinum* BC17-2 with respect to the **3a**-sulfoxides, exhibiting a moderate diastereoselectivity in favor of the compound ***anti*-3a**. In contrast, *P. lilacinum* exhibited a preference for the stereoisomer *syn*.

Antimicrobial activity of the isolated compounds **1**, **1a**, **2**, **2a**, ***syn*-3**, ***anti*-3**, ***syn*-3a**, ***anti*-3a**, **4**, **4a**, **5**, and **5a** was evaluated against human pathogenic bacteria and yeast but unfortunately no significant activity was detected.

## Data Availability

Data are contained within the article and Supplementary Materials.

## Supplementary Materials

The following supporting information can be downloaded at: https://www.mdpi.com/article/10.3390/ijms26030908/s1.
