# Supplementary material for: Biotransformation of Thiochroman Derivatives Using Marine-Derived Fungi: Isolation, Characterization, and Antimicrobial Activity
Source: Int J Mol Sci. 2025 Jan 22;26(3):908. doi: 10.3390/ijms26030908 (PMC11816680; doi:10.3390/ijms26030908)
Supplement: Supplementary file 1 [file ijms-26-00908-s001.zip › ijms-3356471-supplementary.pdf]

Biotransformation of Thiochroman Derivatives Using Marine-Derived Fungi: Isolation, Characterization, and Antimicrobial Activity

Jorge R. Virués-Segovia,[1,2] Cristina Pinedo-Rivilla,[1,2] Salvador Muñoz-Mira,[1] Matilde Ansino,[1] Victoria E. González-Rodríguez,[3] Abdellah Ezzanad,[1] Fátima Galán-Sánchez,[4,5] Rosa Durán-Patrón[1,2,*], Josefina Aleu,[1,2,*]

[1] Departamento de Química Orgánica, Facultad de Ciencias, Universidad de Cádiz, Puerto Real, 11510 Cádiz, Spain.

[2] Instituto de Investigación en Biomoléculas (INBIO), Universidad de Cádiz, Puerto Real, 11510 Cádiz, Spain.

[3] Laboratorio de Microbiología, Departamento de Biomedicina, Biotecnología y Salud Pública, Facultad de Ciencias del Mar y Ambientales, Universidad de Cádiz, 11510 Puerto Real, Cádiz, Spain.

[4] Servicio de Microbiología. Hospital Universitario Puerta del Mar, 11009 Cádiz, Spain.

[5] Instituto de investigación e Innovación Biomédica de Cádiz (INIBICA), 11009 Cádiz, Spain.

*Correspondence: josefina.aleu@uca.es (J.A.), rosa.duran@uca.es (R.D.-P.)

Supporting Information Content

Figure S1. ^1H NMR spectrum (700 MHz, $CDCl_3$) of compound **6a**. 4

Figure S2. ^{13}C NMR spectrum (175 MHz, $CDCl_3$) of compound **6a**. 5

Figure S3. gCOSY spectrum of compound **6a**. 6

Figure S4. gHSQC spectrum of compound **6a**. 7

Figure S5. gHMBC spectrum of compound **6a**. 8

Figure S6. HR ESI-MS spectrum of compound **6a**. 9

Figure S7. ^1H NMR spectrum (500 MHz, $CDCl_3$) of compound **(*R*)-MPA-*syn*-(1*R*, 4*S*)-3.** 10

Figure S8. ^{13}C NMR spectrum (125 MHz, $CDCl_3$) of compound **(*R*)-MPA-*syn*-(1*R*, 4*S*)-3**. 11

Figure S9. gCOSY spectrum of compound **(*R*)-MPA-*syn*-(1*R*, 4*S*)-3**. 12

Figure S10. ^1H NMR spectrum (500 MHz, $CDCl_3$) of compound **(*S*)-MPA-*syn*-(1*R*, 4*S*)-3**. 13

Figure S11. ^{13}C NMR spectrum (125 MHz, $CDCl_3$) of compound **(*S*)-MPA-*syn*-(1*R*, 4*S*)-3**. 14

Figure S12. gCOSY spectrum of compound **(*S*)-MPA-*syn*-(1*R*, 4*S*)-3**. 15

Figure S13. ^1H NMR spectrum (500 MHz, $CDCl_3$) of compound **(*S*)-MPA-*anti*-(1*R*, 4*R*)-3 (T = 25ºC)**. 16

Figure S14. ^{13}C NMR spectrum (125 MHz, $CDCl_3$) of compound **(*S*)-MPA-*anti*-(1*R*, 4*R*)-3 (T = 25ºC)**. 17

Figure S15. gCOSY spectrum of compound **(*S*)-MPA-*anti*-(1*R*, 4*R*)-3 (T = 25ºC)**. 18

Figure S16. ^1H NMR spectrum (500 MHz, $CDCl_3$) of compound **(*S*)-MPA-*anti*-(1*R*, 4*R*)-3 (T = -25ºC)**. 19

Figure S17. ^{13}C NMR spectrum (125 MHz, $CDCl_3$) of compound **(*S*)-MPA-*anti*-(1*R*, 4*R*)-3 (T = -25ºC)**. 20

Figure S18. gCOSY spectrum of compound **(*S*)-MPA-*anti*-(1*R*, 4*R*)-3 (T = -25ºC)**. 21

Figure S19. ^1H NMR spectrum (400 MHz, $CDCl_3$) of compound **(*R*)-MPA-*syn*-(1*R*, 4*S*)-3a**. 22

Figure S20. ^{13}C NMR spectrum (100 MHz, $CDCl_3$) of compound **(*R*)-MPA-*syn*-(1*R*, 4*S*)-3a**. 23

Figure S21. gCOSY spectrum of compound **(*R*)-MPA-*syn*-(1*R*, 4*S*)-3a**. 24

Figure S22. ^1H NMR spectrum (400 MHz, $CDCl_3$) of compound **(*S*)-MPA-*syn*-(1*R*, 4*S*)-3a**. 25

Figure S23. ^{13}C NMR spectrum (100 MHz, $CDCl_3$) of compound **(*S*)-MPA-*syn*-(1*R*, 4*S*)-3a**. 26

Figure S24. gCOSY spectrum of compound **(*S*)-MPA-*syn*-(1*R*, 4*S*)-3a**. 27

Figure S25. ^1H NMR spectrum (400 MHz, $CDCl_3$) of compound **(*R*)-MPA-(*S*)-4a**. 28

Figure S26. gCOSY spectrum of compound **(*R*)-MPA-(*S*)-4a**. 29

Figure S27. ^1H NMR spectrum (400 MHz, $CDCl_3$) of compound **(*S*)-MPA-(*S*)-4a**. 30

Figure S28. gCOSY spectrum of compound **(*S*)-MPA-(*S*)-4a.** 31

Figure S29. *ee* Determination of thiochroman-4-ol (**1**) from *P. lilacinum* BC17-2 (Chromatogram I) and *E. maritima* BC17 (Chromatogram II). 32

Figure S30. *ee* Determination of *syn*-thiochroman-4-ol 1-oxide (***syn*-3**) from *P. lilacinum* BC17-2 (Chromatogram I) and *E. maritima* BC17 (Chromatogram II). 33

Figure S31. *ee* Determination of *anti*-thiochroman-4-ol 1-oxide (***anti*-3**) from *P. lilacinum* BC17-2 (Chromatogram I) and *E. maritima* BC17 (Chromatogram II). 34

Figure S32. *ee* Determination of thiochroman-4-ol 1,1-dioxide (**4**) from *P. lilacinum* BC17-2.35

Figure S33. *ee* Determination of thiochroman-4-one 1-oxide **(5**) from *P. lilacinum* BC17-2. .. 36

Figure S34. *ee* Determination of 6-chlorothiochroman-4-ol (**1a**) from *P. lilacinum* BC17-2 (Chromatogram I) and *E. maritima* BC17 (Chromatogram II). 37

Figure S35. *ee* Determination of *syn*-6-chlorothiochroman-4-ol 1-oxide (***syn*-3a**) from *P. lilacinum* BC17-2 (Chromatogram I) and *E. maritima* BC17 (Chromatogram II). 38

Figure S36. *ee* Determination of *anti*-6-chlorothiochroman-4-ol 1-oxide (***anti*-3a**) from *P. lilacinum* BC17-2 (Chromatogram I) and *E. maritima* BC17 (Chromatogram II). 39

Figure S37. *ee* Determination of 6-chlorothiochroman-4-ol 1,1-dioxide (**4a**) from *P. lilacinum* BC17-2. 40

Figure S38. *ee* Determination of 6-chlorothiochroman-4-one 1-oxide (**5a**) from *P. lilacinum* BC17-2. 41

Figure S39. *ee* Determination of 1-(5-chloro-2-(methylthio)phenyl)propane-1,3-diol (**6a**) from *P. lilacinum* BC17-2.. 42

Figure S40. *ed* Determination of thiochroman-4-ol 1-oxide (**3**) diastereoisomers from *P. lilacinum* BC17-2 (Chromatogram I) and *E. maritima* BC17 (Chromatogram II). 43

Figure S41. *ed* Determination of 6-chlorothiochroman-4-ol 1-oxide (**3a**) diastereoisomers from *P. lilacinum* BC17-2 (Chromatogram I) and *E. maritima* BC17 (Chromatogram II). 44

Figure S42. *In vitro* antimicrobial assays. 45

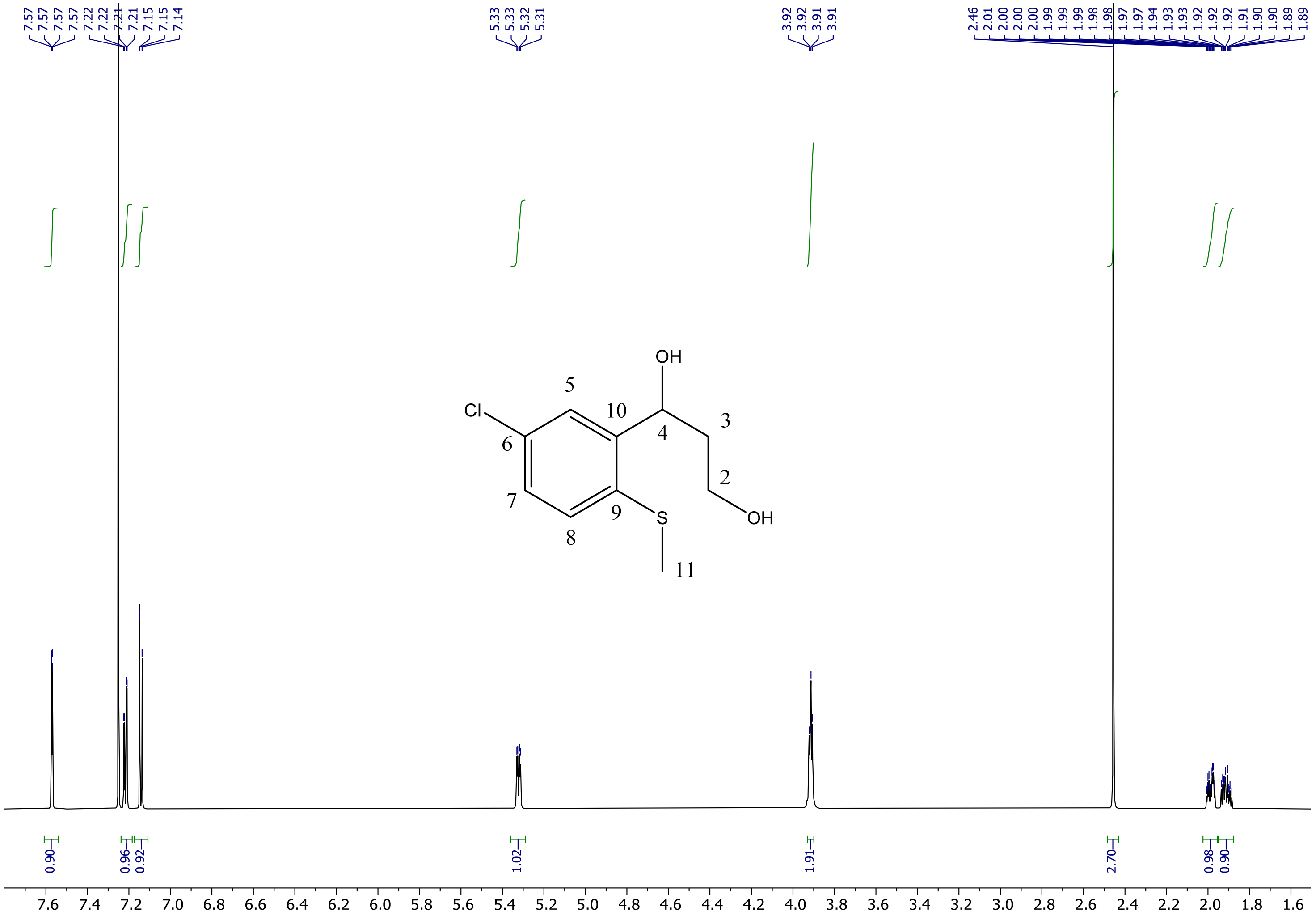

Figure S1. ^{1}H NMR spectrum (700 MHz, $CDCl_3$) of compound **6a**.

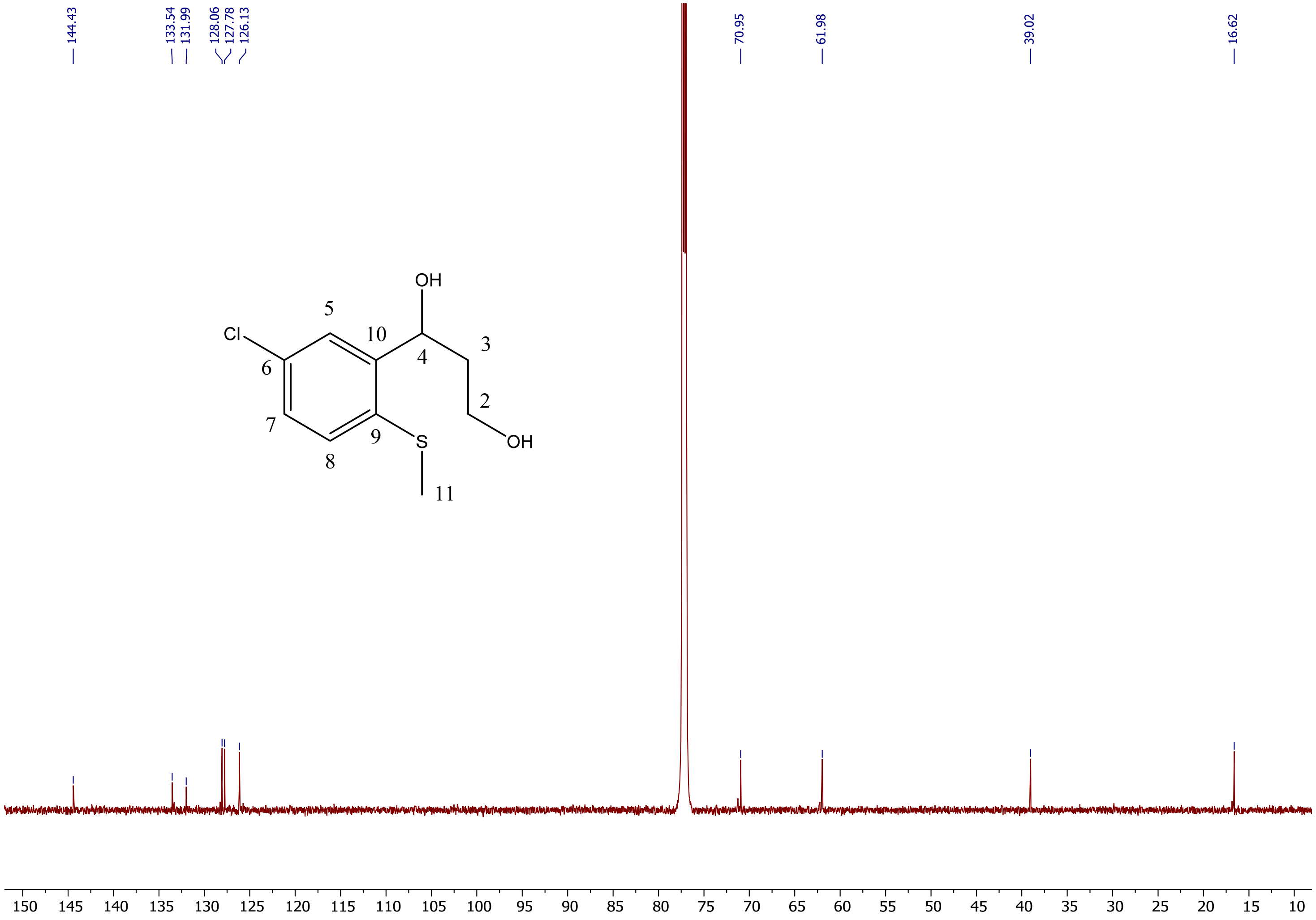

Figure S2. ^{13}C NMR spectrum (175 MHz, $CDCl_3$) of compound **6a**.

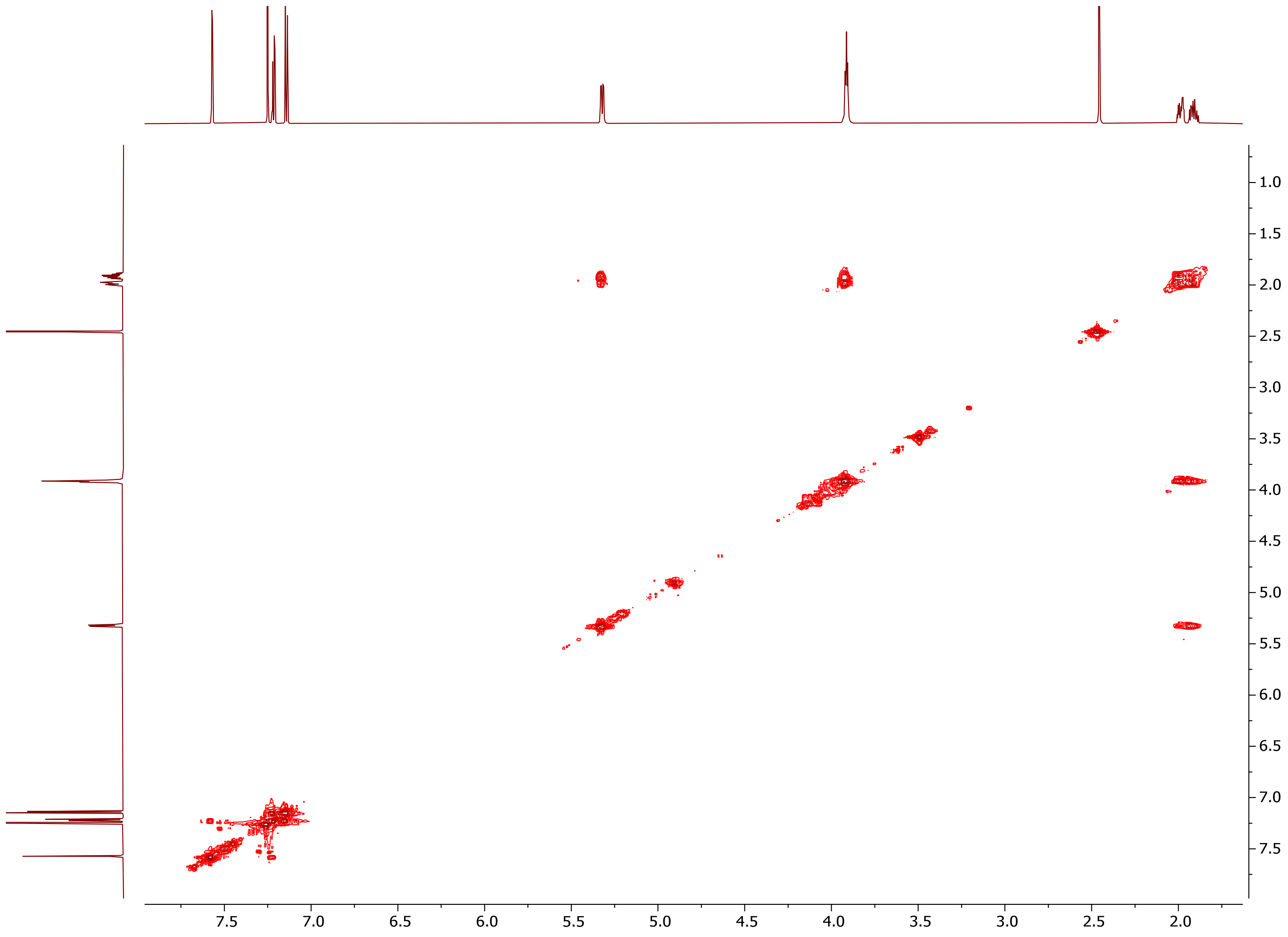

Figure S3. gCOSY spectrum of compound **6a**.

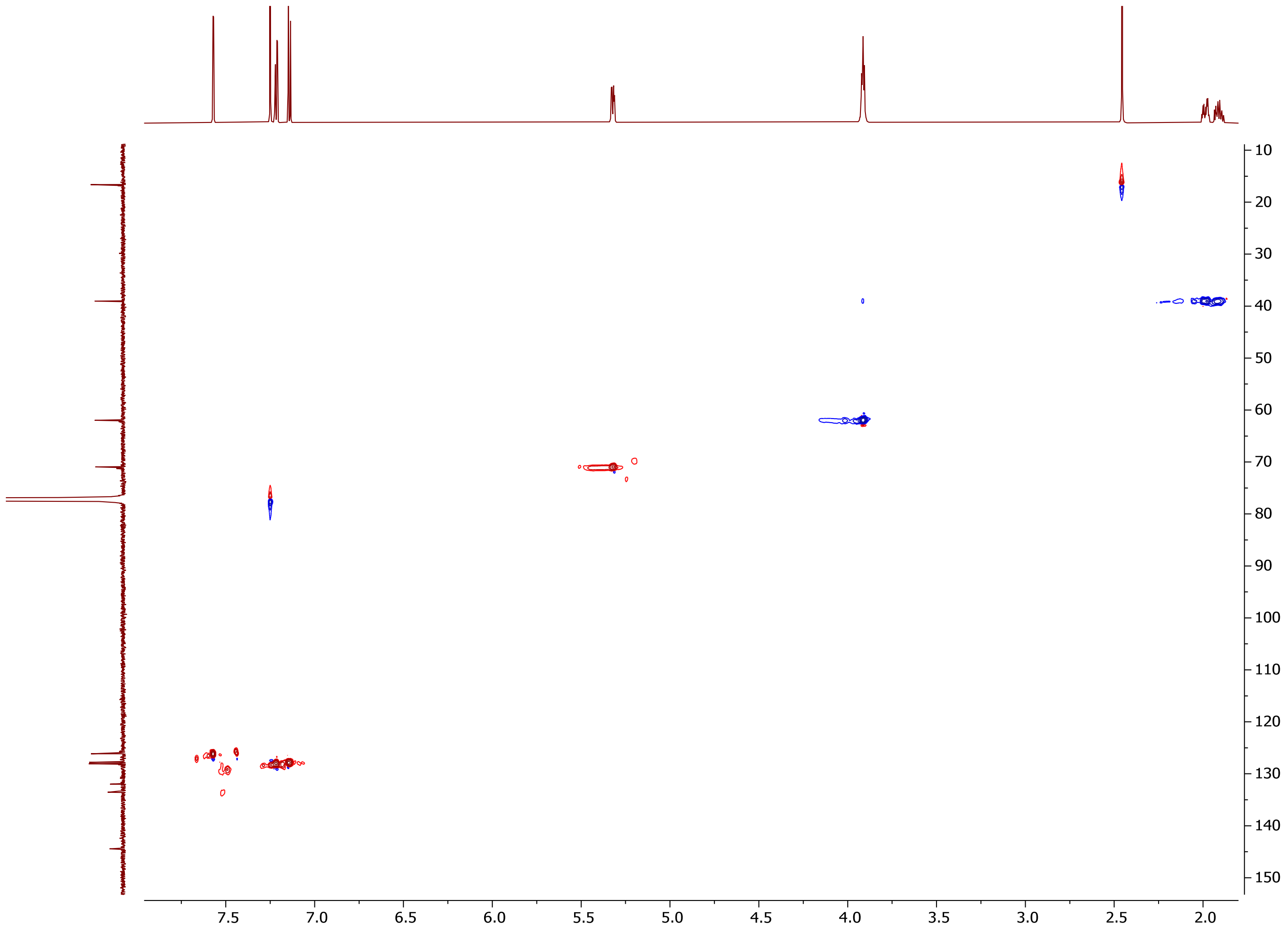

Figure S4. gHSQC spectrum of compound **6a**.

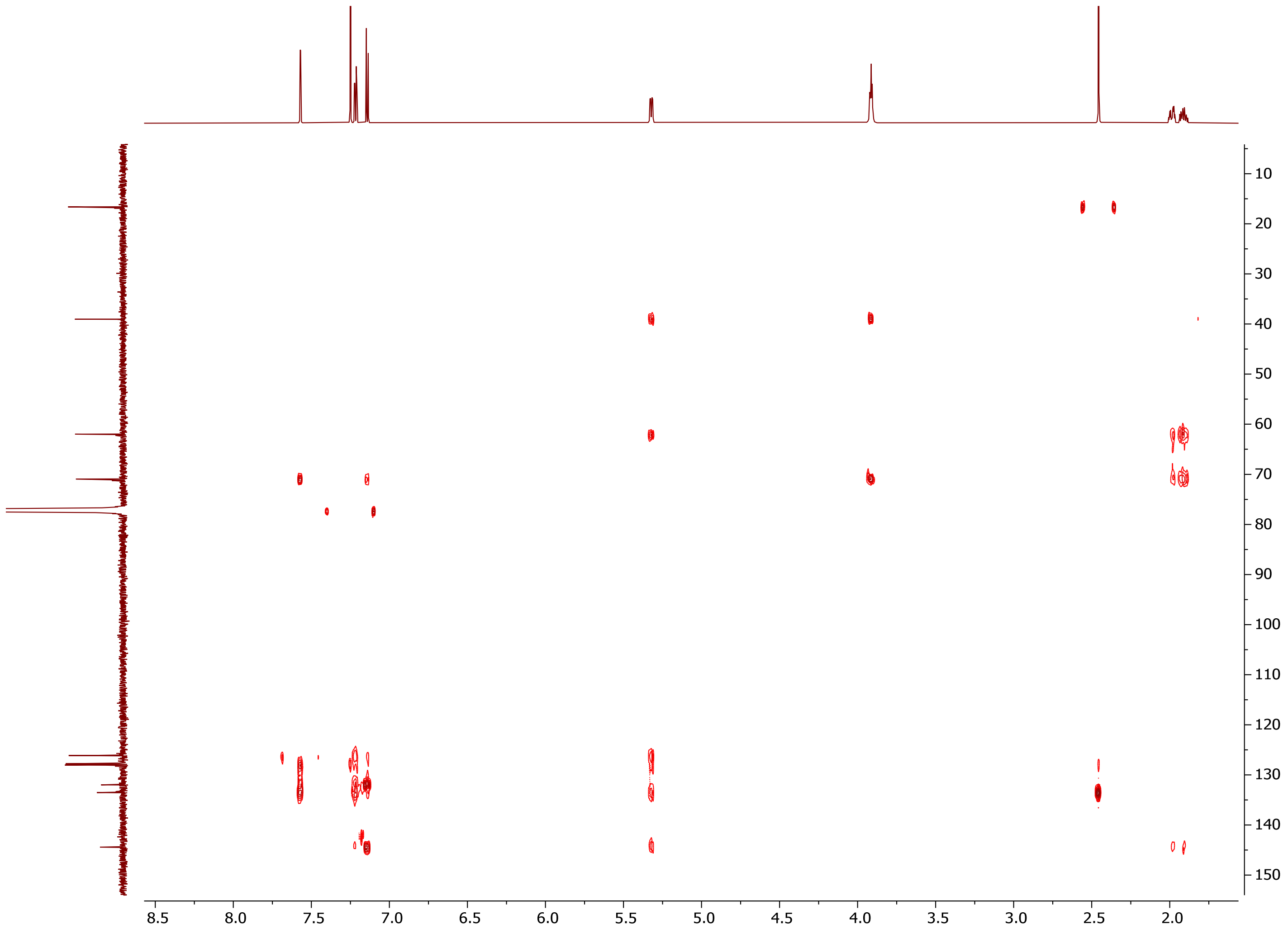

Figure S5. gHMBC spectrum of compound **6a**.

Monoisotopic Mass, Even Electron Ions
100 formula(e) evaluated with 8 results within limits (up to 50 best isotopic matches for each mass)
Elements Used:
C: 0-25 H: 0-100 O: 0-3 Na: 0-1 S: 0-1 Cl: 0-1

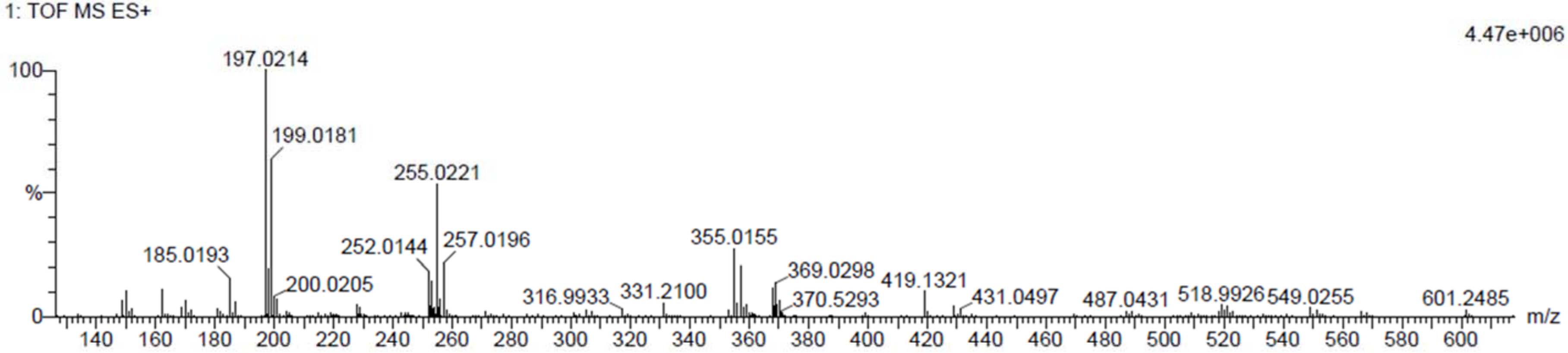

Minimum:				-1.5				
Maximum:		5.0	10.0	50.0				
Mass	Calc. Mass	mDa	PPM	DBE	i-FIT	Norm	Conf(%)	Formula
255.0221	255.0222	-0.1	-0.4	3.5	159.1	0.116	89.09	C10 H13 O2 Na S Cl

Figure S6. HR ESI-MS spectrum of compound **6a**.

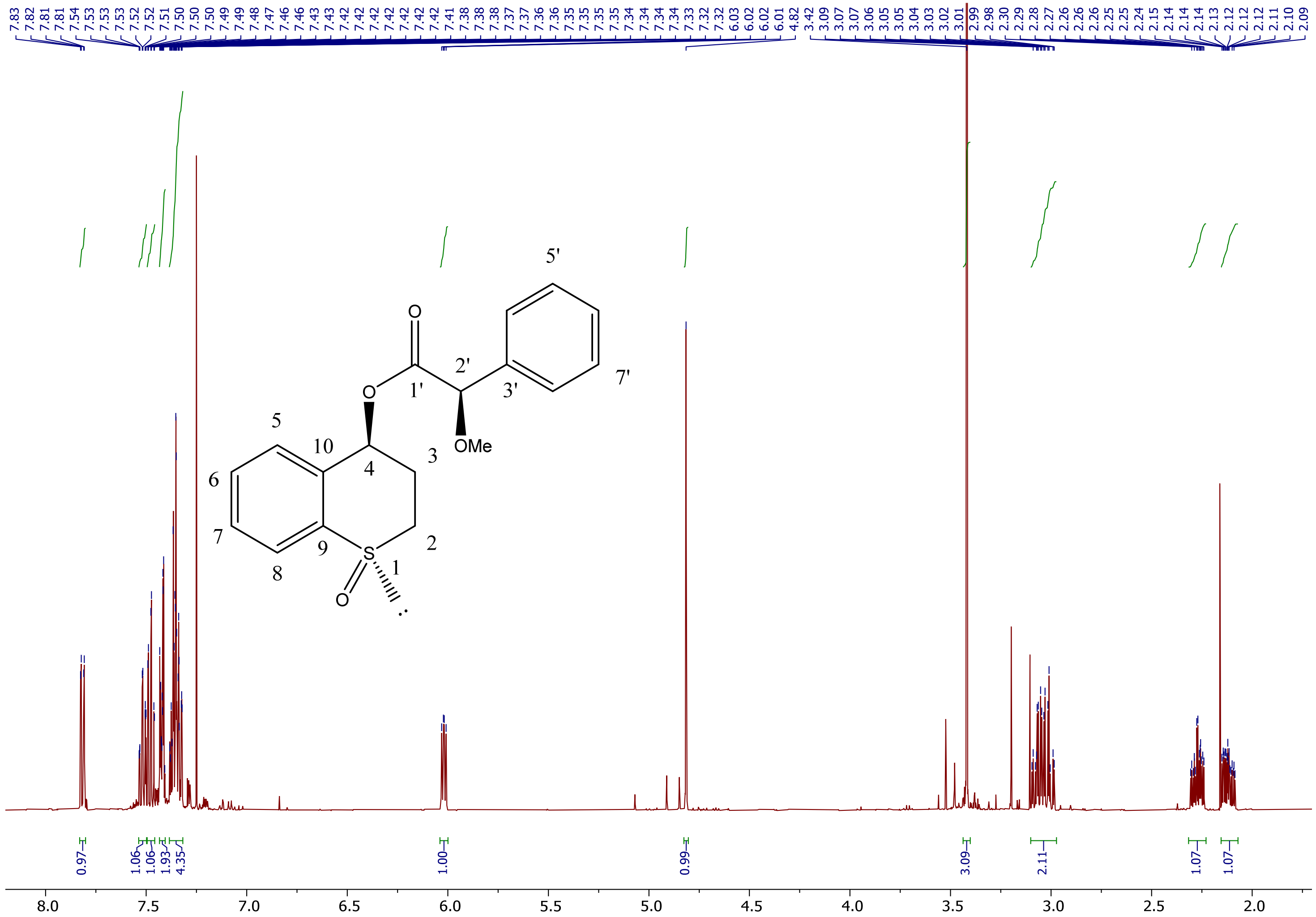

Figure S7. ^{1}H NMR spectrum (500 MHz, $CDCl_3$) of compound ***(R)*-MPA-*syn*-(1*R*, 4*S*)-3.**

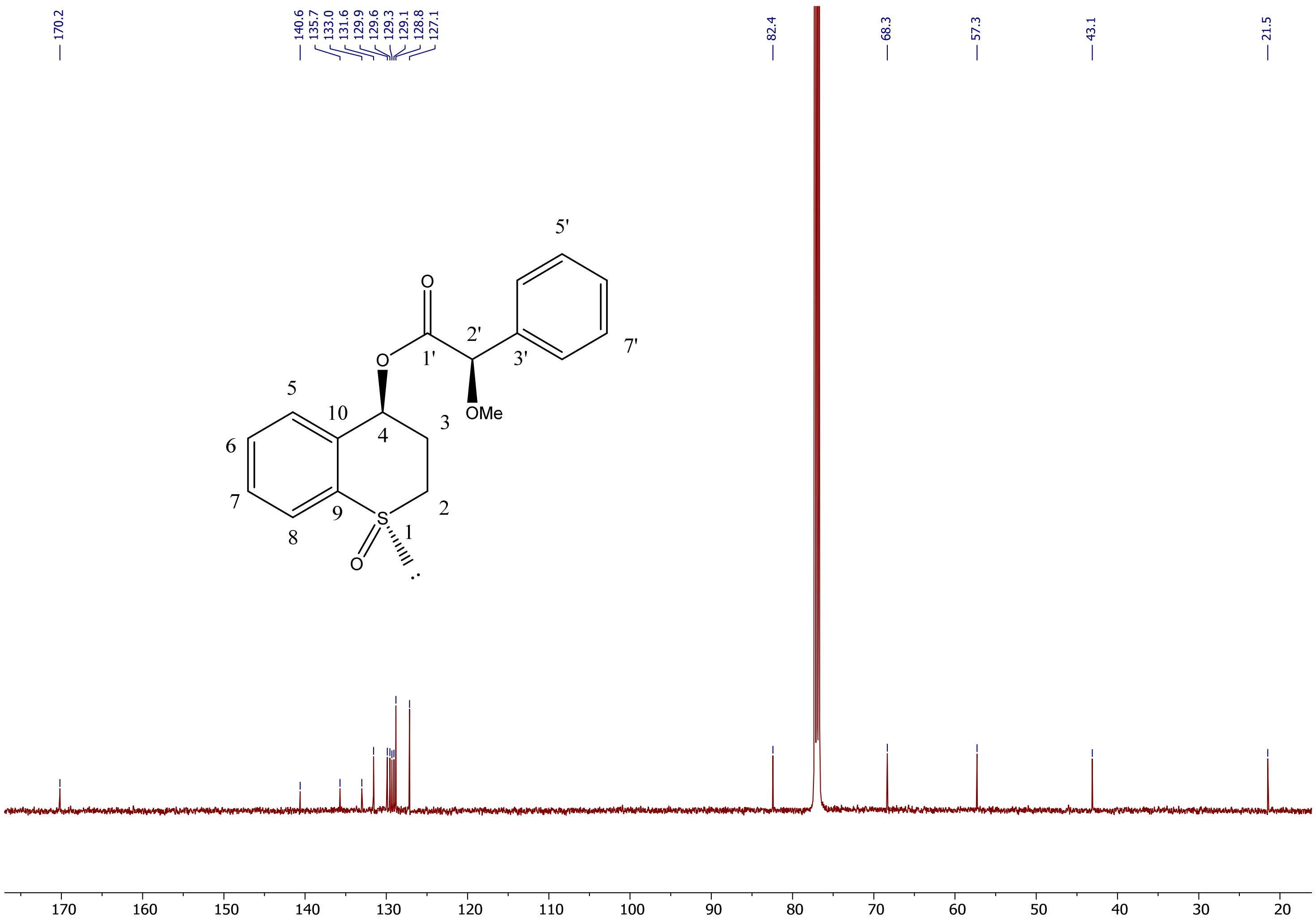

Figure S8. ^{13}C NMR spectrum (125 MHz, $CDCl_3$) of compound **(*R*)-MPA-*syn*-(1*R*, 4*S*)-3**.

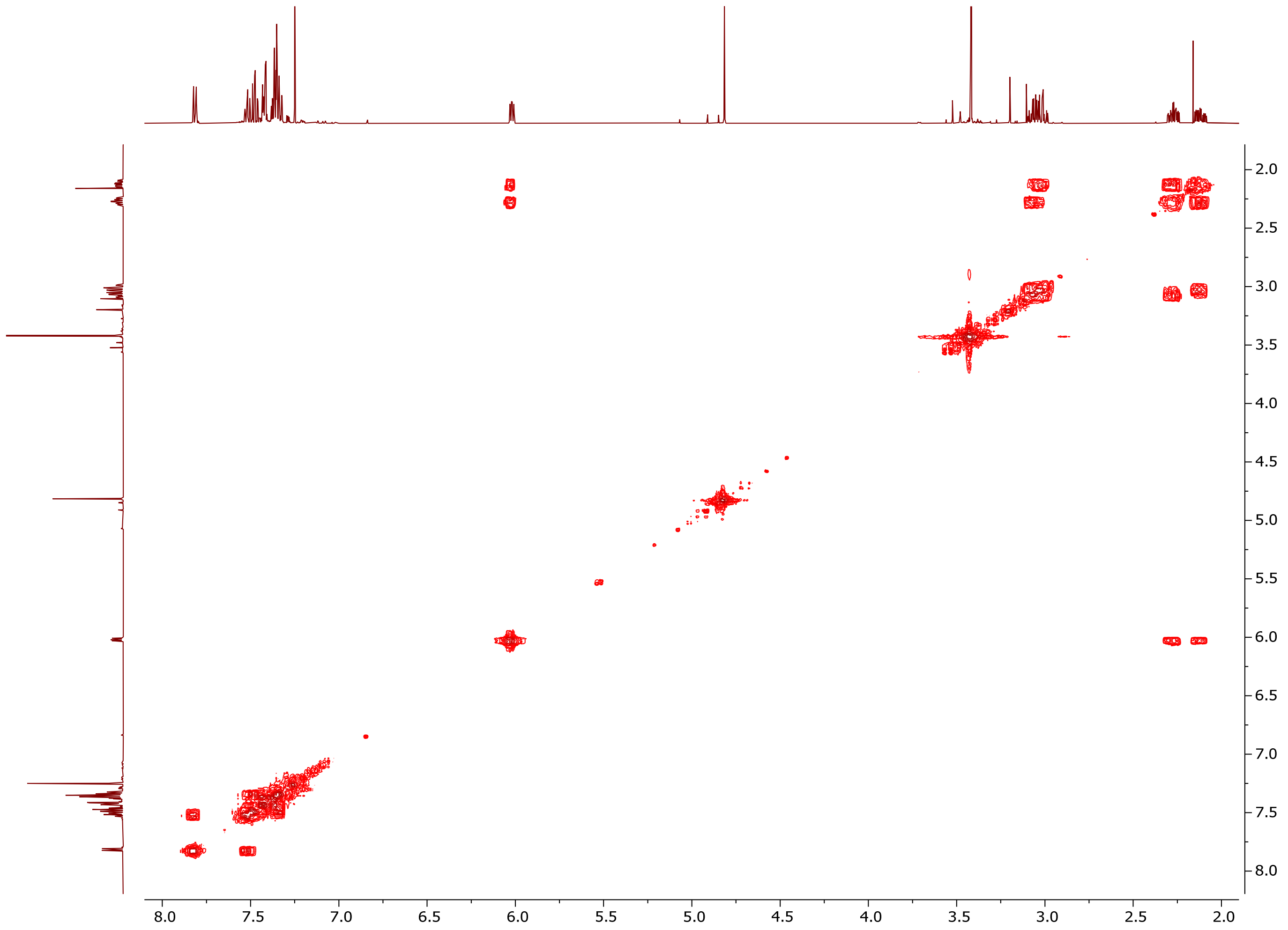

Figure S9. gCOSY spectrum of compound **(*R*)-MPA-*syn*-(1*R*, 4*S*)-3**.

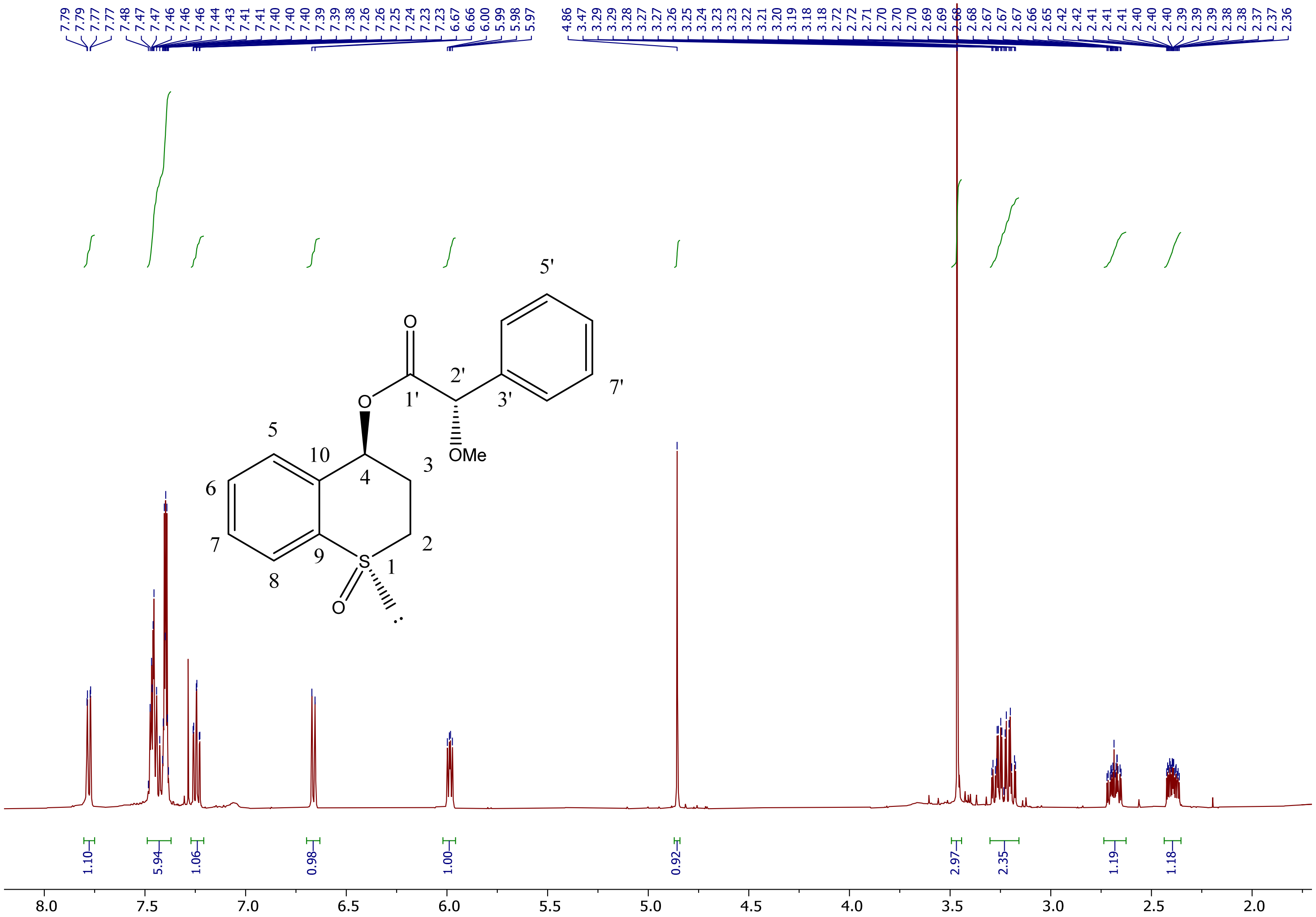

Figure S10. ^{1}H NMR spectrum (500 MHz, $CDCl_3$) of compound **(*S*)-MPA-*syn*-(1*R*, 4*S*)-3**.

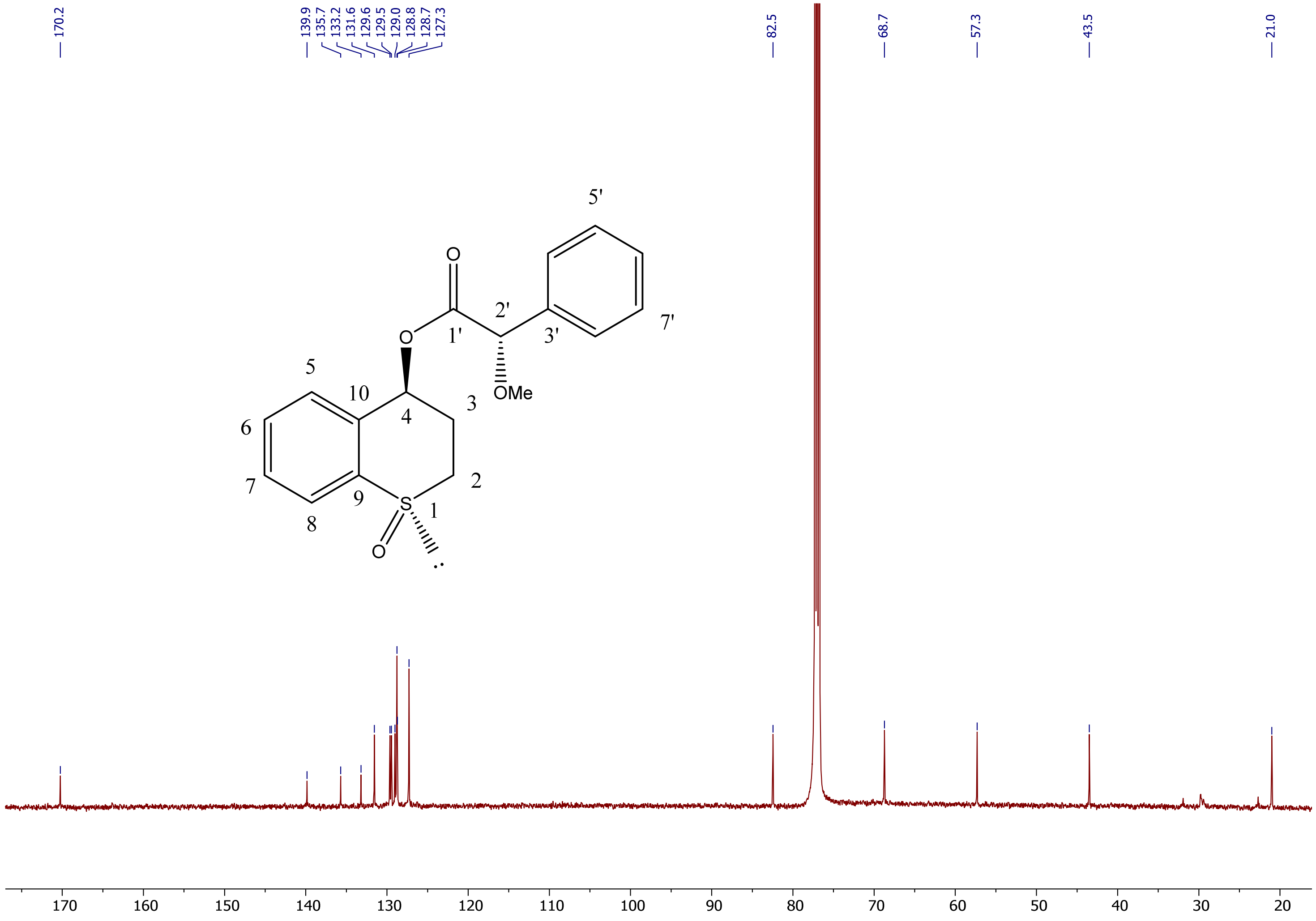

Figure S11. ^{13}C NMR spectrum (125 MHz, $CDCl_3$) of compound **(*S*)-MPA-*syn*-(1*R*, 4*S*)-3**.

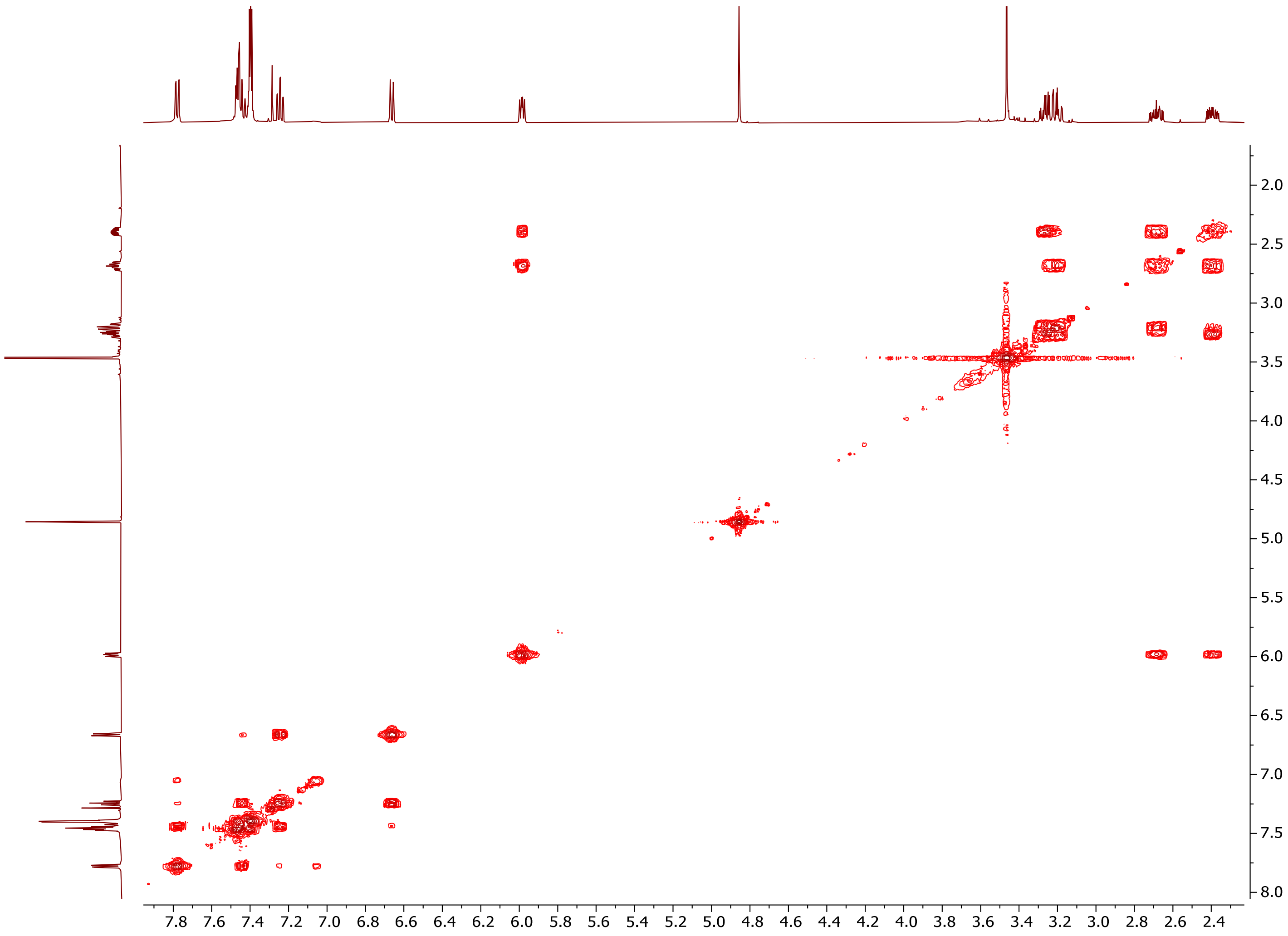

Figure S12. gCOSY spectrum of compound **(*S*)-MPA-*syn*-(1*R*, 4*S*)-3**.

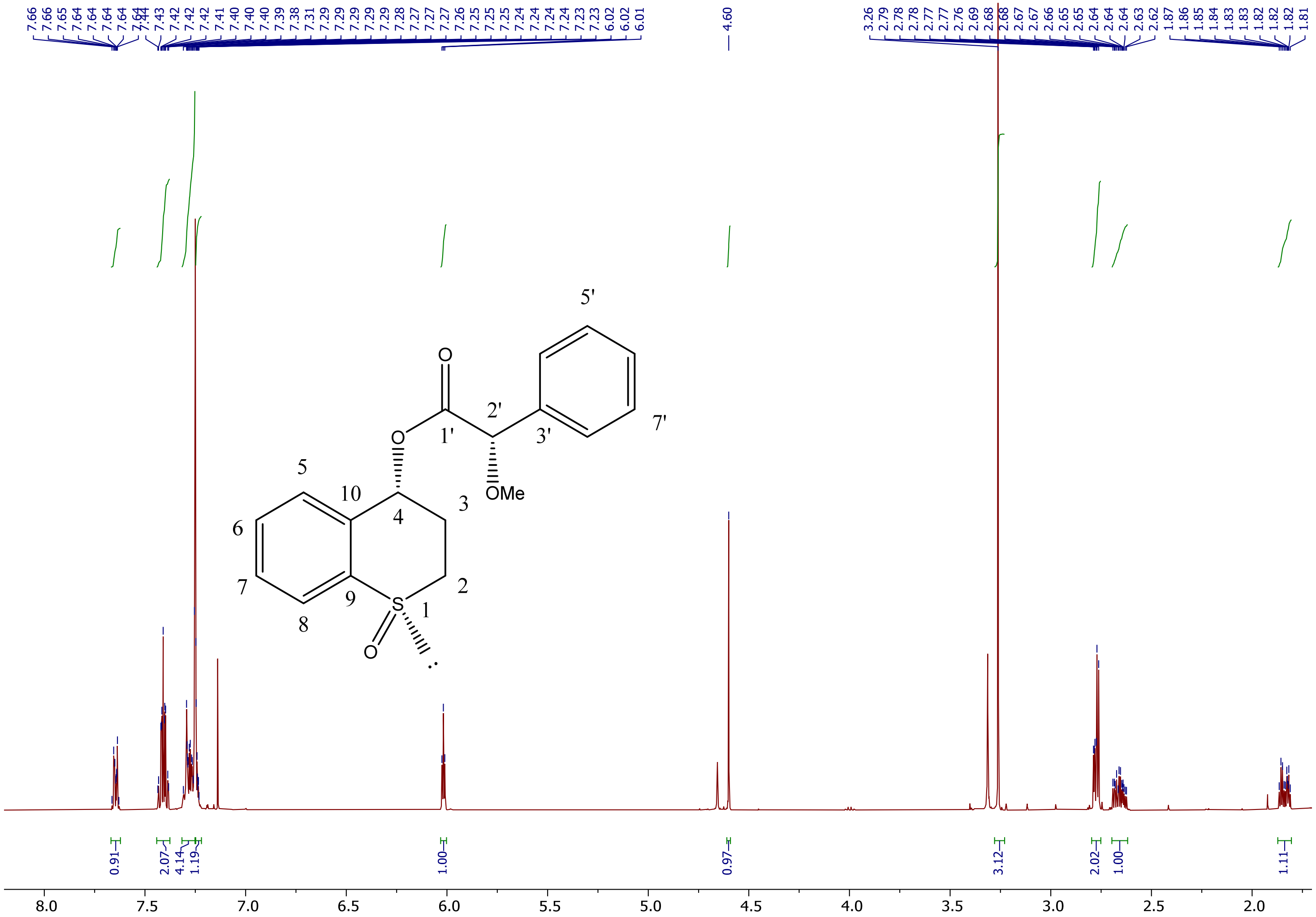

Figure S13. ^{1}H NMR spectrum (500 MHz, $CDCl_3$) of compound **(*S*)-MPA-*anti*-(1*R*, 4*R*)-3 (T = 25ºC)**.

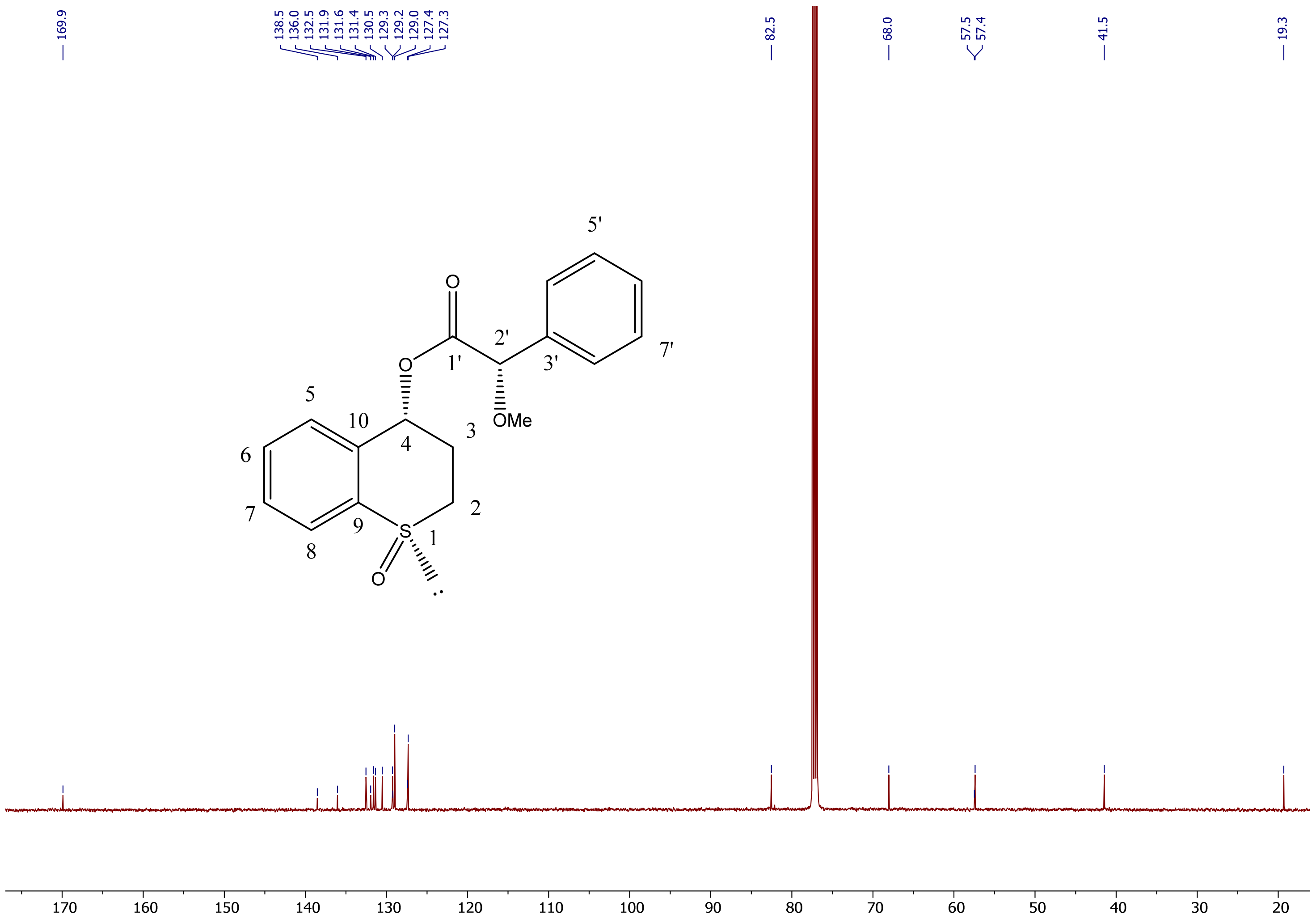

Figure S14. ^{13}C NMR spectrum (125 MHz, $CDCl_3$) of compound **(*S*)-MPA-*anti*-(1*R*, 4*R*)-3 (T = 25ºC)**.

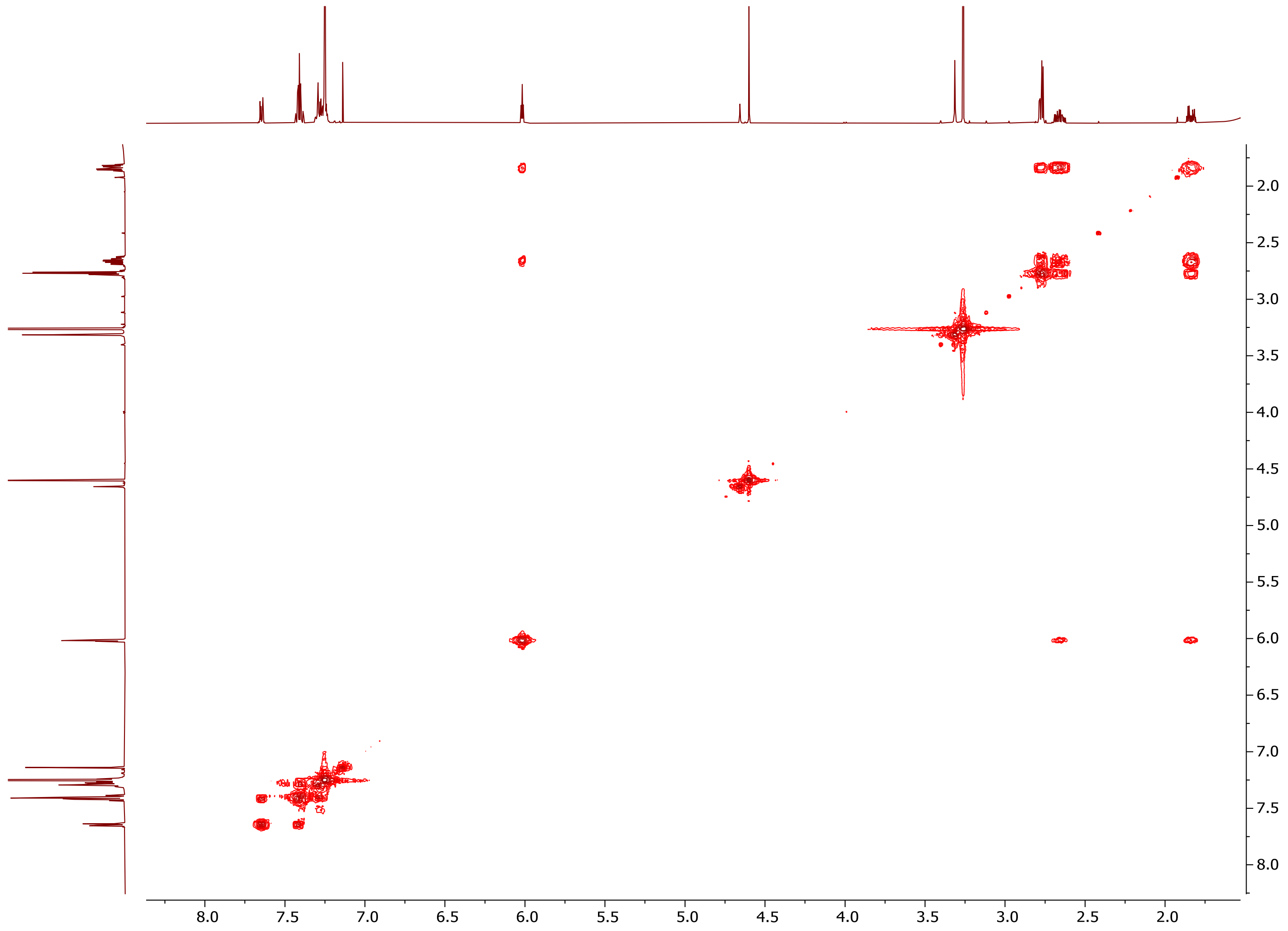

Figure S15. gCOSY spectrum of compound **(*S*)-MPA-*anti*-(1*R*, 4*R*)-3 (T = 25ºC)**.

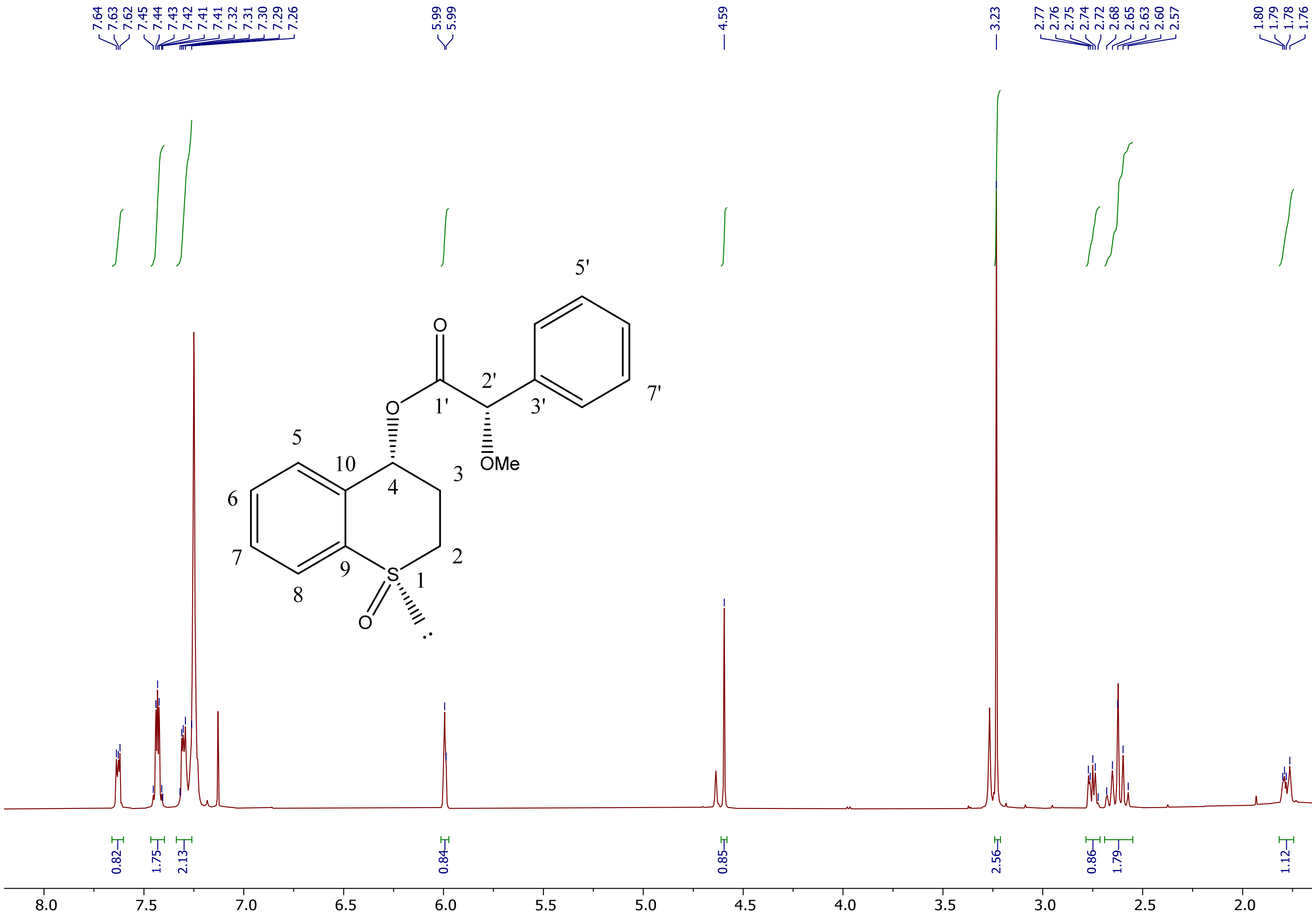

Figure S16. ^{1}H NMR spectrum (500 MHz, $CDCl_3$) of compound **(*S*)-MPA-*anti*-(1*R*, 4*R*)-3 (T = -25ºC)**.

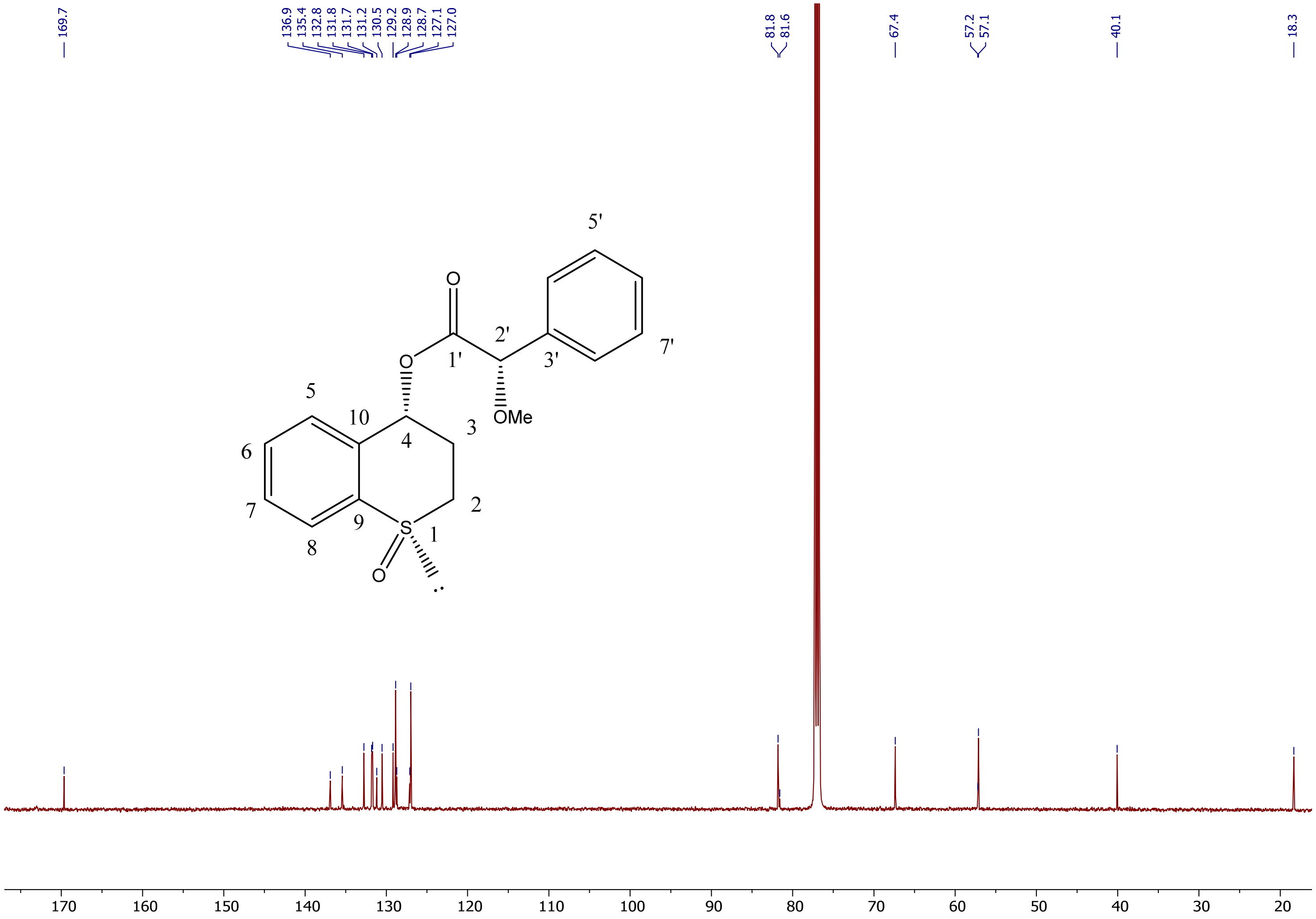

Figure S17. ^{13}C NMR spectrum (125 MHz, $CDCl_3$) of compound **(*S*)-MPA-*anti*-(1*R*, 4*R*)-3 (T = -25ºC)**.

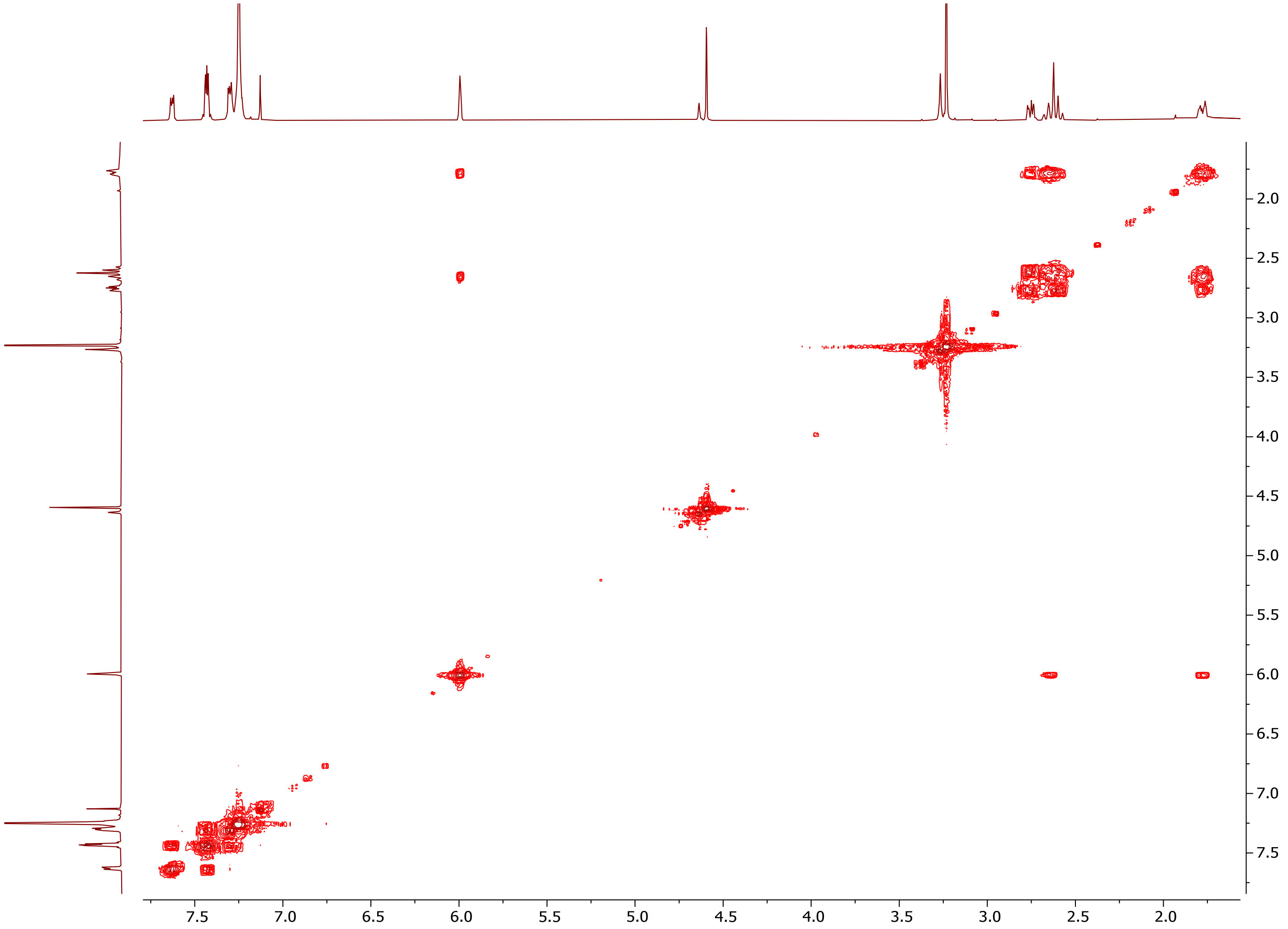

Figure S18. gCOSY spectrum of compound **(*S*)-MPA-*anti*-(1*R*, 4*R*)-3 (T = -25ºC)**.

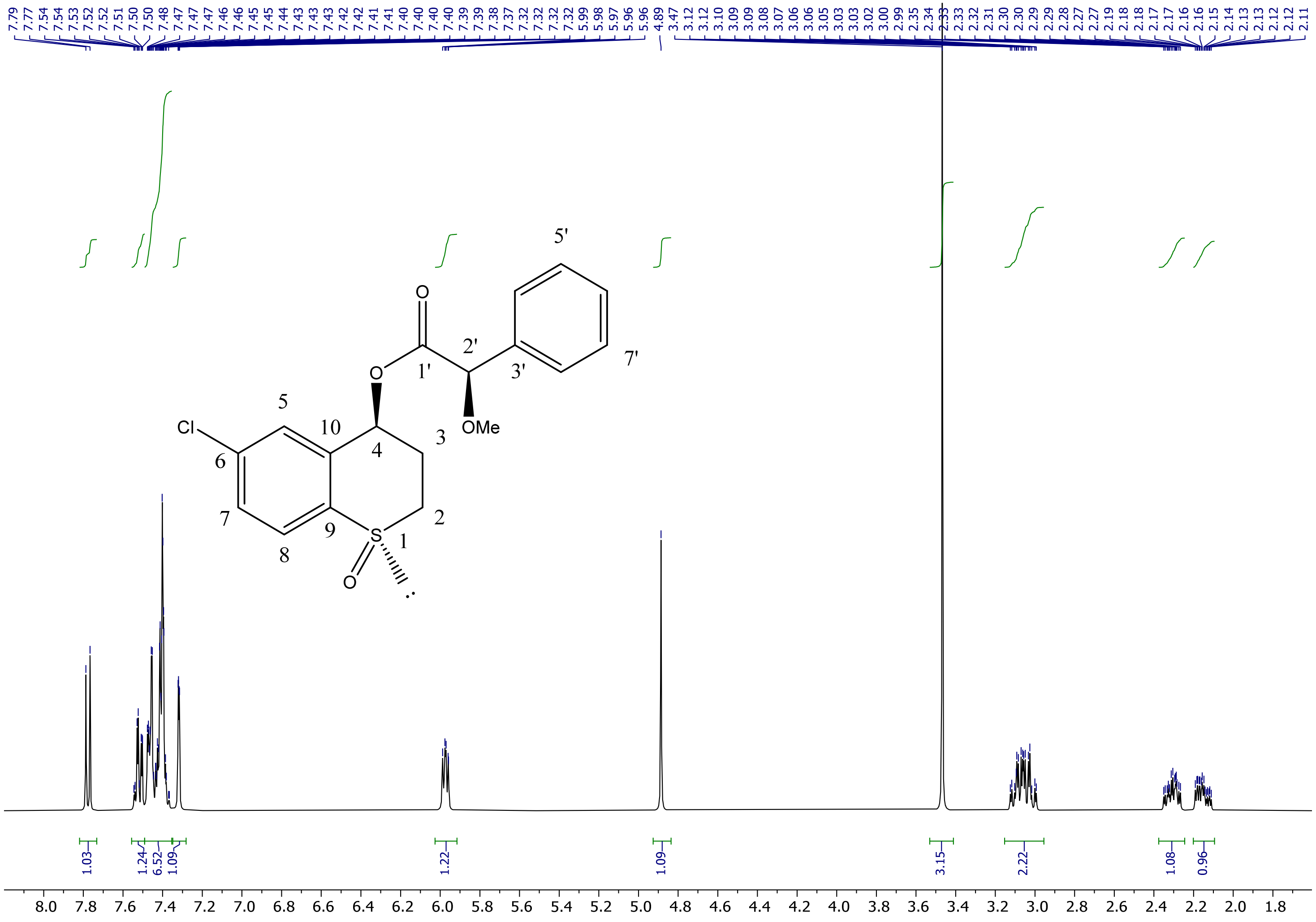

Figure S19. ^{1}H NMR spectrum (400 MHz, $CDCl_3$) of compound **(*R*)-MPA-*syn*-(1*R*, 4*S*)-3a**.

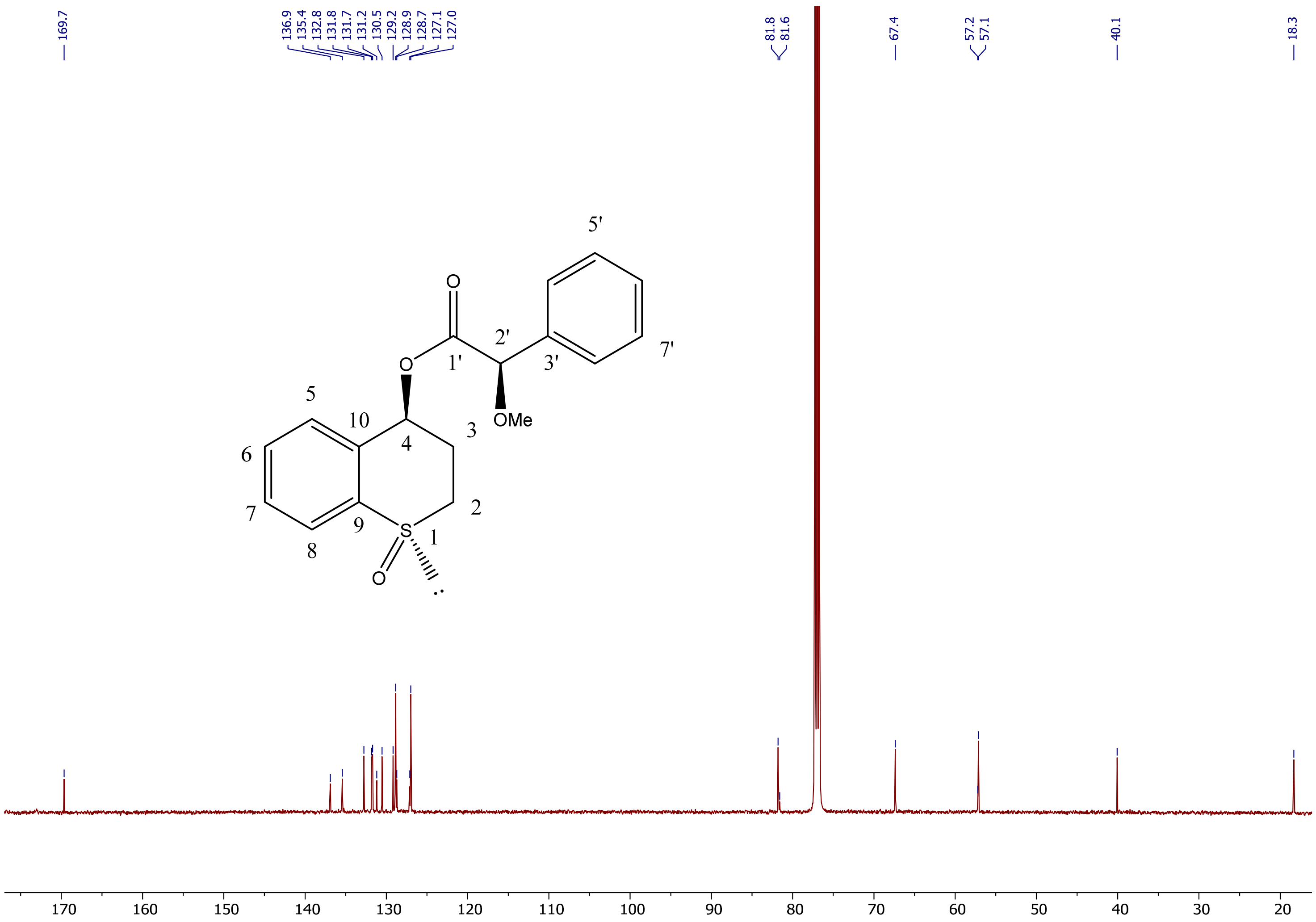

Figure S20. ^{13}C NMR spectrum (100 MHz, $CDCl_3$) of compound **(*R*)-MPA-*syn*-(1*R*, 4*S*)-3a**.

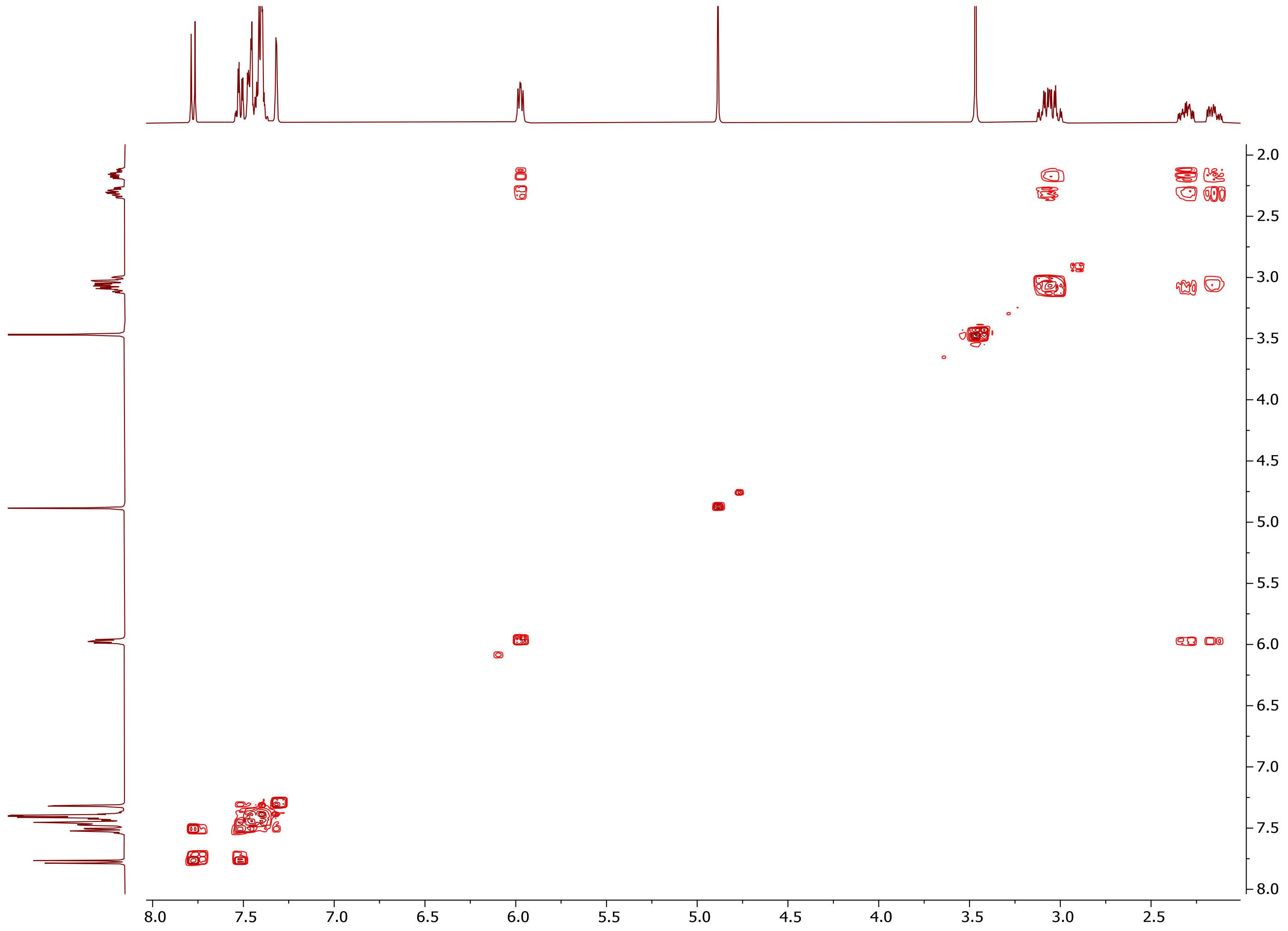

Figure S21. gCOSY spectrum of compound **(*R*)-MPA-*syn*-(1*R*, 4*S*)-3a**.

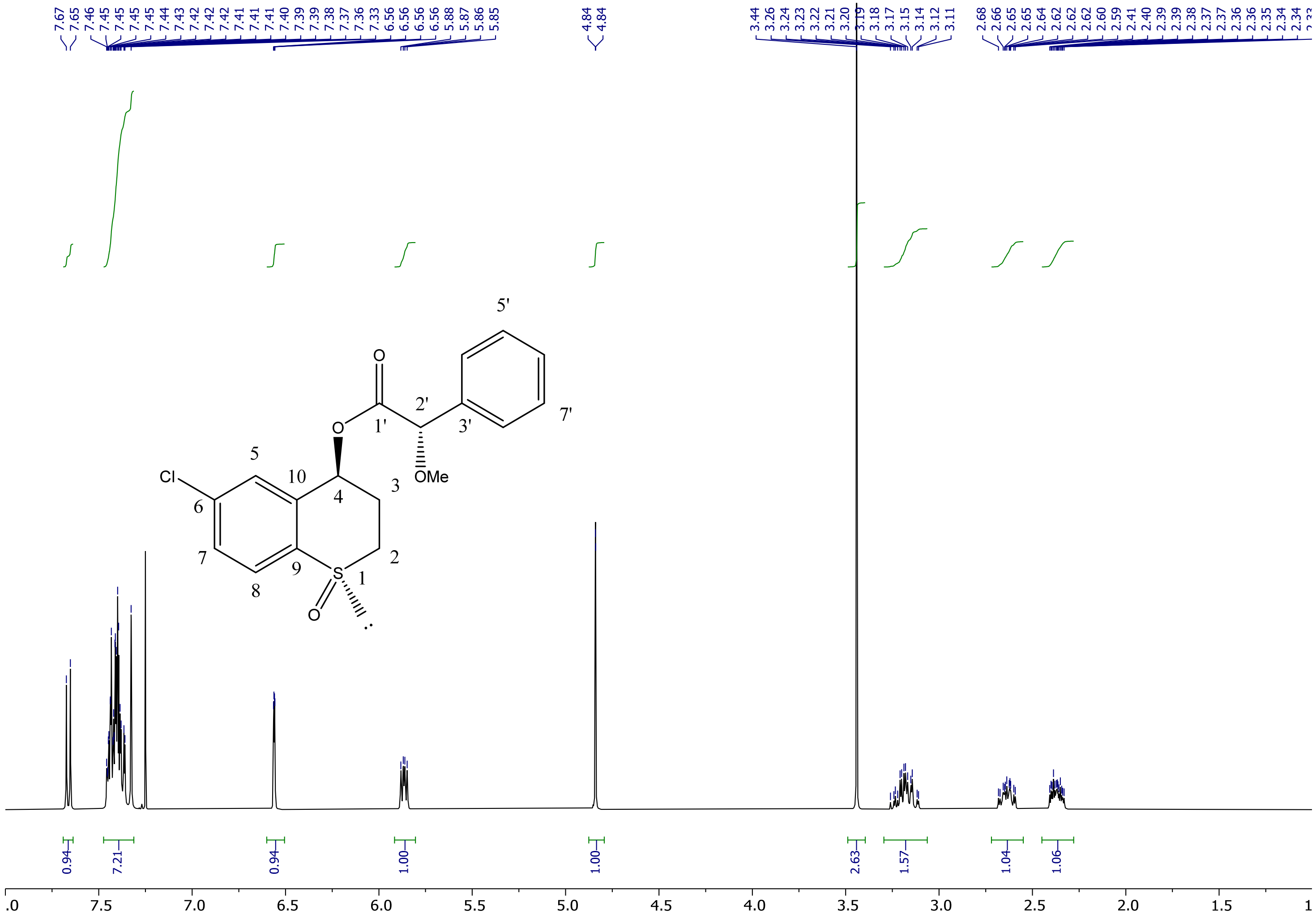

Figure S22. ^{1}H NMR spectrum (400 MHz, $CDCl_3$) of compound **(*S*)-MPA-*syn*-(1*R*, 4*S*)-3a**.

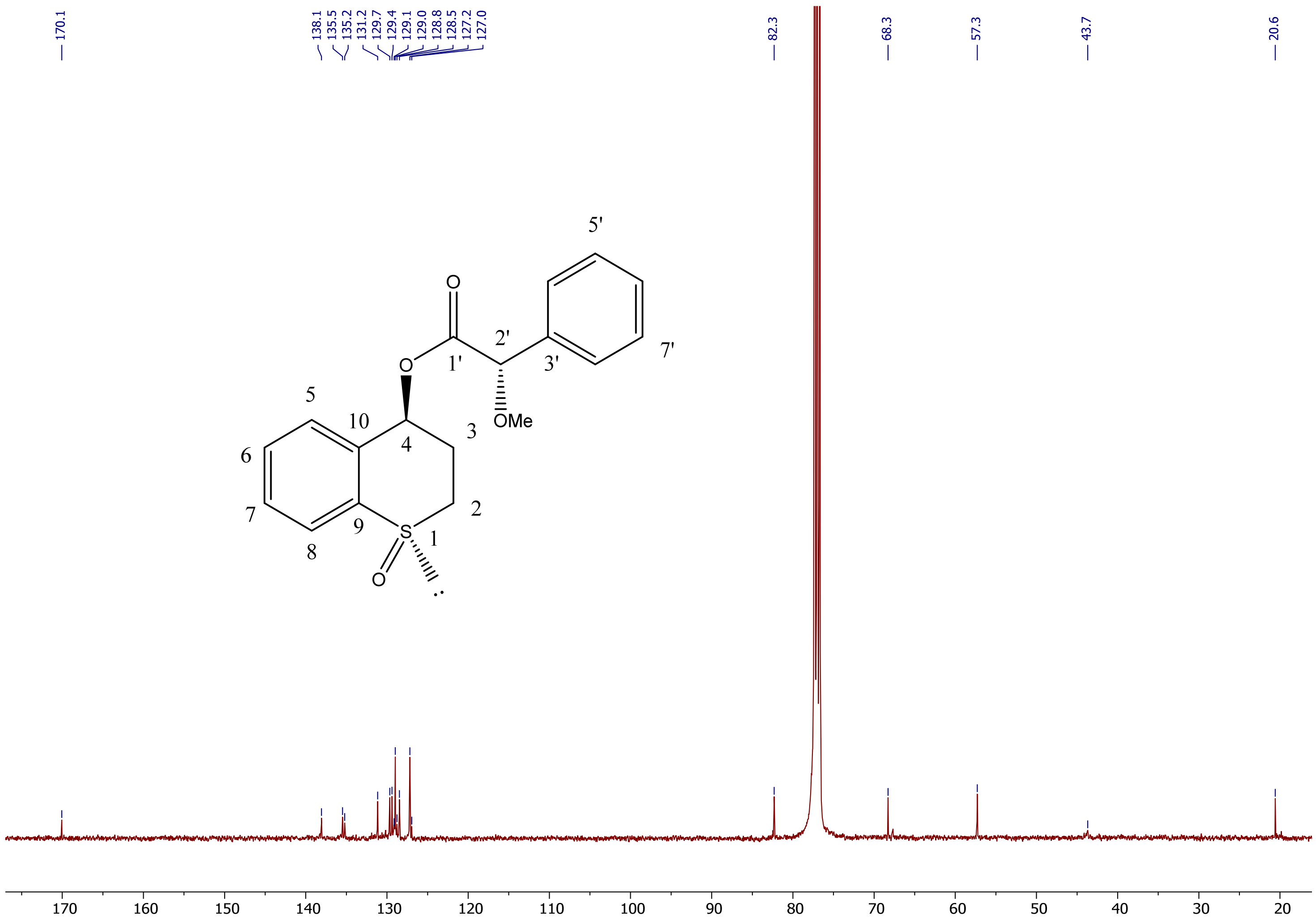

Figure S23. ^{13}C NMR spectrum (100 MHz, $CDCl_3$) of compound **(*S*)-MPA-*syn*-(1*R*, 4*S*)-3a**.

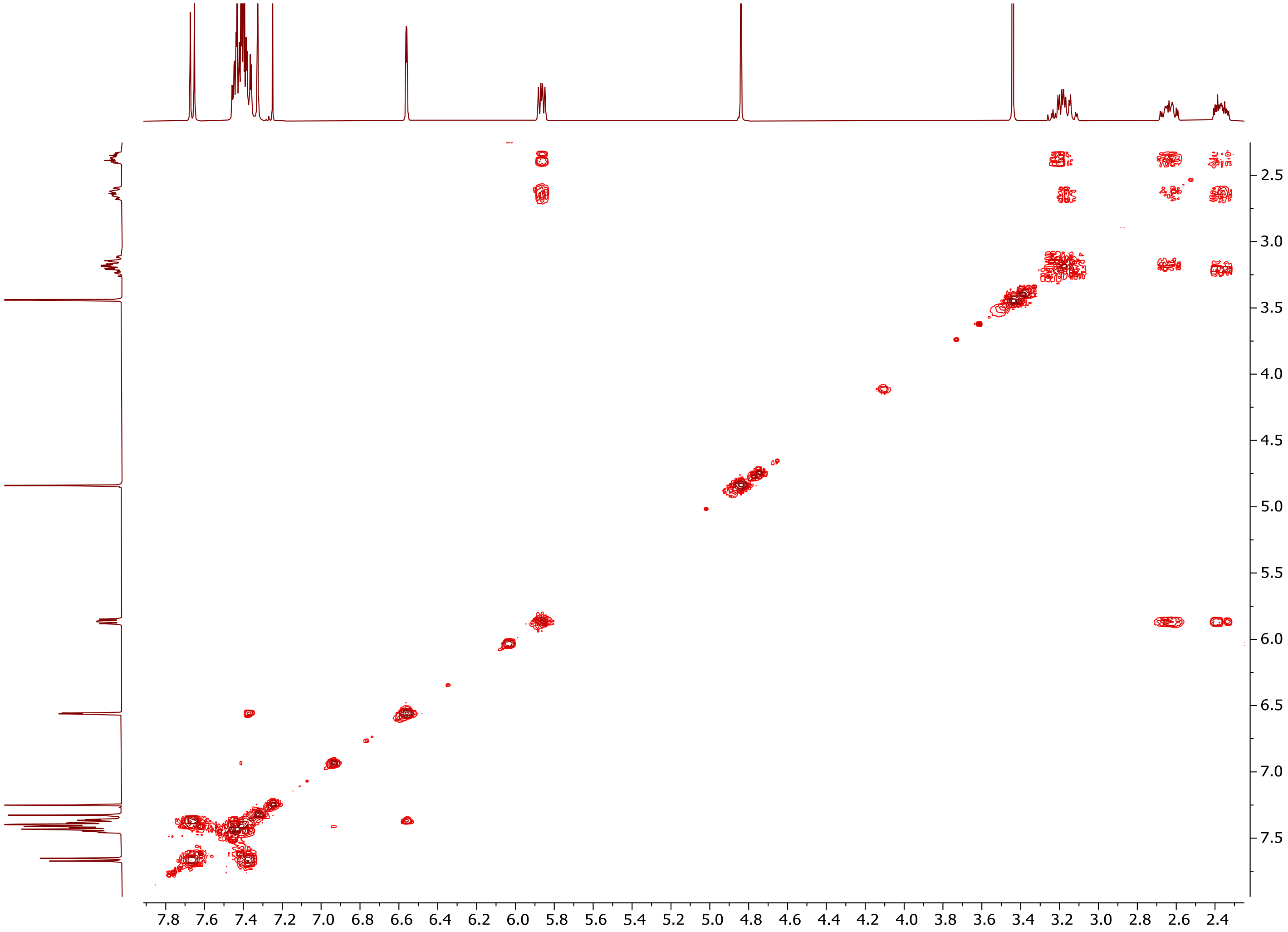

Figure S24. gCOSY spectrum of compound **(*S*)-MPA-*syn*-(1*R*, 4*S*)-3a**.

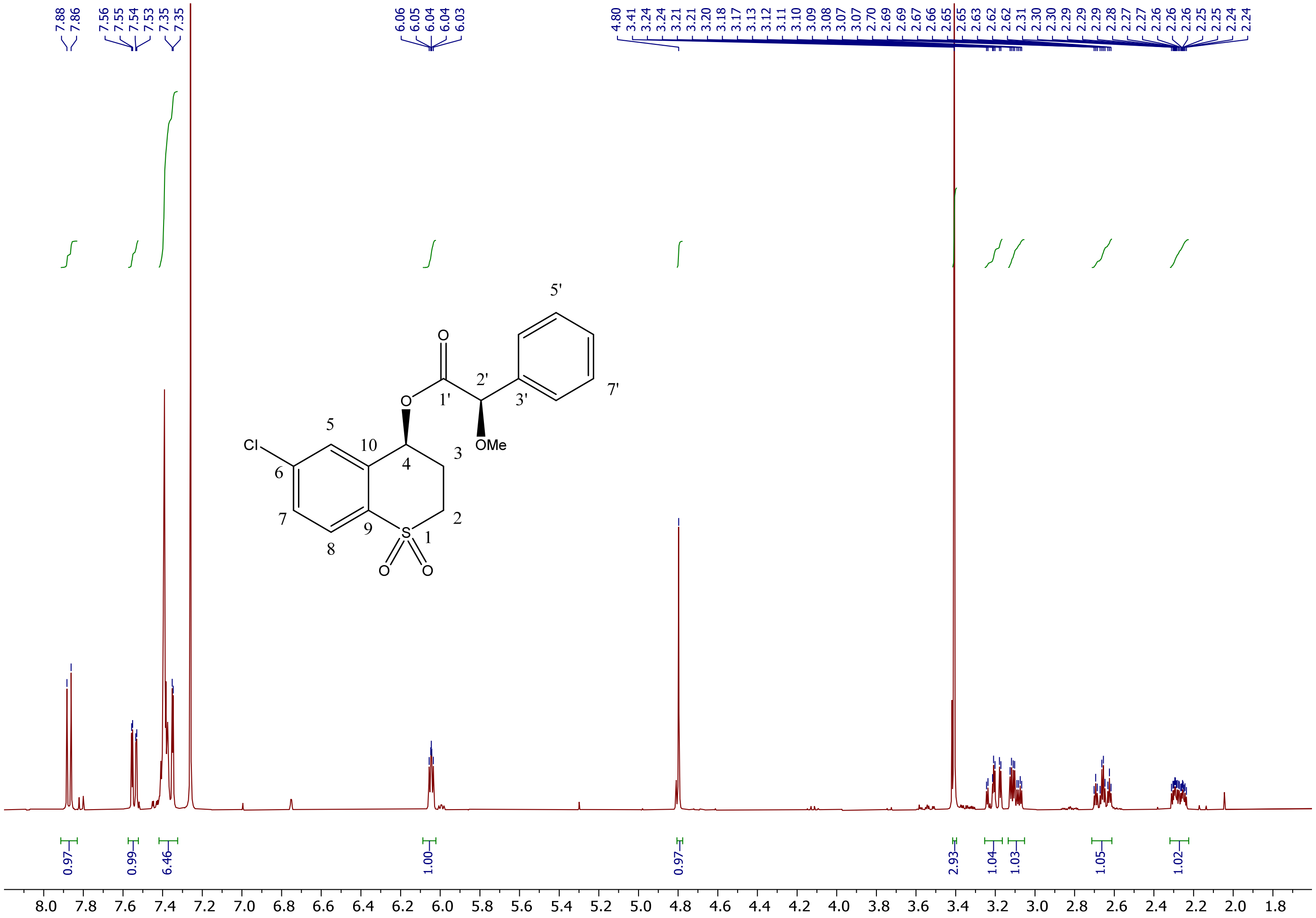

Figure S25. ^{1}H NMR spectrum (400 MHz, $CDCl_3$) of compound ***(R)*-MPA-*(S)*-4a**.

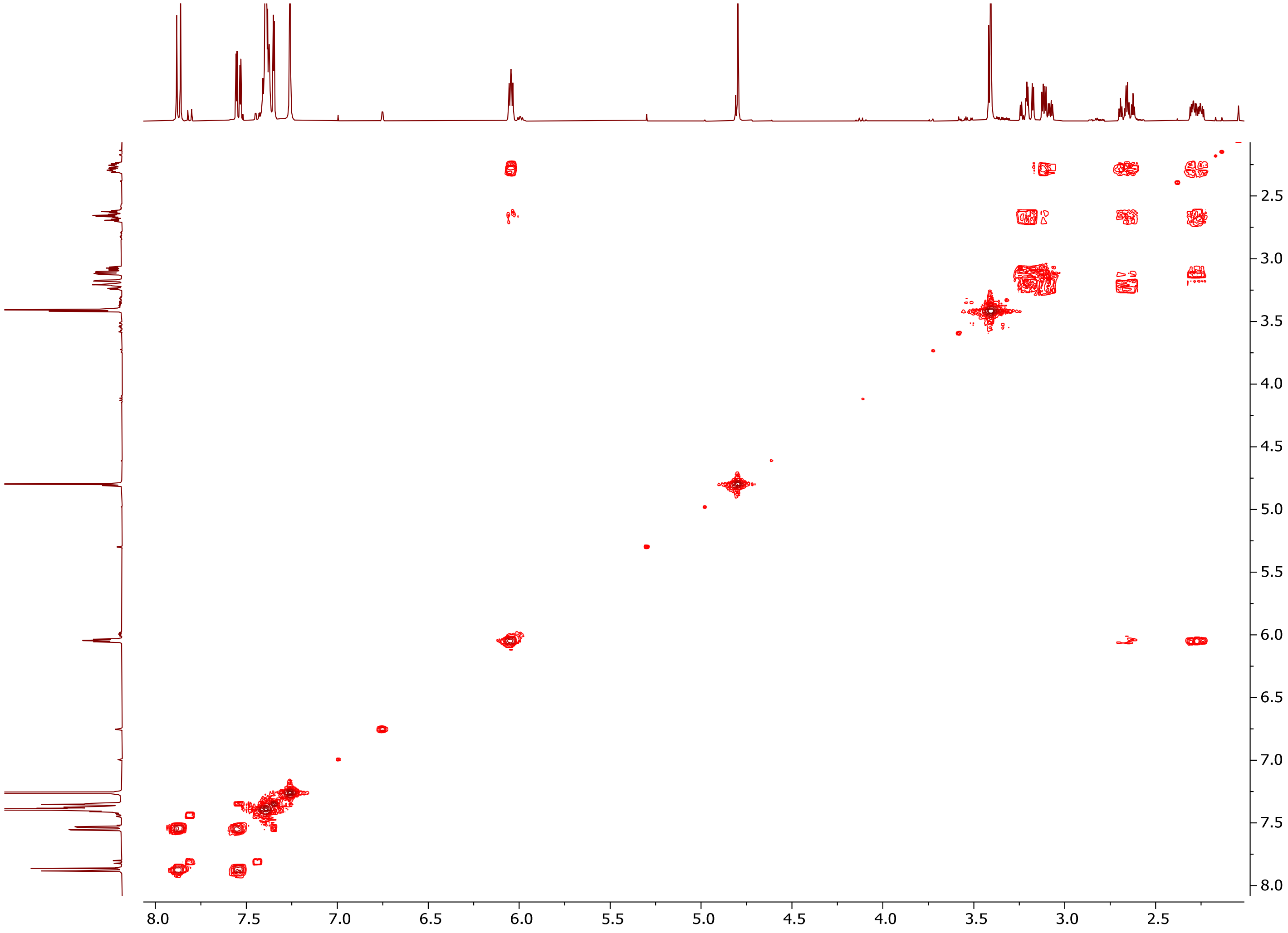

Figure S26. gCOSY spectrum of compound ***(R)*-MPA-*(S)*-4a**.

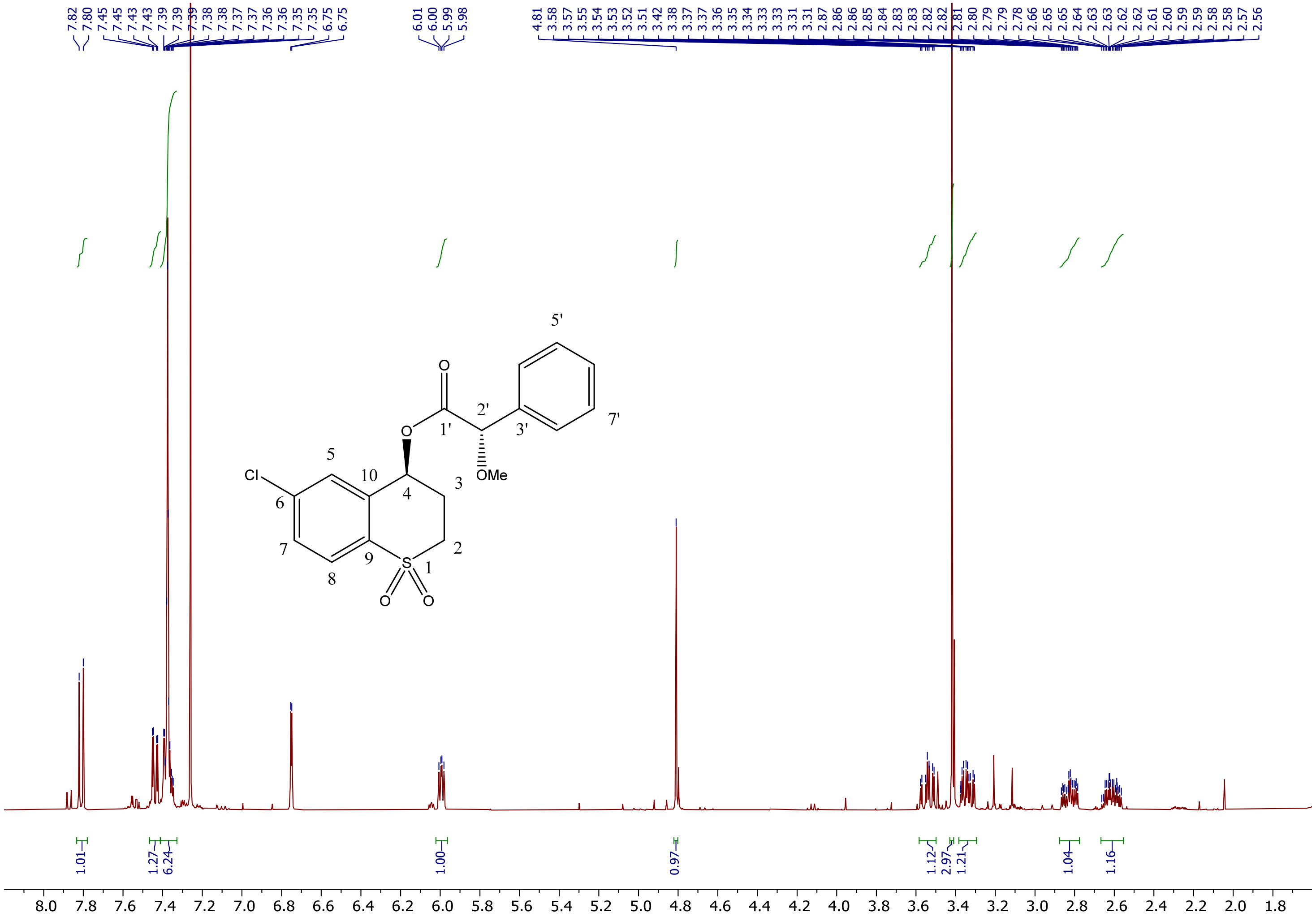

Figure S27. ^{1}H NMR spectrum (400 MHz, $CDCl_3$) of compound ***(S)*-MPA-*(S)*-4a**.

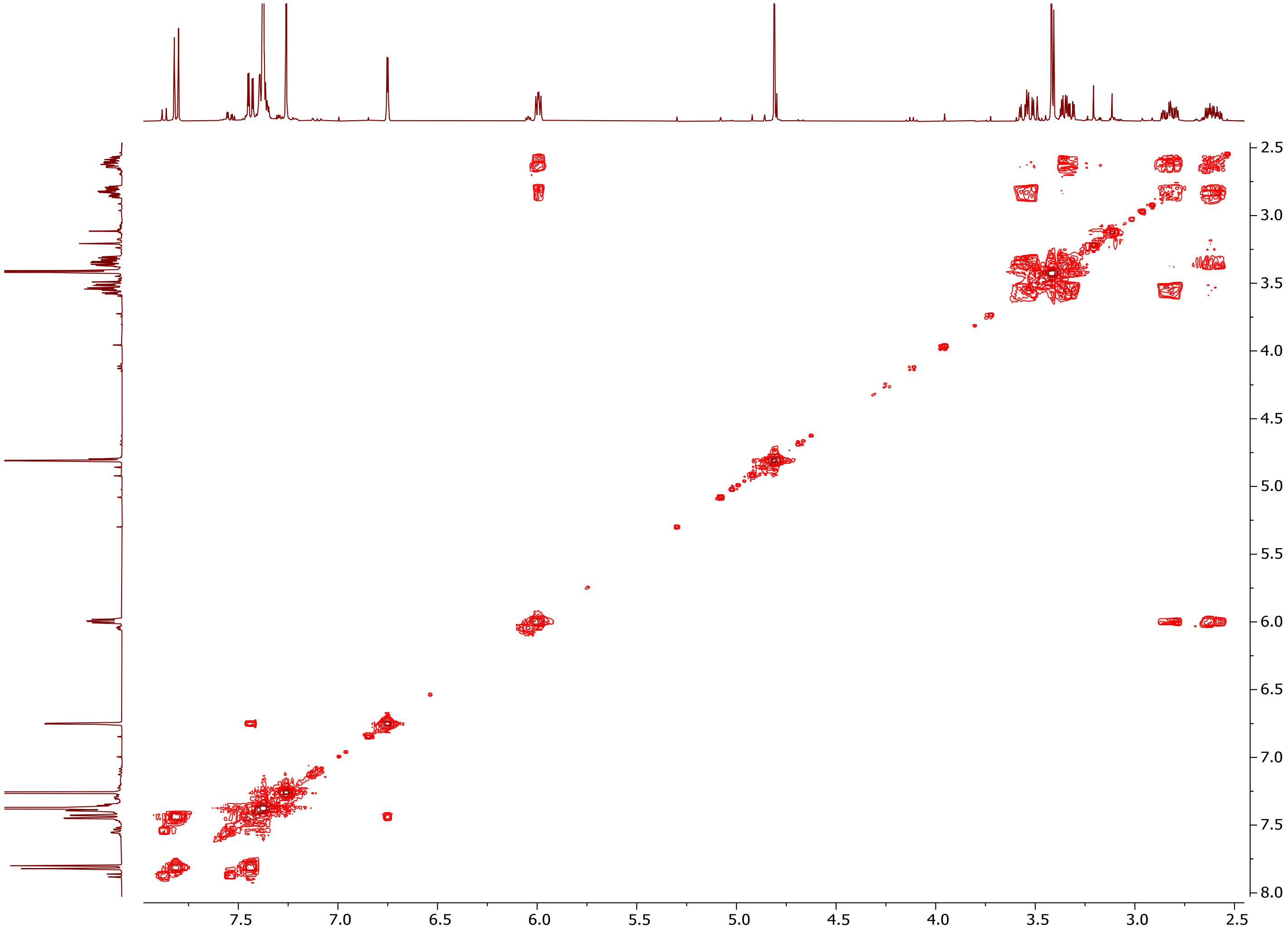

Figure S28. gCOSY spectrum of compound **(*S*)-MPA-(*S*)-4a.**

- **Thiochroman-4-ol (1):**

I

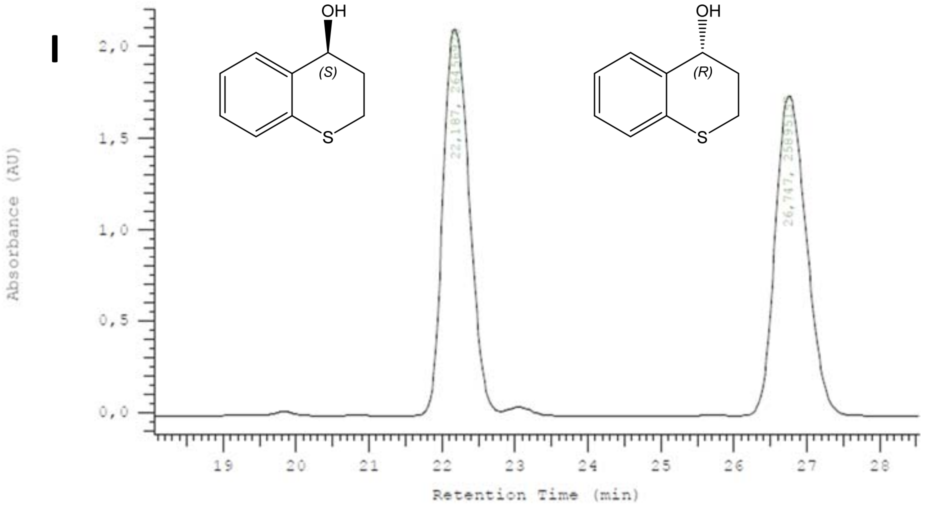

II

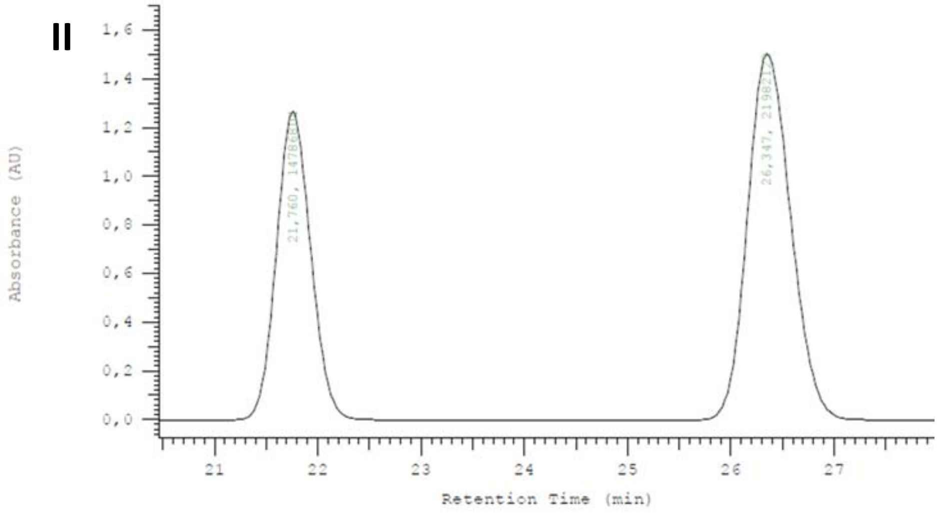

***P. lilacinum* BC17-2**						
**Peak**	**Enantiomer**	**Time (Min)**	**Area**	**Area %**	***ee*(%)**	$[\alpha]_D^{20}$
1	*S*	22.2	26740631	50.8	1.6	-4.1 (c 2.1, $CHCl_3$)
2	*R*	26.7	25895157	49.2		
***E. maritima* BC17**						
**Peak**	**Enantiomer**	**Time (Min)**	**Area**	**Area %**	***ee* (%)**	$[\alpha]_D^{20}$
1	*S*	21.8	14786804	40.2	19.6	+17.4 (c 4.8, MeOH)
2	*R*	26.3	21982123	59.8		

Figure S29. *ee* Determination of thiochroman-4-ol (**1**) from *P. lilacinum* BC17-2 (Chromatogram I) and *E. maritima* BC17 (Chromatogram II).

Determined by chiral HPLC analysis on Chiralcel IB N-5 column (Daicel, Japan, 5 µm, 250 mm × 4.6 mm). Eluent *n*-hexane:*i*-PrOH = 95:5, flow rate 0.6 mL/min, λ = 220 nm.

- ***Syn*-thiochroman-4-ol 1-oxide (3):**

I

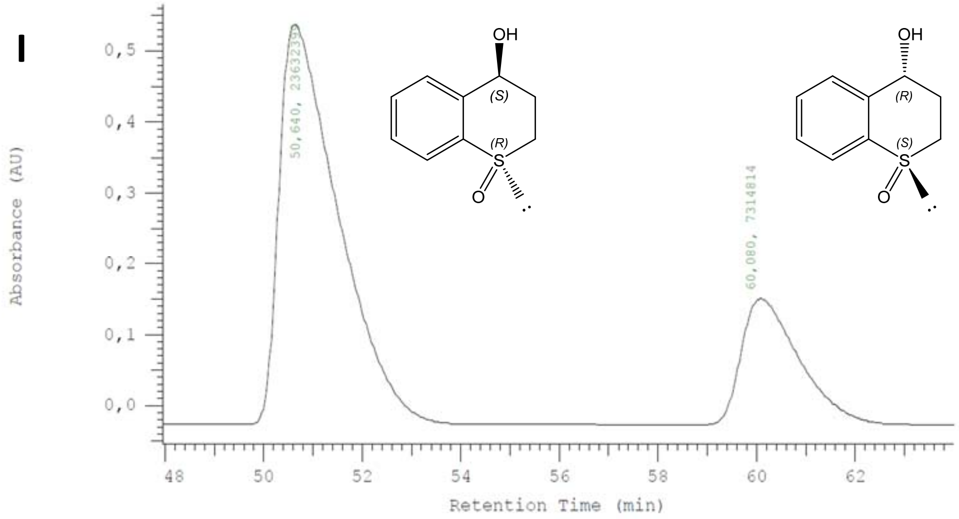

II

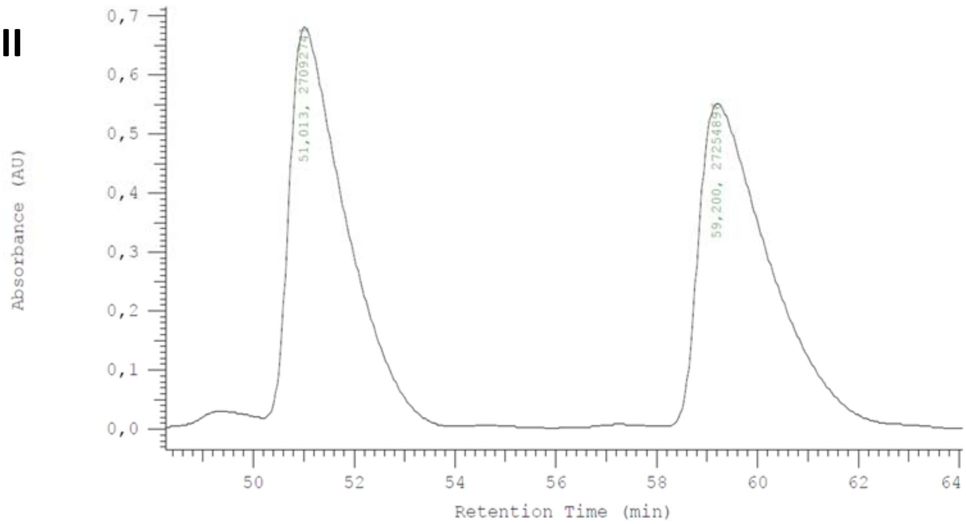

***P. lilacinum* BC17-2**						
**Peak**	**Enantiomer**	**Time (Min)**	**Area**	**Area %**	***ee* (%)**	$[\alpha]_D^{26}$
1	1*R*, 4*S*	50.6	23632399	76.4	52.7	-33.1 (c 0.4, MeOH)
2	1*S*, 4*R*	60.1	7314814	23.6		
***E. maritima* BC17**						
**Peak**	**Enantiomer**	**Time (Min)**	**Area**	**Area %**	***ee* (%)**	
1	*1R, 4S*	51.0	27092741	49.9	0.3	-
2	1*S*, 4*R*	59.2	27254896	50.1		

Figure S30. *ee* Determination of *syn*-thiochroman-4-ol 1-oxide (***syn*-3**) from *P. lilacinum* BC17-2 (Chromatogram I) and *E. maritima* BC17 (Chromatogram II).
Determined by chiral HPLC analysis on Chiralcel IB N-5 column (Daicel, Japan, 5 µm, 250 mm × 4.6 mm). Eluent *n*-hexane:*i*-PrOH = 93:7, flow rate 0.6 mL/min, λ = 220 nm.

- ***Anti*-thiochroman-4-ol 1-oxide (3):**

I

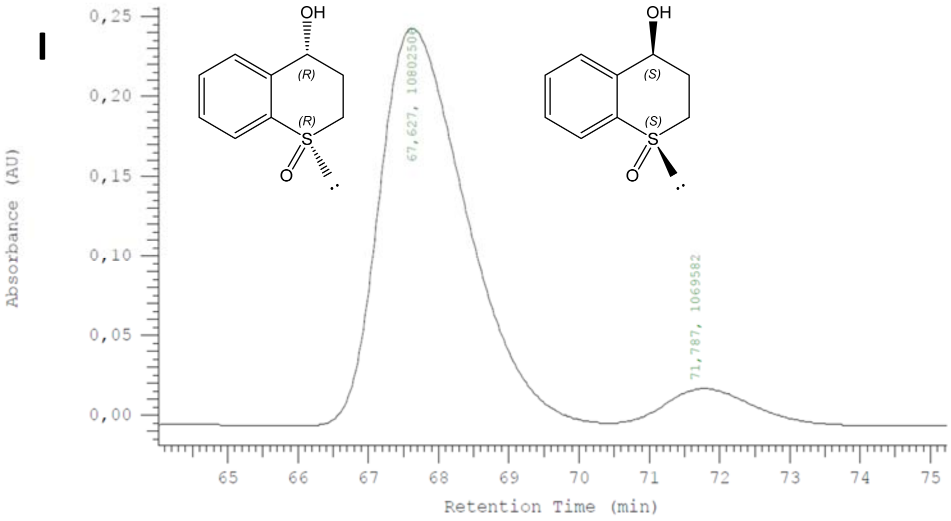

II

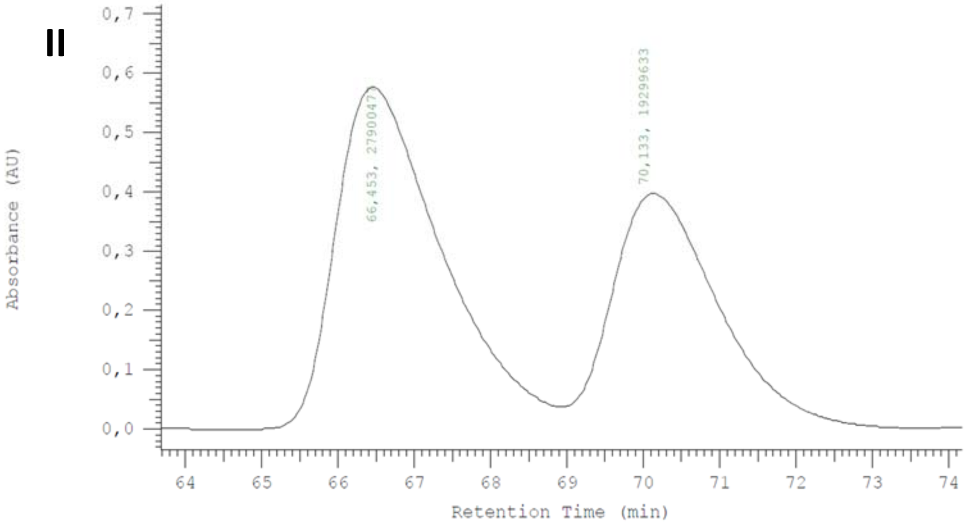

***P. lilacinum* BC17-2**						
**Peak**	**Enantiomer**	**Time (Min)**	**Area**	**Area %**	***ee* (%)**	$[\alpha]_D^{25}$
1	1*R*, 4*R*	67.6	10802506	91.0	82.0	-38.9 (c 0.4, MeOH)
2	1*S*, 4*S*	71.8	1069582	9.0		
***E. maritima* BC17**						
**Peak**	**Enantiomer**	**Time (Min)**	**Area**	**Area %**	***ee* (%)**	
1	1*R*, 4*R*	66.5	27900477	59.1	18.2	-
2	1*S*, 4*S*	70.1	19299633	40.9		

Figure S31. *ee* Determination of *anti*-thiochroman-4-ol 1-oxide (***anti*-3**) from *P. lilacinum* BC17-2 (Chromatogram I) and *E. maritima* BC17 (Chromatogram II).
Determined by chiral HPLC analysis on Chiralcel IB N-5 column (Daicel, Japan, 5 µm, 250 mm × 4.6 mm). Eluent *n*-hexane:*i*-PrOH = 93:7, flow rate 0.6 mL/min, λ = 220 nm.

- **Thiochroman-4-ol 1,1-dioxide (4):**

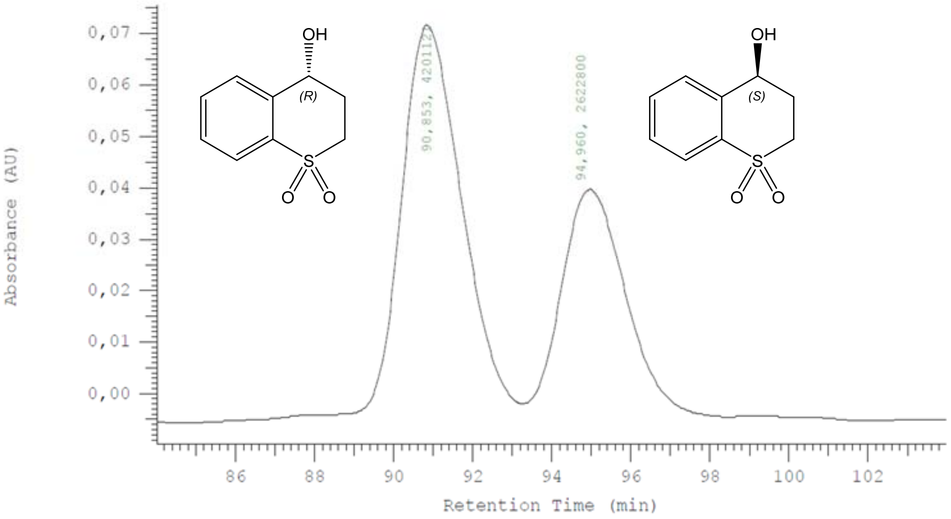

P. lilacinum BC17-2						
**Peak**	**Enantiomer**	**Time (Min)**	**Area**	**Area %**	***ee* (%)**	$[\alpha]_D^{27}$
1	*R*	90.9	4201121	61.6	23.1	-5.9 (c 0.3, $CHCl_3$)
2	*S*	95.0	2622800	38.4		

Figure S32. *ee* Determination of thiochroman-4-ol 1,1-dioxide (**4**) from *P. lilacinum* BC17-2. Determined by chiral HPLC analysis on Chiralcel IB N-5 column (Daicel, Japan, 5 µm, 250 mm × 4.6 mm). Eluent *n*-hexane:*i*-PrOH = 93:7, flow rate 0.6 mL/min, λ = 220 nm.

- **Thiochroman-4-one 1-oxide (5):**

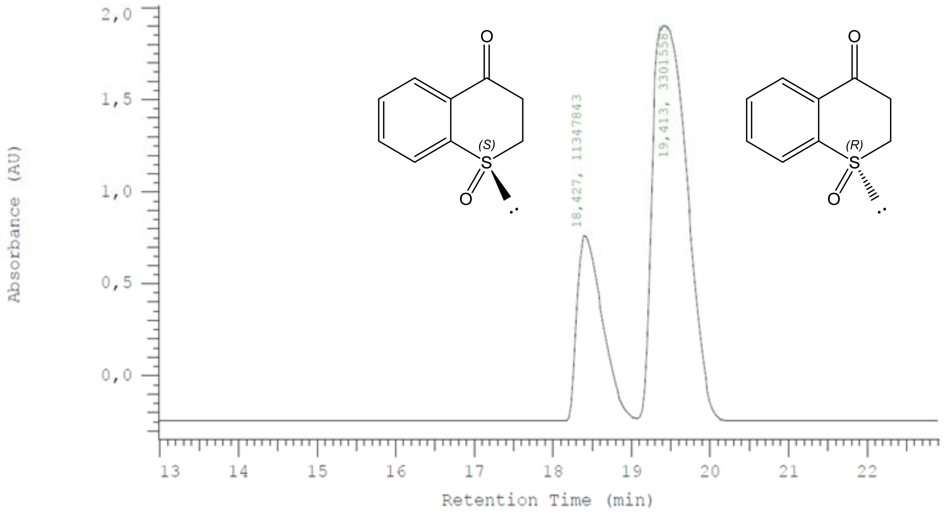

P. lilacinum BC17-2						
Peak	Enantiomer	Time (Min)	Area	Area %	*ee* (%)	$[\alpha]_D^{24}$
1	*S*	18.4	11347843	25.6	48.8	-39.1 (c 0.4, MeOH)
2	*R*	19.4	33015585	74.4		

Figure S33. *ee* Determination of thiochroman-4-one 1-oxide (**5**) from *P. lilacinum* BC17-2. Determined by chiral HPLC analysis on Chiralcel IB N-5 column (Daicel, Japan, 5 µm, 250 mm × 4.6 mm). Eluent *n*-hexane:*i*-PrOH = 70:30, flow rate 0.8 mL/min, λ = 240 nm.

- **6-Chlorothiochroman-4-ol (1a):**

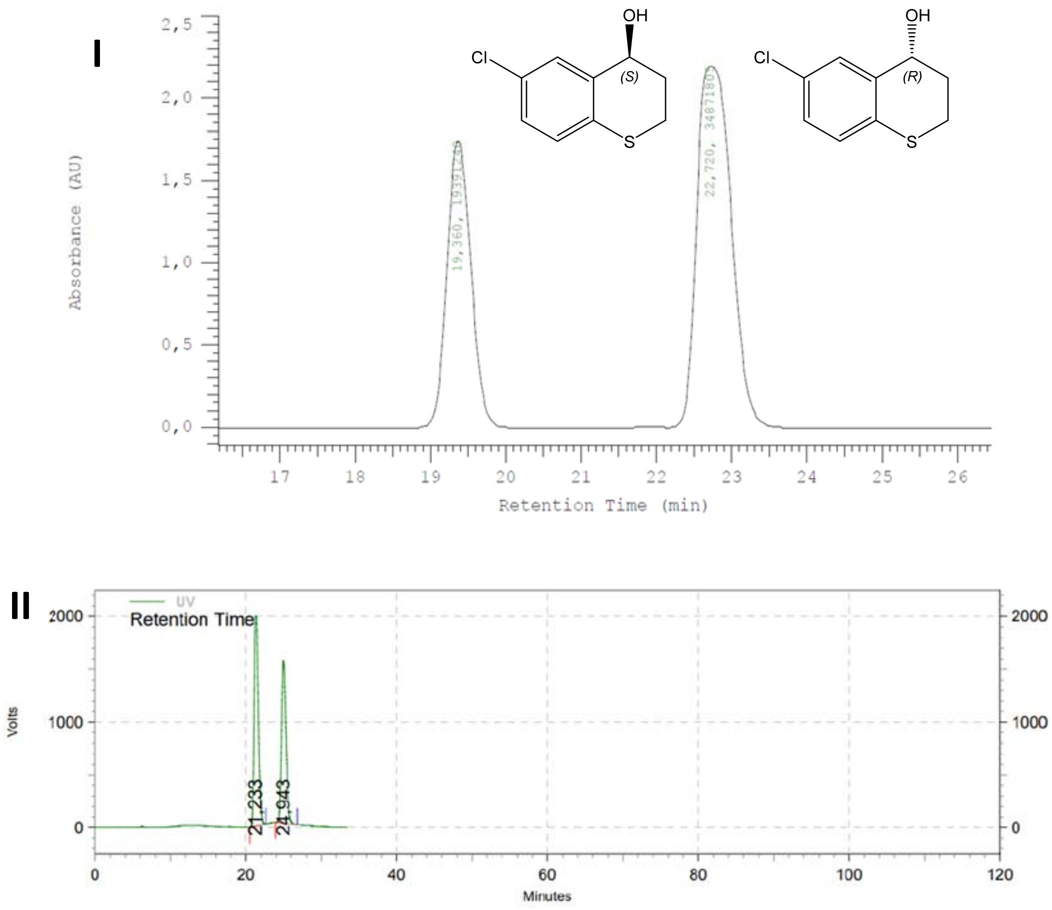

P. lilacinum BC17-2						
Peak	Enantiomer	Time (Min)	Area	Area %	ee (%)	$[\alpha]_D^{21}$
1	*S*	19.4	19391240	35.7	28.5	+22.5 (c 2.4, $CHCl_3$)
2	*R*	22.7	34871805	64.3		
E. maritima **BC17**						
Peak	Enantiomer	Time (Min)	Area	Area %	ee (%)	$[\alpha]_D^{20}$
1	*S*	21.2	27900477	59.1	18.2	-7.9 (c 0.3, MeOH)
2	*R*	24.9	19299633	40.9		

Figure S34. *ee* Determination of 6-chlorothiochroman-4-ol (**1a**) from *P. lilacinum* BC17-2 (Chromatogram I) and *E. maritima* BC17 (Chromatogram II).
Determined by chiral HPLC analysis on Chiralcel IB N-5 column (Daicel, Japan, 5 µm, 250 mm × 4.6 mm). Eluent *n*-hexane:*i*-PrOH = 95:5, flow rate 0.6 mL/min, λ = 250 nm.

- ***Syn*-6-chlorothiochroman-4-ol 1-oxide (*syn*-3a):**

I

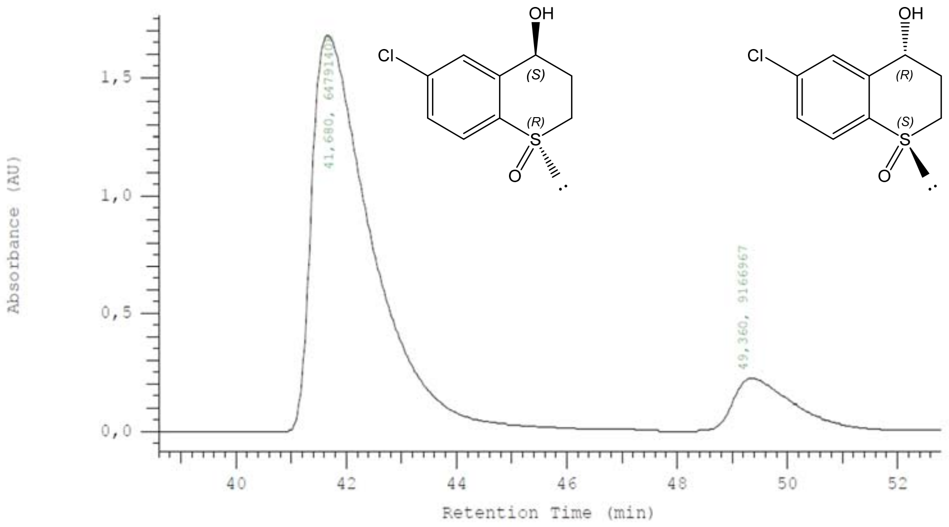

II

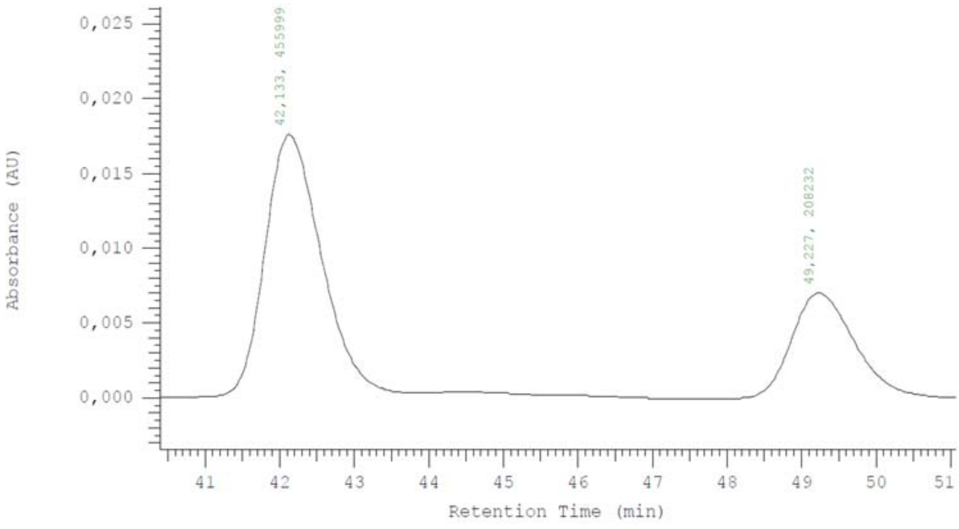

***P. lilacinum* BC17-2**						
**Peak**	**Enantiomer**	**Time (Min)**	**Area**	**Area %**	***ee* (%)**	$[\alpha]_D^{24}$
1	1*R*, 4*S*	41.7	64791408	87.6	75.2	-70.9 (c 0.23, MeOH)
2	1*S*, 4*R*	49.4	9166967	12.4		
***E. maritima* BC17**						
**Peak**	**Enantiomer**	**Time (Min)**	**Area**	**Area %**	***ee* (%)**	$[\alpha]_D^{20}$
1	*1R, 4S*	42.1	455999	68.1	37.3	-19.4 (c 0.42, MeOH)
2	1*S*, 4*R*	49.2	208232	31.9		

Figure S35. *ee* Determination of *syn*-6-chlorothiochroman-4-ol 1-oxide (***syn*-3a**) from *P. lilacinum* BC17-2 (Chromatogram I) and *E. maritima* BC17 (Chromatogram II).
Determined by chiral HPLC analysis on Chiralcel IB N-5 column (Daicel, Japan, 5 µm, 250 mm × 4.6 mm). Eluent *n*-hexane:*i*-PrOH = 93:7, flow rate 0.6 mL/min, λ = 250 nm.

- ***Anti*-6-chlorothiochroman-4-ol 1-oxide (*anti*-3a):**

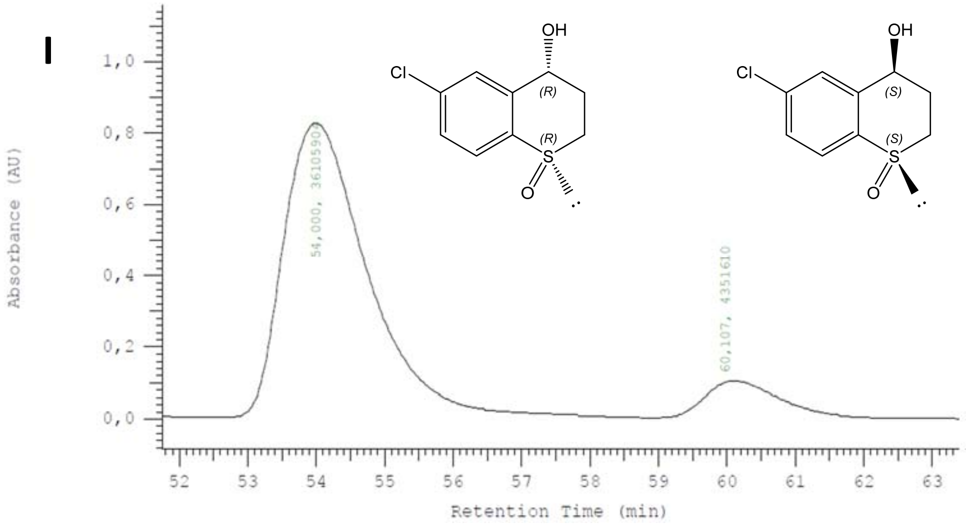

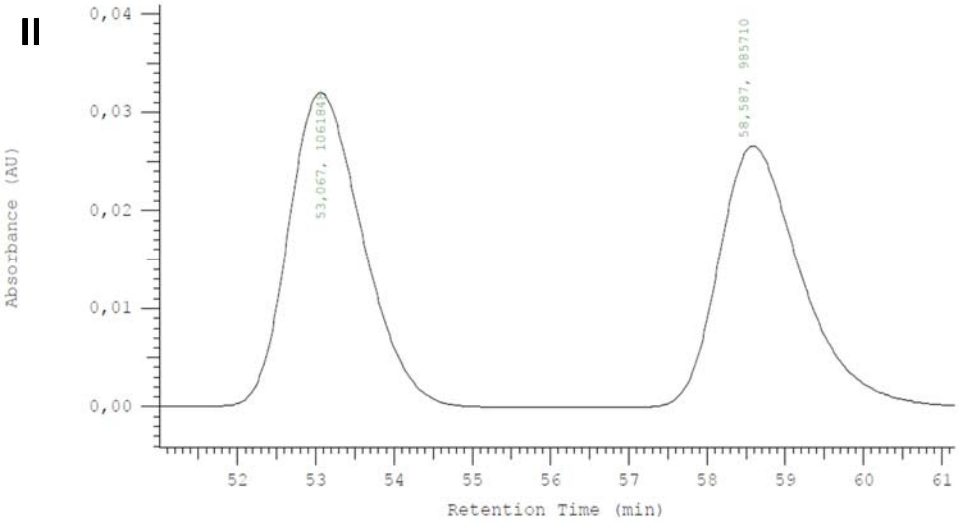

***P. lilacinum* BC17-2**						
**Peak**	**Enantiomer**	**Time (Min)**	**Area**	**Area %**	***ee* (%)**	$[\alpha]_D^{24}$
1	1*R*, 4*R*	54.0	36105904	89.2	78.5	-82.7 (c 1.1, MeOH)
2	1*S*, 4*S*	60.1	4351610	10.8		
***E. maritima* BC17**						
**Peak**	**Enantiomer**	**Time (Min)**	**Area**	**Area %**	***ee* (%)**	$[\alpha]_D^{20}$
1	*1R*, 4*R*	53.1	1061848	51.9	3.7	-10.5 (c 0.42, MeOH)
2	1*S*, 4*S*	58.6	985710	48.1		

Figure S36. *ee* Determination of *anti*-6-chlorothiochroman-4-ol 1-oxide (***anti*-3a**) from *P. lilacinum* BC17-2 (Chromatogram I) and *E. maritima* BC17 (Chromatogram II).
Determined by chiral HPLC analysis on Chiralcel IB N-5 column (Daicel, Japan, 5 µm, 250 mm × 4.6 mm). Eluent *n*-hexane:*i*-PrOH = 93:7, flow rate 0.6 mL/min, λ = 260 nm.

- **6-Chlorohiochroman-4-ol 1,1-dioxide (4a):**

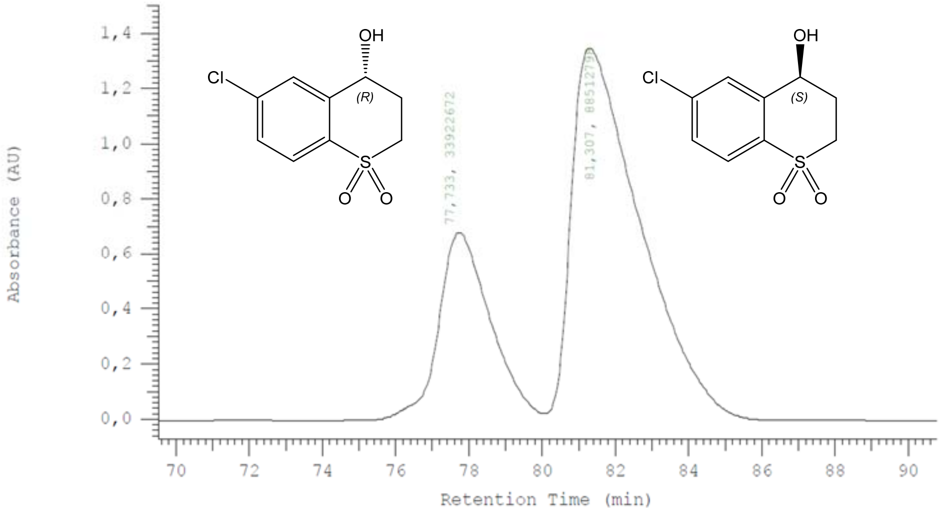

P. lilacinum BC17-2						
**Peak**	**Enantiomer**	**Time (min)**	**Area**	**Area %**	***ee* (%)**	$[\alpha]_D^{27}$
1	*R*	77.7	33922672	27.7	44.6	+10.8 (c 0.3, $CHCl_3$)
2	*S*	81.3	88512798	72.3		

Figure S37. *ee* Determination of 6-chlorothiochroman-4-ol 1,1-dioxide (**4a**) from *P. lilacinum* BC17-2. Determined by chiral HPLC analysis on Chiralcel IB N-5 column (Daicel, Japan, 5 µm, 250 mm × 4.6 mm). Eluent *n*-hexane:*i*-PrOH = 93:7, flow rate 0.6 mL/min, λ = 220 nm.

- **6-Chlorohiochroman-4-one 1-oxide (5a):**

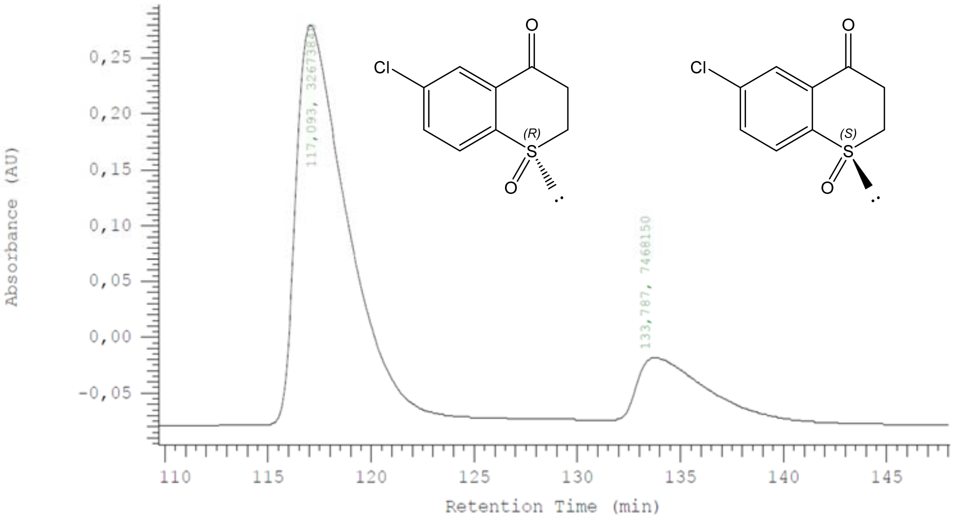

P. lilacinum BC17-2						
Peak	Enantiomer	Time (Min)	Area	Area %	*ee* (%)	$[\alpha]_D^{25}$
1	*R*	117.1	32673840	81.4	62.8	-98.0 (c 0.2, MeOH)
2	*S*	133.8	7468150	18.6		

Figure S38. *ee* Determination of 6-chlorothiochroman-4-one 1-oxide (**5a**) from *P. lilacinum* BC17-2. Determined by chiral HPLC analysis on Chiralcel IB N-5 column (Daicel, Japan, 5 µm, 250 mm × 4.6 mm). Eluent *n*-hexane:*i*-PrOH = 93:7, flow rate 0.6 mL/min, λ = 240 nm.

- **1-(5-chloro-2-(methylthio)phenyl)propane-1,3-diol (6a):**

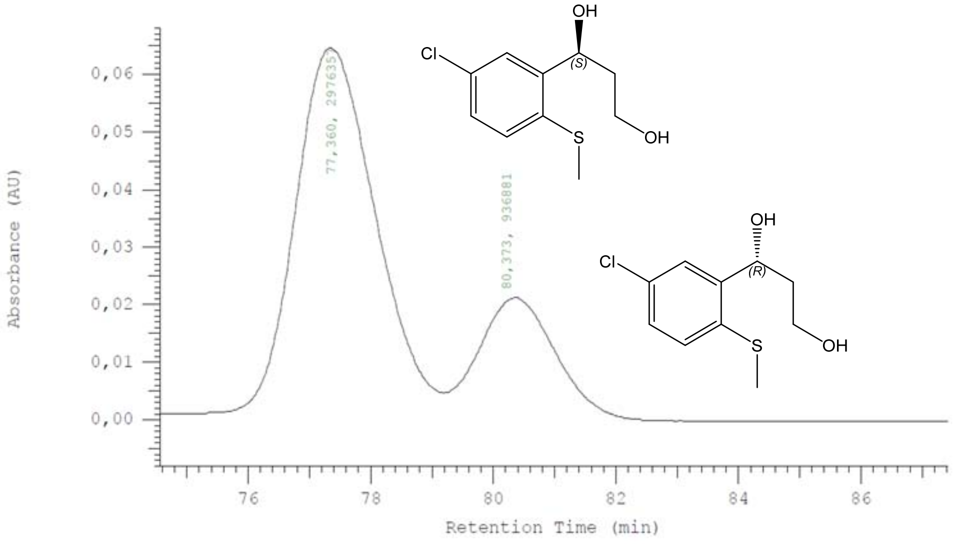

P. lilacinum BC17-2						
Peak	Enantiomer	Time (Min)	Area	Area %	ee(%)	$[\alpha]_D^{25}$
1	*S*	77.4	2976357	76.1	52.1	+10.6 (c 0.06, $CHCl_3$)
2	*R*	80.4	936881	23.9		

Figure S39. *ee* Determination of 1-(5-chloro-2-(methylthio)phenyl)propane-1,3-diol (**6a**) from *P. lilacinum* BC17-2.

Determined by chiral HPLC analysis on Chiralcel IB N-5 column (Daicel, Japan, 5 µm, 250 mm × 4.6 mm). Eluent *n*-hexane:*i*-PrOH = 98:2, flow rate 0.6 mL/min, λ = 260 nm.

- **Thiochroman-4-ol 1-oxide (3):**

I

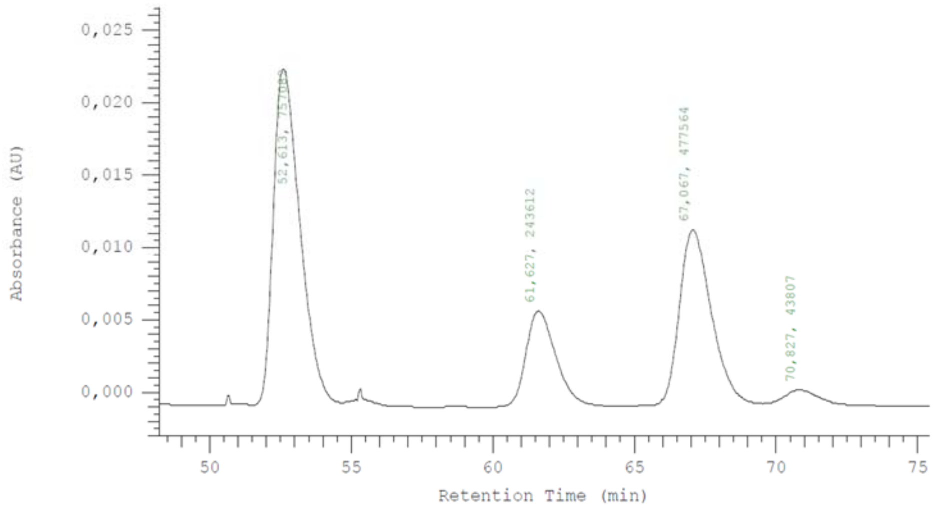

II

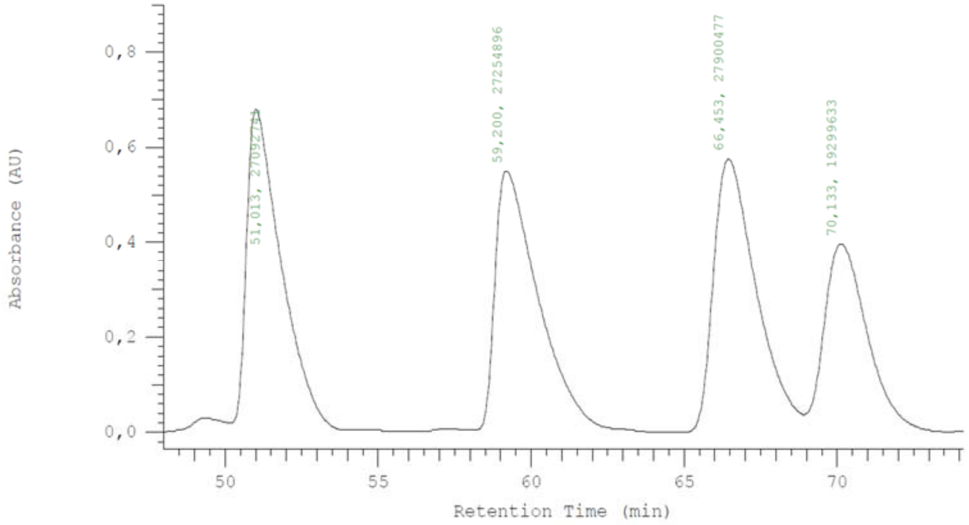

***P. lilacinum* BC17-2**						
**Diastereoisomer**	**Peak**	**Enantiomer**	**Time (Min)**	**Area**	**$A_1 + A_2$**	***ed* (%)**
Syn	1	1*R*, 4*S*	52.6	757082	1000694	31.5
	2	1*S*, 4*R*	61.6	243612		
Anti	3	1*R*, 4*R*	67.1	477564	521371	
	4	1*S*, 4*S*	70.8	43807		
***E. maritima* BC17**						
**Diastereoisomer**	**Peak**	**Enantiomer**	**Time (Min)**	**Area**	**$A_1 + A_2$**	***ed* (%)**
Syn	1	1*R*, 4*S*	51.0	27092741	54347637	7.0
	2	1*S*, 4*R*	59.2	27254896		
Anti	3	1*R*, 4*R*	66.5	27900477	47200110	
	4	1*S*, 4*S*	70.1	19299633		

Figure S40. *ed* Determination of thiochroman-4-ol 1-oxide (**3**) diastereoisomers from *P. lilacinum* BC17-2 (Chromatogram I) and *E. maritima* BC17 (Chromatogram II).
Determined by chiral HPLC analysis on Chiralcel IB N-5 column (Daicel, Japan, 5 µm, 250 mm × 4.6 mm). Eluent *n*-hexane:*i*-PrOH = 95:5, flow rate 0.6 mL/min, λ = 220 nm.

- **6-Chlorothiochroman-4-ol 1-oxide (3a):**

I

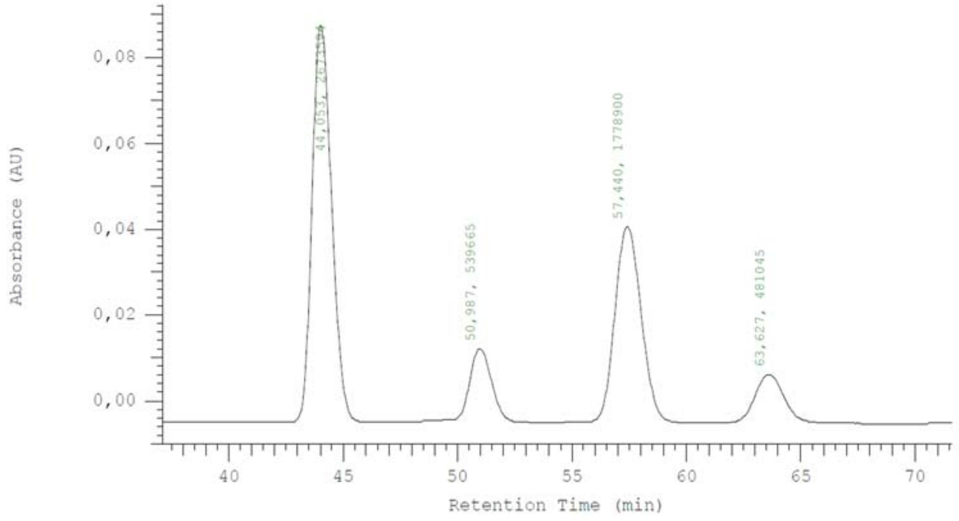

II

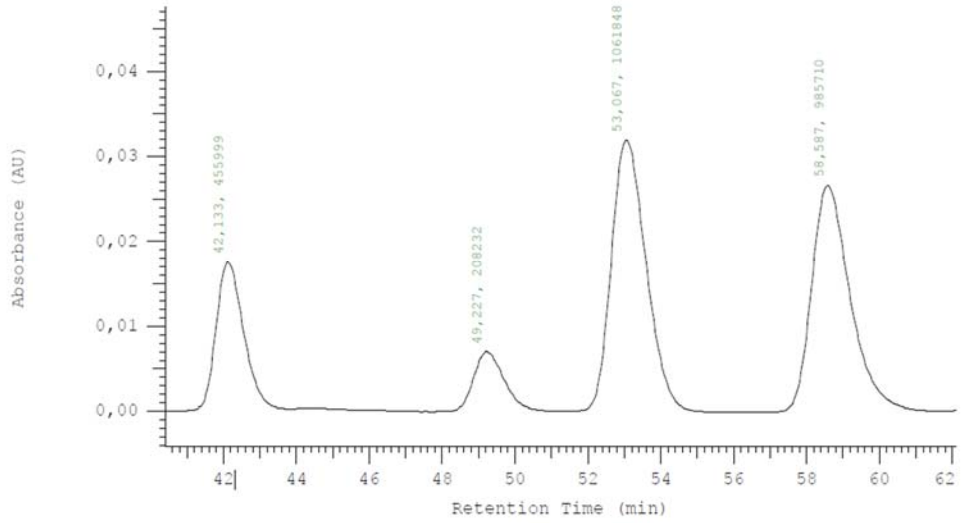

P. lilacinum BC17-2						
**Diastereoisomer**	**Peak**	**Enantiomer**	**Time (Min)**	**Area**	**$A_1 + A_2$**	***ed* (%)**
Syn	1	1*R*, 4*S*	44.1	2673594	3213259	17.4%
	2	1*S*, 4*R*	51.0	539665		
Anti	3	1*R*, 4*R*	57.4	1778900	2259945	
	4	1*S*, 4*S*	63.6	481045		
***E. maritima* BC17**						
**Diastereoisomer**	**Peak**	**Enantiomer**	**Time (Min)**	**Area**	**$A_1 + A_2$**	***ed* (%)**
Syn	1	*1R*, 4*S*	42.1	455999	664231	51.0%
	2	1*S*, 4*R*	49.2	208232		
Anti	3	1*R*, 4*R*	53.1	1061848	2047558	
	4	1*S*, 4*S*	58.6	985710		

Figure S41. *ed* Determination of 6-chlorothiochroman-4-ol 1-oxide (**3a**) diastereoisomers from *P. lilacinum* BC17-2 (Chromatogram I) and *E. maritima* BC17 (Chromatogram II).
Determined by chiral HPLC analysis on Chiralcel IB N-5 column (Daicel, Japan, 5 µm, 250 mm × 4.6 mm). Eluent *n*-hexane:*i*-PrOH = 95:5, flow rate 0.6 mL/min, λ = 260 nm.

MIC (µg/mL)			
**Compound**	***S. aureus* ATCC 29213**	***E. coli* ATCC25922**	***C. albicans* HPM-1922816**
Thiochroman-4-ol (**1**)	>256	256	>128
6-Chlorothiochroman-4-ol (**1a**)	>256	256	>128
Thiochroman-4-one (**2**)	>256	256	>128
6-Chlorothiochroman-4-one (**2a**)	>256	256	>128
syn-Thiochroman-4-ol 1-oxide (***syn*-3**)	>256	>256	>128
anti-Thiochroman-4-ol 1-oxide (***anti*-3**)	>256	>256	>128
syn-6-Chlorohiochroman-4-ol 1-oxide (***syn*-3a**)	>256	256	>128
anti-6-Chlorohiochroman-4-ol 1-oxide (***anti*-3a**)	>256	256	>128
Thiochroman-4-ol 1,1-dioxide (**4**)	>256	>256	>128
6-Chlorothiochroman-4-ol 1,1 dioxide (**4a**)	>256	>256	>128
Thiochroman-4-one 1-oxide (**5**)	>256	256	>128
6-Chlorothiochroman-4-one 1-oxide (**5a**)	>256	256	>128

Figure S42. *In vitro* antimicrobial assays.
